# Supplementary figures and images for: A shared ancient enhancer element differentially regulates the bric-a-brac tandem gene duplicates in the developing Drosophila leg
Source: PLoS Genet. 2022 Mar 16;18(3):e1010083. doi: 10.1371/journal.pgen.1010083 (PMC8959175; doi:10.1371/journal.pgen.1010083)

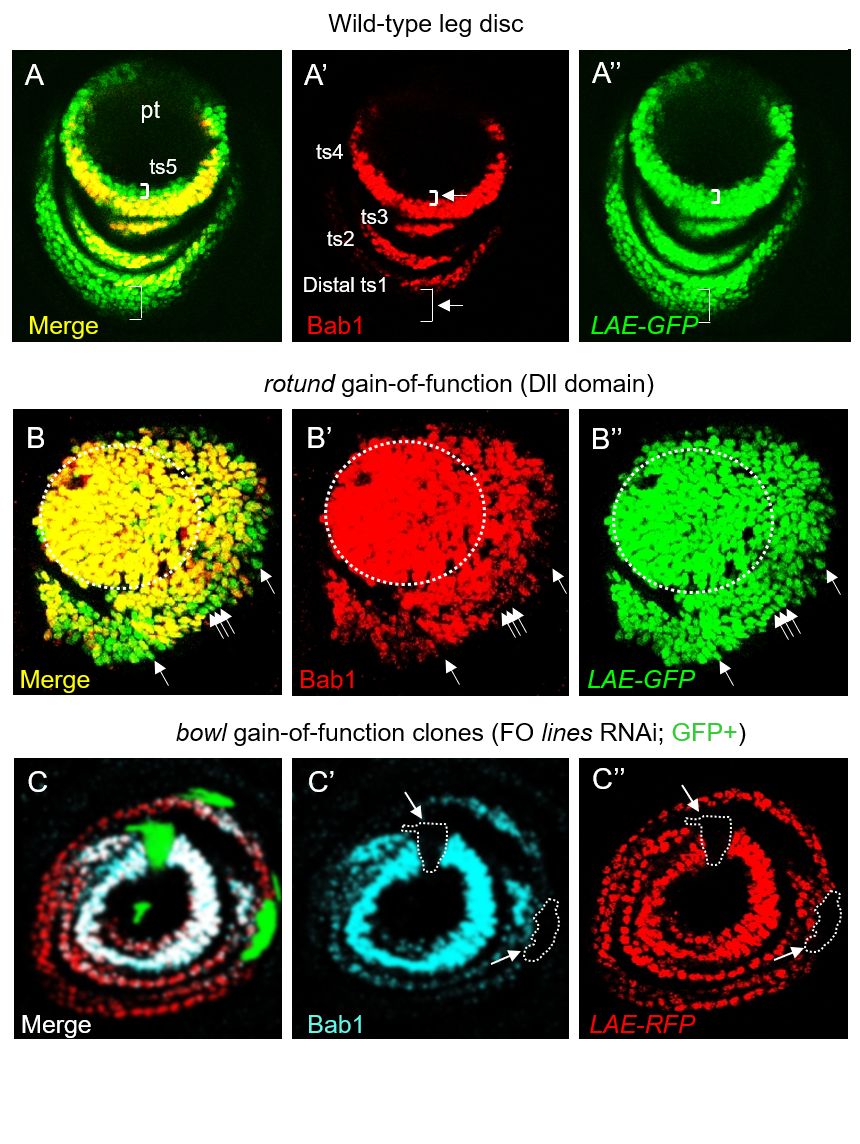

Supplement: S1 Fig — (A) The bab1 paralog is expressed in a subset of LAE-GFPZH2A (bab2)-expressing cells, both proximally and distally within the developing tarsus. Merged Bab1 (red) immunostaining and GFP fluorescence (green) as well as each marker in isolation in (A’) and (A”), respectively, are shown for a wild-type L3 leg disc expressing LAE-GFPZH2A (medial confocal view). Positions of LAE-GFPZH2A (bab2)-expressing ts1-5 cells and of the non-expressing pre-tarsal (pt) cells are indicated in (A) and (A’). Brackets indicate paralog-specific expression in bab2-expressing (GFP+) ts1 and ts5 cells, as detected as green- instead of yellow-colored cells in (A) (see also white arrows in (A’)). Of note, bab1 is only expressed in distal ts1 cells, while LAE-GFPZH2A (bab2) expression extends proximally. (B) Rotund TF gain-of-function within the developing Dll-expressing cells differentially activates the bab gene paralogs along the P-D leg axis. Merged Bab1 (red) immunostaining and GFP (green) fluorescence, as well as each marker in isolation in (B’) and (B”), respectively, are shown for a leg disc dissected from a L3 larvae harboring both UAS-Rn and DllEM212-Gal4 transgenes. Contrary to a distal domain (circled with a dashed line) in which both bab1 and LAE-GFPZH2A (bab2) are strictly co-expressed, many proximalmost Dll-expressing GFP+ cells neither activate bab1 (some are indicated by white arrows). (C) Ectopic Bowl TF stabilization, through clonal Lines protein depletion, is sufficient to down-regulate both bab1 and LAE-GFPZH2A (bab2) expression. Merged Bab1 (cyan) immunostaining, RFP (red) and GFP (green) fluorescence, as well as the two former markers in isolation in (C’) and (C”), respectively, are shown for a L3 leg disc expressing LAE-RFPZH2A. Flip-out (FO) mitotic clones are detected through GFP expression in (C), and are circled with dashed lines in (C’) and (C”). Within the developing tarsus Bowl stabilization leads to cell-autonomous repression of both bab1 and LAE-RFPZH2A (bab2). [file pgen.1010083.s001.tif]

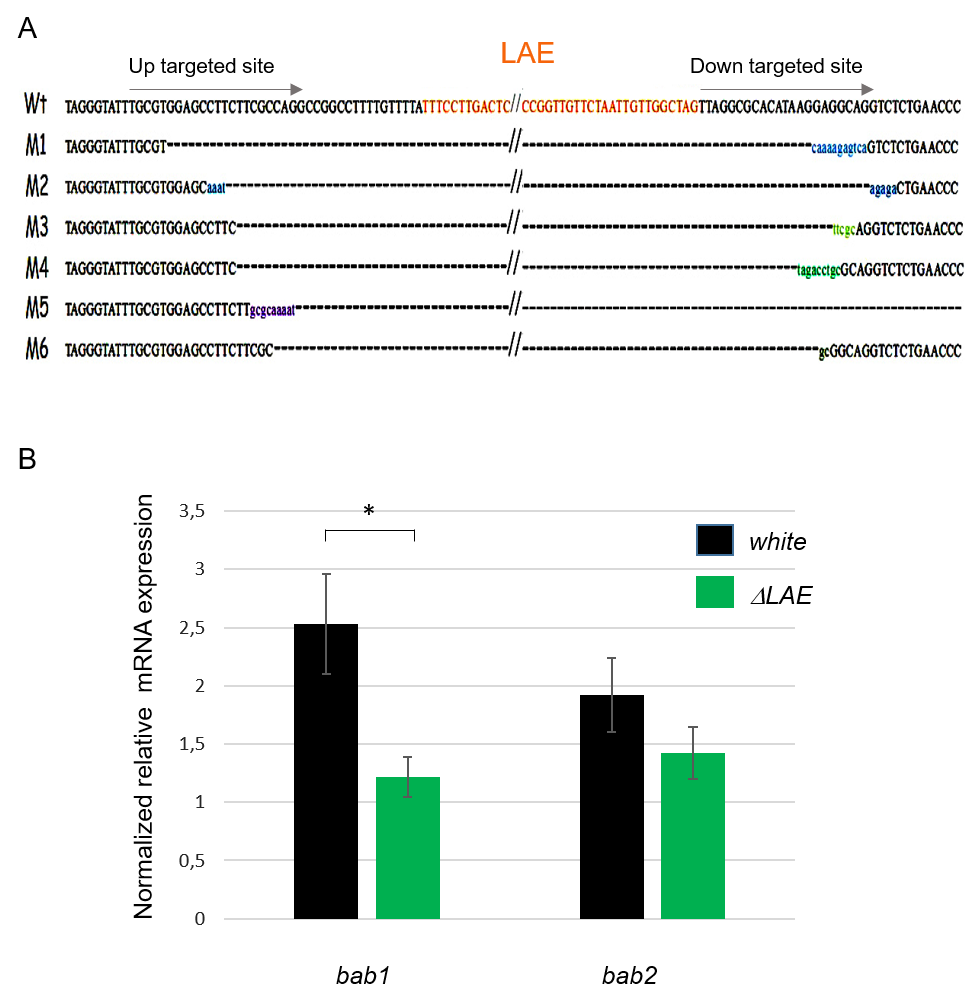

Supplement: S2 Fig — (A) Targeted deletion of the LAE with CRIPSR/Cas9 genome editing. The sequences flanking LAE from the wild-type (Wt) and six deleted chromosomes (M1-6) are shown. LAE sequences are depicted in orange while exogenous sequences in mutant chromosomes are indicated by distinctly-colored lower case letters (unmodified nucleotides are upper case ones). (B) Overall bab1-2 expression from wild-type and homozygous babΔLAE L3 leg discs, as determined from reverse transcription quantitative PCR analyses. mRNA levels are normalized from expression of three housekeeping genes: Rpl32, Mlc-c and Gpdh1. Results show the mean and the standard error of the mean of 4 independent experiments (Wilcoxon test p value < 0.05 is indicated by *). (TIF) [file pgen.1010083.s002.tif]

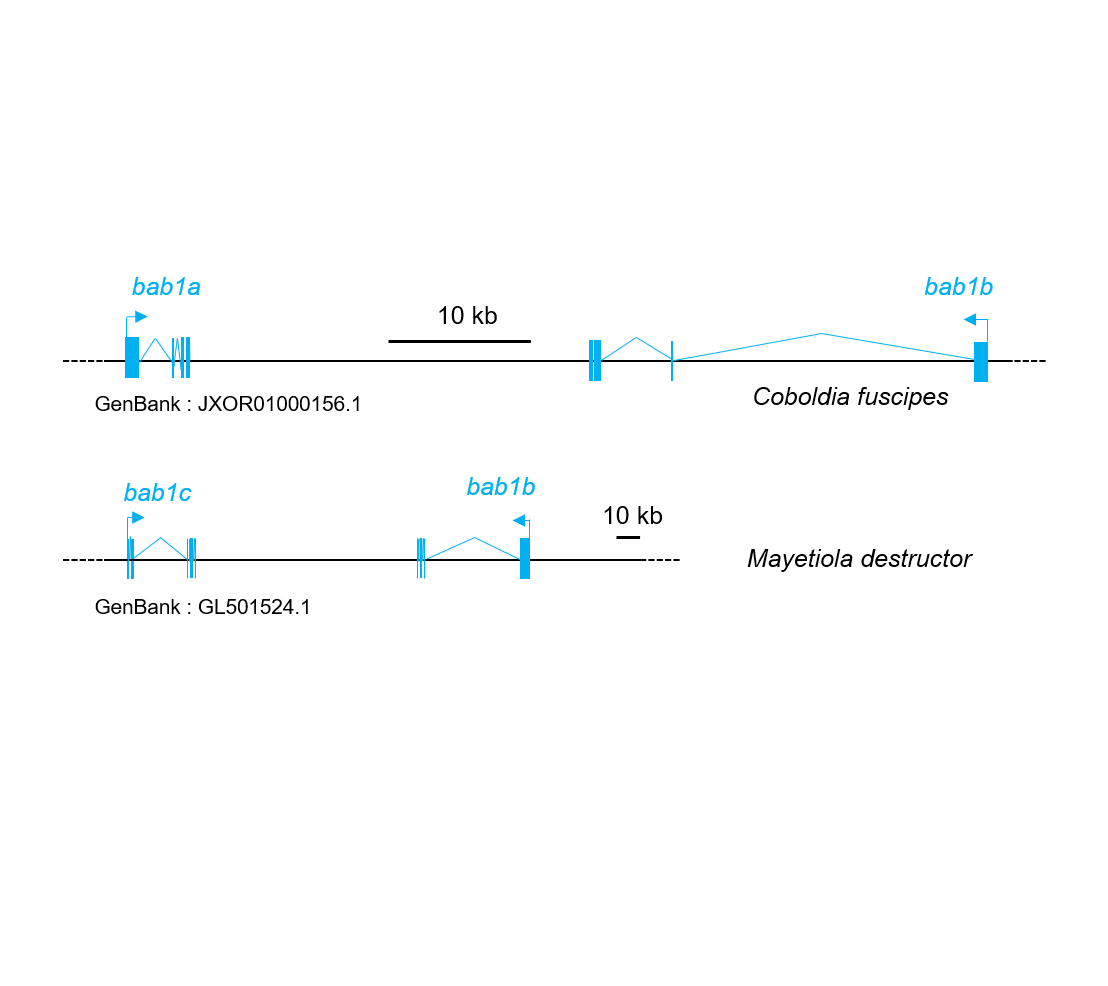

Supplement: S3 Fig — bric-a-brac paralogs from the fungus gnat Coboldia fuscipes (Psychodomorpha) and the gall midge Mayetiola destructor (Bibionomorpha), are shown. GenBank identifiers of the corresponding genomic sequences are indicated. (TIF) [file pgen.1010083.s003.tif]

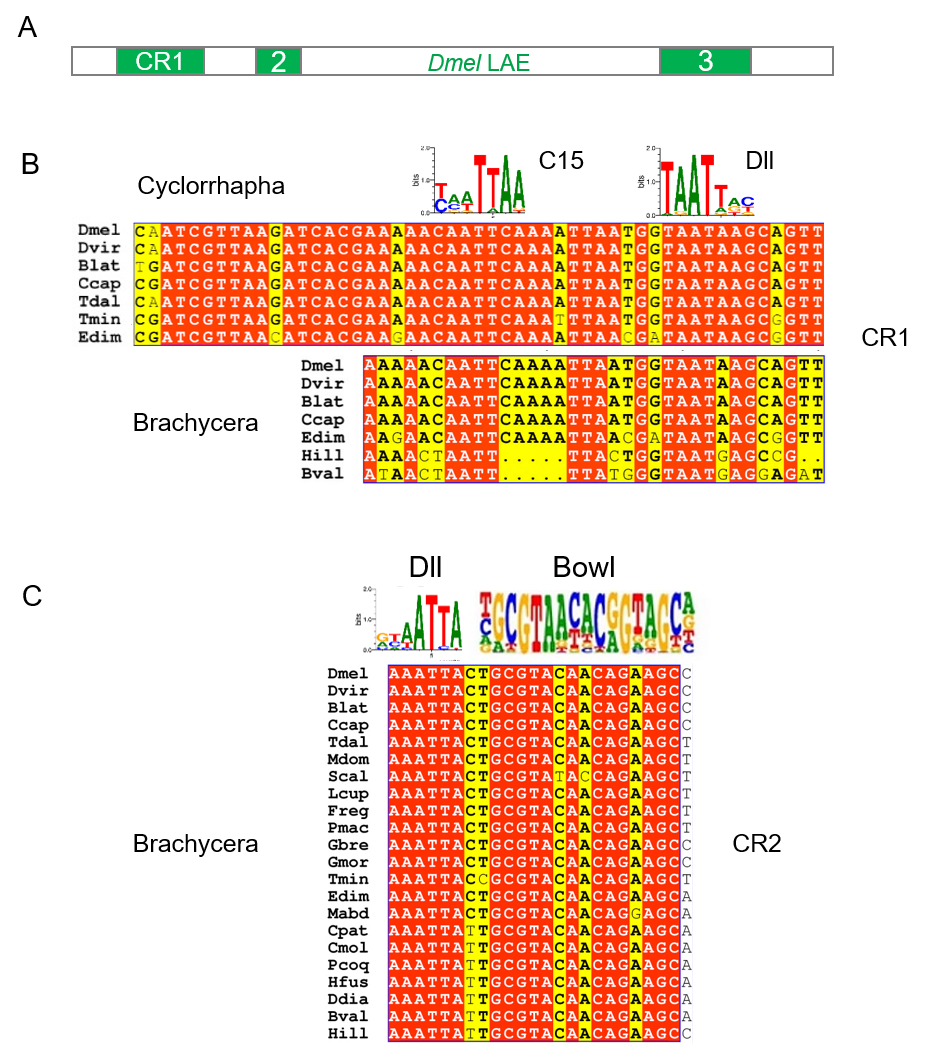

Supplement: S4 Fig — (A) Structural conservation of the Dmel LAE enhancer among Drosophilidae. The locations of CR1-3 sequences, conserved among 12 reference drosophilid genomes, are boxed in green. (B-C) Alignments of brachyceran CR1 (B) and CR2 (C) sequences are shown. The four-letter species abbreviations are listed in S1 Data. Strictly conserved positions are indicated by white characters on a red background while partially ones conserved (>50%) are in black characters on a yellow background. The sequence LOGOs for the evolutionarily-conserved C15, Dll and Bowl binding sites are indicated above the aligned sequences. (TIF) [file pgen.1010083.s004.tif]

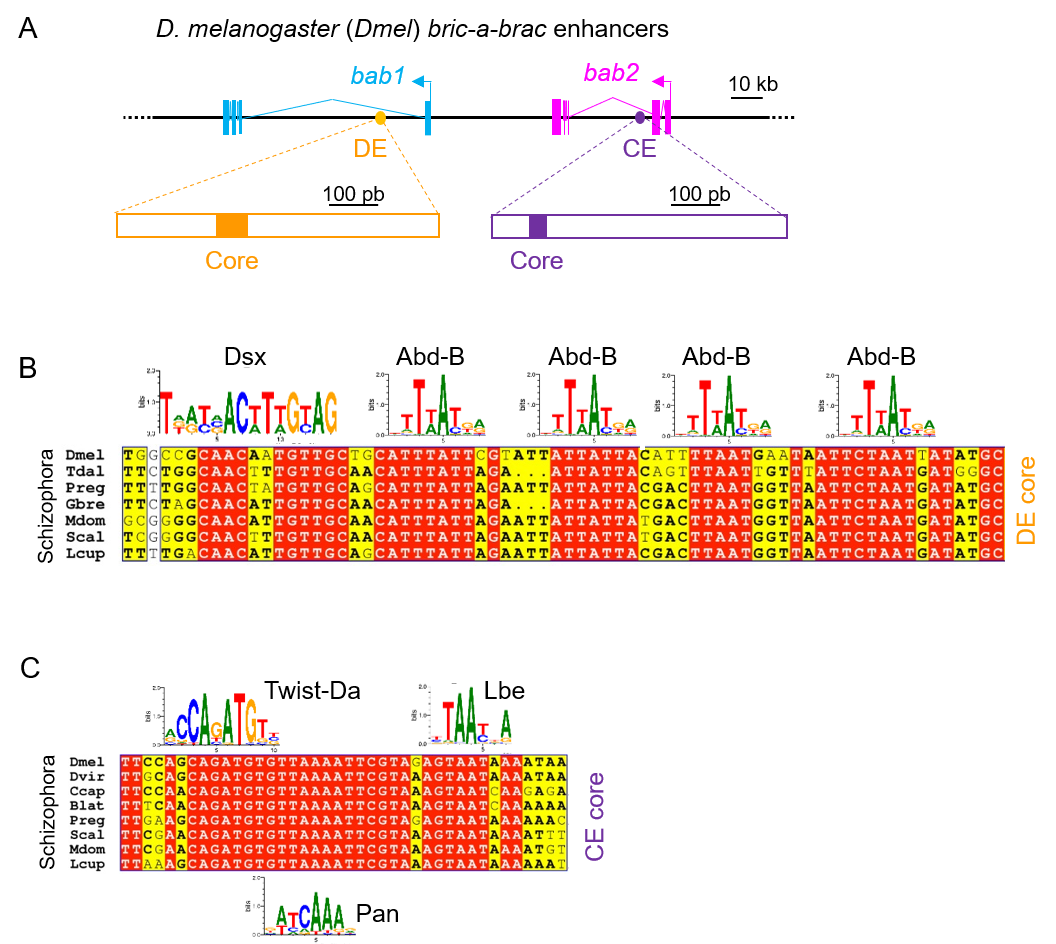

Supplement: S5 Fig — (A) Schematic view of the DE and CE enhancers within the Dmel bab locus. The tandem bab1 (blue) and bab2 (magenta) transcription units are depicted as in Fig 4A. Positions of the evolutionarily-conserved cores within the cardiac CE and abdominal DE sequences are shown in beneath. (B-C) Evolutionary conservation of CE (B) and DE (C) core sequences among schizophorans. The four-letter species abbreviations are listed in S1 Data. Strictly conserved positions are indicated by white characters on a red background while partially conserved ones (>50%) are in black characters on a yellow background. The sequence LOGOs for bona fide (Dsx and Abd-B) or predicted (Twist-Da, Lbe and Pan) transcription factor binding sites are shown above or below the alignments. (TIF) [file pgen.1010083.s005.tif]

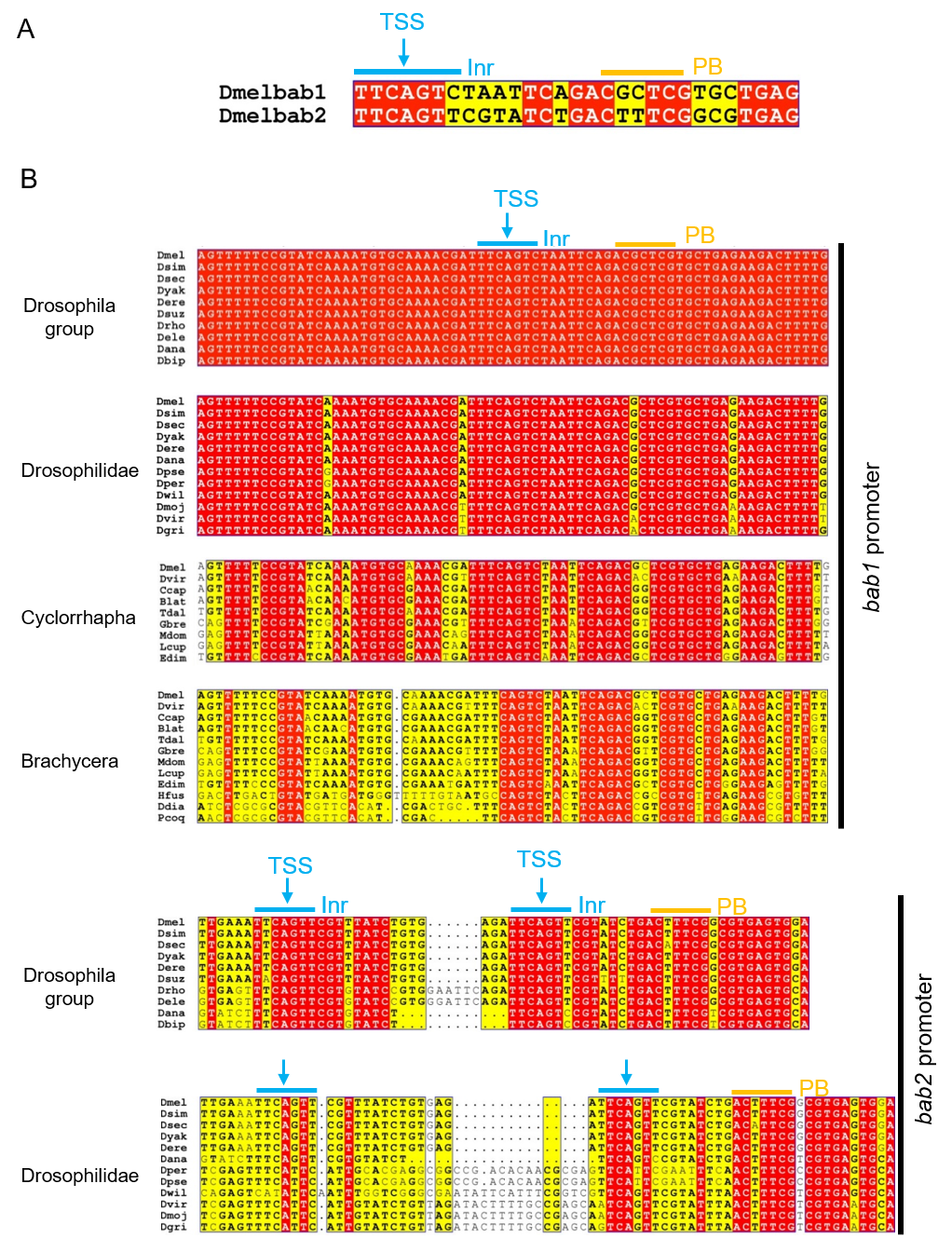

Supplement: S6 Fig — (A) Sequence homology between the Dmel twin bab gene promoters. Positions of initiator (Inr) and pause button (PB) sequences are indicated above the aligned sequences. Transcription start site (TSS) is indicated by a vertical arrow. (B-C) Evolutionary conservation of bab1 (B) and bab2 (C) promoter sequences, among selected dipteran lineages (as indicated on the left side). The four-letter species abbreviations are listed in S1 Data. Strictly conserved positions are indicated by white characters on a red background while partially conserved ones are in black characters on a yellow background. Inr, PB and TSS locations are depicted as in (A). (TIF) [file pgen.1010083.s006.tif]
