# Supplementary material for: A shared ancient enhancer element differentially regulates the bric-a-brac tandem gene duplicates in the developing Drosophila leg
Source: PLoS Genet. 2022 Mar 16;18(3):e1010083. doi: 10.1371/journal.pgen.1010083 (PMC8959175; doi:10.1371/journal.pgen.1010083)

Supplementary data

Four letter abbreviations for investigated species page 2

Predicted sequences for BTB-BabCD proteins pages 3-20

Bab1 sequence conservation among cyclorrhaphans pages 21-22

Bab2 sequence conservation among cyclorrhaphans pages 23-24

Sequence conservation between Bab1/2 paralogs pages 25-29

Sequence conservation between paralogous Bab1/2 proteins among cyclorrhaphans. The four-letter species abbreviations are as listed below (page 2). Strictly conserved amino-acid residues are indicated by white characters on a red background while partially conserved ones are in black characters on a yellow background. Locations of the strongly-conserved BTB and BabCD domains are indicated along the right side (see black lines).

Enhancer sequence conservation among Drosophilidae pages 30-39

Conservation among twelve reference drosophilids of *D. melanogaster* LAE, CE, AE and DE sequences. The four-letter Drosophilidae species abbreviations are as listed below (page 2). Sequence LOGOs of (predicted) binding sites for the Dll, Bowl, C15, Rn, Pan, Lbe, Twist, Abd-B and Dsx transcription factors are depicted above or below the alignments.

Four letter abbreviations for investigated species

Dmel: Drosophila melanogaster						
Dsim: Drosophila simulans						
Dsec: Drosophila sechellia						
Dyak: Drosophila yakuba						
Dere: Drosophila erecta						
Dsuz: Drosophila suzukii						
Drho: Drosophila rhopaloa						
Dele: Drosophila elegans	Drosophilidae					
Dbip: Drosophila bipectinata						
Dana: Drosophila ananassae						
Dper: Drosophila persimilis						
Dpse: Drosophila pseudoobscura		Acalyptratae				
Dwil: Drosophila willistoni						
Dvir: Drosophila virilis						
Dmoj: Drosophila mojavensis				Cyclorrhapha	Eremoneura	
Dgri: Drosophila grimshawi						
Tmin: Themira minor			Schizophora			
Tdal: Teleopsis dalmanni						
Blat: Bactrocera latifrons						
Ccap : Ceratitis capitata						
Mdom: Musca domestica						Brachycera
Scal: Stomoxys calcitrans						
Lcup: Lucilia cuprina						
Preg: Phormia regina						
Chom: Cochliomyia hominivorax		Calyptratae				
Pmac: Paykullia maculata						
Gbre: Glossina brevipalpis						
Gmor: Glossina morsitans						
Edim: Eristalis dimidiata			Aschiza			
Mabd: Megaselia abdita						
Cpat: Condylostylus patibulatus			Empidoidea			
Pcoq: Proctacanthus coquilletti						
Ddia: Dasypogon diadema			Asiloidea			
Hfus: Holcocephala fusca					Orthorrhapha	
Hill: Hermetia illucens			Stratiomyomorpha			
Mdes: Mayetiola destructor			Binionomorpha			
Cfus: Coboldia fuscipes						
Ppat: Phlebotomus papatasi			Psychodomorpha			
Llon: Lutzomyia longipalpis						Nematocera
Agam: Anopheles gambiae						
Aaeg: Aedes aegypti			Culicomorpha			
Dpul: Daphnia pulex	Crustacea					

>Drosophila_melanogaster_Bab1_Brachycera_Cyclorrhapha_Schizophora_
Acalyptratae_Drosophilidae
MASAQAETNVGLASEQGPVAQRQRKGTGSGADSPKSNRSSPTQQEEKRIKSEDRTSPTGG
AKDEDKESQGHAVAGGGGSSPVSSPQGRSSSVASPSSSSQQFCLRWNNYQTNLTTIFDQL
LQNECFVDVTLACDGRSMKAHKMVLSACSPYFQTLLAETPCQHPIVIMRDVNWSDLKAIV
EFMYRGEINVSQDQIGPLLRIAEMLKVRGLADVTHMEAATAAAAAASSERMPSSPKESTS
TSRTEHDREREAEELLAFMQPEKKLRTSDWDPAELRLSPLERQQGRNVRKRRWPSADTIF
NPPAPPSPLSSLIAAERMELEQKERERQRDCSLMTPPPKPPMSSGSTVGATRRLETAIHA
LDMPSPAATPGPLSRSSRPHSQSPQQQQAQQQGQLPLPLPLHPHHHASPAPHPSQTAGSA
HHPASPAGDSRFPLGPAAAMAAARELSGLGPGPSAEPRLPPPPPHHHGGGGVGGGGVGGG
GAGGVGSGGGSSLADDLEIKPGIAEMIREEERAKMMENSHAWMGATGSTLAADSYQYQLQ
SMWQKCWNTNQNLMHHMRFRERGPLKSWRPETMAEAIFSVLKEGLSLSQAARKYDIPYPT
FVLYANRVHNMLGPSIDGGPDLRPKGRGRPQRILLGIWPDEHIKGVIKTVVFRDTKDIKD
ESLAAHMPPYGRHSPAFPLQDLPLSYPGASGALAGAPSSMACPNGSGPQTGVGVAGEQHM
SQETAAAVAAVAHNIRQQMQMAAVPPGLFNLPPHPGVGGGVGNVPGAAGGRASISPALSS
GSGPRHAPSPCGPAGLLPNLPPSMAVALHHQQQQAAHHHMQQLHLQQQQAHLHHHQQQQ
QQQQQQHHQGGHQVAHKSGFGASSSSSASSSSMGQHHAPKAKSSPLRSETPRLHSPLGDL
GLDMASYKREFSPSRLFAEDLAELVGASVSSSSSSAAAATAPPERSAGAASAATGADAPS
SSSSGGIKVEPITTTSE
>Drosophila_melanogaster_Bab2_Brachycera_Cyclorrhapha_Schizophora_
Acalyptratae_Drosophilidae
MDMTKQIVDFEIKSELIGEIDQFEASDYTMAPPEEPKMVEESPQLGHLEDQNRKYSPERE
VEPTLQDPSEVVDQMQKDTESVGEVKSPEKDVETELVKSKASPMNDQALTPPPRPLTSSE
VVGLRDPEHTELRMCLEAKKSRSLPVSPQPQPNLKLAGSALFEFGQRSSPVETKIKTNPE
TKPPRRKIVPPSGEGQQFCLRWNNYQSNLTNVFDELLQSESFVDVTLSCEGHSIKAHKMV
LSACSPYFQALFYDNPCQHPIIIMRDVSWSDLKALVEFMYKGEINVCQDQINPLLKVAET
LKIRGLAEVSAGRGEGGASALPMSAFDDEDEEEELASATAILQQDGDADPDEEMKAKRPR
LLPEGVLDLNQRQRKRSRDGSYATPSPSLQGGESEISERGSSGTPGQSQSQPLAMTTSTI
VRNPFASPNPQTLEGRNSAMNAVANQRKSPAPTATGHSNGNSGAAMHSPPGGVAVQSALP
PHMAAIVPPPPSAMHHHAQQLAAQHQLAHSHAMASALAAAAAGAGAAGAGGAGSGSGSGA
SAPTGGTGVAGSGAGAAVGSHHDDMEIKPEIAEMIREEERAKMIESGGHGGWMGAAAAAT
GAASVAADSYQYQLQSMWQKCWNTNQQNLVQQLRFRERGPLKSWRPEAMAEAIFSVLKEG
LSLSQAARKFDIPYPTFVLYANRVHNMLGPSLDGGADPRPKARGRPQRILLGMWPEELIR
SVIKAVVFRDYREIKEDMSAHQYANGQGHGTYIGGGTTTNGYHSAAAAKLAAQNAALAPP
DAGSPLSSMTETLRRQILSQQQQHQQHHQQQAHHQQQPSHHQQQSPHAQSMNMYKSPAYL
QRSEIEDQVSAAAAVAAAAAKHQQQQGERRGSENLPDLSALGLMGLPGLNVMPSRGSGGG
SGGAAPNSAASYARELSRERERDREREREREL SRQYGSQSRGSSSGSGSAKSLTASQRPG
AASPYSAAHYAKHQASAYNKRFLESLPAGIDLEAFANGLLQKSVNKSPRFEDFFPGPGQD
MSELFANPDASAAAAAAAYAPPGAIRESPLMKIKLEQQHATELPHED
>Drosophila_pseudoobscura_pseudoobscura_1_Diptera_Brachycera_
Cyclorrhapha_Schizophora_Acalyptratae_Drosophilidae
MASLEVQLDAERGSTENEAQSNAEQAQSAQRQRSGGTLSPQQGGSNRSSPTTLHADDRRG
SEERGGAPGGGGGGGGAVSNEETAGPGGSSSPVAGSPSASGSPPSTPSSQQFCLRWNNYQ
SNLTTIFDQLLQNECFVDVTLACDGRSMKAHKMVLSACSPYFQTLLAETPCQHPIVIMRD
VSWGDLKAIVEFMYRGEINVSQDQIGPLLRIAEMLKVRGLADVTHMEAATAAAAAAASEA
RDAHSHSHKEATHRDTVEQREAEELLAFMQPEKKLRGDWDYHPSSSGGTSAVSTAGGNLE
LRLSPLERQQGRNVRKRRWPSADTIFNPPASPLSSLIAAERLELEQKERERQRDCSLMTP
PPKPQLQGAGGVGGGGGGGTPRRLTPALESAIHGLDMPSPAATPGPRSSRALAPSPQQHH
QQQQQQQQHPLHMPPVPPHHSHAQHLGLSPVSSQHGVSSAGASPAGESRYPLGPTAAAM
AAAMELSGLPPPAEPRLPPPPPHHHSQGVGGGGGGVGSQGVGPSGSSLADDMEIKPGIAE
MIREEERAKMMENSQAWMGATAGSALAADSYQYQLQSMWQKCWNTNQNLMHHMRFRERGP
LKSWRPETMAEAIFSVLKEGLSLSQAARKYDIPYPTFVLYANRVHNMLGPSIDGGPDLRP
KGRGRPQRILLGIWPDEHIKGVIKTVVFRDAKDMKDDSLGGHMPPYGRHSDMTLSYPGAS
GGIPGPGAASLSALACPNGMGSGGGGVTGPGGGVGVGPGVDQHMSQETAAAVAAVAHNIR
QQMQMAAAVQHQHGEAGPPVVPPPGLFNLPPHLAGGPGPPGSRGSISPALSSGSGPRHAP

SPCGPAGLLPNLPPSMAVALHRDPAAAALLSQHQQQQHHLQQLHLQQQQQQQHHLHQQ
VAAAHHGMPHKSGFGASSMAAASAASTSASSSSSSHQHSHSHPHSHSHSHGEKQQKKGSP
HRSETPRLHSPLGDLGLEMSSYKREYSPSRLFADDLAELVGASVSSSSSVTQGAAGGGSS
AAIGEAPRSSSSGGIKVEPITTTSE
>Drosophila_pseudoobscura_pseudoobscura_2_Diptera_Brachycera_
Cyclorrhapha_Schizophora_Acalyptratae_Drosophilidae
MDMTKQIMDFEIKSESGGDCEANDYTMATATEEPKGAPEAATQNDGDTKSSCHEEEEAAT
GTQATATGPATEKAAADLDEADEEAAEPKQNLIKDPPLTPPPRPLTSSEVVGLRDPDDPE
LRLQLEAKKSRSLPVSPQPQASHKLAAAALFEFGQTSPGRGSAERTPVKAIKPDQKVAPA
RRRLPPVSGGATDNQQFCLRWNNYQSNLTNVFDELLQSESFVDVTLSCEGQSIKAHKMVL
SACSPYFQALFYDNPCQHPIIIMRDVHWSDLKALVEFMYKGEINVCQDQINPLLKVAETL
KIRGLAEVSAGASRDGASAHPMLPDQRMSVYGEDEEEDEELAAAILRQDHEDPEPKRARL
MADTALDLNQRQRKRSRDGNYATPSPLHGETLRPDVESEASPRTAVTPSGQSQPTSTIVR
NPFASPNPQSLQAHLLTGTGTSSSASSSSSGGTSSSTVGTAAVGTSSSSAASSSSAGTYR
TAARSCSPPPHHQHQHQHHHPSGSNGSSAGALLHSPTGGGSSGGSQSSLPPHMAAAVAAA
AHHAAAAVPQQAPPPAMHHHAAAAAAQQLAAQHQLAHSHAMASALAAAAAGGNGGGAAAA
PASGAVGGSVPSASSVGSSHHDDMEIKPEIAEMIREEERAKMIESGGHGGWMGAAAAATG
ASVADSYQYQLQSMWQKCWNTNQQNLVQQLRFRERGPLKSWRPEAMAEAIFSVLKEGLSL
SQAARKFDIPYPTFVLYANRVHNMLGPSLDGGTDPRPKARGRPQRILLGMWPEELIRSVI
KAVVFRDYREIKEDMGAHQYANGQAHGTYIGGGTTSNGYHSAAAAKMAAQNAALAPPDAS
SPLSTMTESLRRQILSQQQQAAQHQQHQQQAPHMYKSPAYLQRSEIEDQVSAAAAVAAAA
AKHQQSGGGGGGGGGGRGSENLPDLTALGLMGLPGLNVLPSRGAGGNGGGGGGAAAPSSA
ASYARELTRERERDRELKEAMQARQYGNQSRGGSGGSNSSAKAQSNRPGAASPYSSHYAK
QQQQQQQQQQQQHSKQQEQHPNYAYNKRFLESLPAGIDFEAIANGLLQKSVQAQNKSPRF
EDFFPGPGGPQDMSELFVNSDAAAAAAAAAAAAAYAPVRESPLMKIKLEQQHATELPHED
>Drosophila_virilis_1_Brachycera_Cyclorrhapha_Schizophora_
Acalyptratae_Drosophilidae
MASTETPTSRQSSDRSNAAATAQTDSETYATEQAQAAQRQRSGGGVGSPTSQQDKSKTVS
PPLTTPPDANANANANANANANVDVNVNVNAGSSSPGSRSTPQHQLPQQFCLRWNNYQSN
LTTIFDQLLQNESFVDVTLACDGRSIKAHKMVLSACSPYFQTLLAETPCQHPIVIMRDVS
WCDLKAIVEFMYRGEINVSQDQIGPLLRIAELLKVRGLADVTQMEEINTAAAAAAAAAAA
AAATASSTPTAQHSPKNSPAEREGSEDQLLSFMQPEKKLRSGDWDCGELRLSPLERQQSR
NVRKRRWPSADAIFQAPASPLSSLIAAERLEQEQKERERQRDCGILTPPPKQMALPLGSS
GSGSVTPRRLTPAAAALETALHGLDMPSPATTPAPAAVAAAAAAGRCQRTLAQSLPSPQQ
QQQHQQQQQQQQQHLHHLQQHQAHHGLGHAPGTSTSGASVHTAAAESRFPLGSAAAMAAA
AMELSVAAAAEPRLPPPPPHHHHSGPPTGGSAASLADDMEIKPGIAEMIREEERAKMMEN
SHAWMGATGSTLAADSYQYQLQSMWQKCWNTNQNLMHHMRFRERGPLKSWRPETMAEAIF
SVLKEGLSLSQAARKYDIPYPTFVLYANRVHNMLGPSIDGGPDLRPKGRGRPQRILLGIW
PDEHIKGVIKTVVFRDTKDLKDDSLGAHLPPYGRHSPVFPLQDMTLSYPGASGGMPVGGA
ASLGALACPNGMGSGGGGGAAVGAGGGGGDQQMSQEQMSAVAAVAHNIRQQMQMAAAAQH
QHGEAGPPPPPGALFNLPPHLAGGGGPGRASISPALSSGSNPSMGPRHAPSPCGPAGLMP
NLPPSMAVALHRDPAAAALLSQQQQHHLQQLQLHQQHQQQQQQQQQQQHALHQQQAQAAA
AAAAAAAAAAAAAHPHHMQPHKSGFGASSMPTASAGSSVAQQQQQLDKSKSKGSPLRSET
PRLHSPLAELGLELGYKREFSPTRLFAEDLAELVGVAPASTSSATTPSTGQPTAGVSGAT
TTDICSGSSSGGGGIKVEPITTSSE
>Drosophila_virilis_2_Brachycera_Cyclorrhapha_Schizophora_
Acalyptratae_Drosophilidae
MDMTKDIMDFERKLESEQYEPSDYTMVNAEIPKQPEVQPETDTERAYEMVNKTLDEAPNE
QLSSAEQQQAQARELEVKLEEEQLEKPTSVLHEQPLTPPPRPLTSSEVVGHSEPLDPELR
VQLSAKKSRSLPVSPQPLVAHNLAAIGLFEFGQTRAKMMQQEQKTKLGQKLTPVGVGVGL
GMGKSSPADNQQFCLRWNNYQSNLTNVFDELLQNESFVDVTLACEGQSIKAHKMVLSACS
PYFQALFYDNPCQHPIIIMRDVNWCDLKALVEFMYKGEINVCQDQINPLLKVAETLKIRG
LAEVSAGSSAAAASGGLGGASLLPEQRMTVYDEDEDEDELAAAAAILRASEEDQDEHVAP
KRARLLAKLRAGDSALDLNQRQRKRSRDGSYATPSPLPLRAESPQRTPSAGAGLGQSQPL
SMTTSTIVRNPFASPNPQALQQQPGCTSSASSSGSSSAASTAGNSANSCSSSNSNSGGSS
AANAYRSCSPAPPPPPPPAAHSNGSAAALSSPTGKNQGSAGAAQAQLPPHMAAAVAAAAHH

ASASASVPPPPPSASSMHHHAAAAAAQQLAAQHQLAHSHAAMASALGASLAAAAAGGAAA
PAVGGNSASSVGGHHDDMEIKPEIAEMIREEERAKMIETSGHGWMGAPATGASVAADSYQ
YQLQSMWQKCWNTNQQNLVQQLRFRERGPLKSWRPEAMAEAIFSVLKEGLSLSQAARKYD
IPYPTFVLYANRVHNMLGPSLDGGTDPRPKARGRPQRILLGMWPEELIRSVIKAVVFRDY
REIKEDMSAHQYANGQAHGAHFGSGNPTAAANGYHSAAVKMAPPDGSNPLSTMTETLRRQ
ILSQQQQHQQQQQQQQQQQQSPHVQSMNMYKSPAYLQRSEIEDQVSAAAAVAAAAKHQQS
ERRGAENLPDLSALGLMGLPGLNVLPSTQQQAGHQRGAAGGNGGGGSGGGSGAMHPSAAS
YARELSREREREREMSRDRELKEAMHARQYGNQSRGSNSSAGSKSAAGSSRPGAASPYSA
HHSHGHYAKHAKEQPGYAYNKRFLESLPAGIDFEAIANGLLQKSVNKSPRFEDFFPGQDM
SELFADAGTAAGAGSAAAAAAAAAAAAAYAPAAAAMRESPLMKIKLEQQQAAELPHED
>Themira_minor_1_Diptera_Brachycera_Cyclorrhapha_Schizophora_
Acalyptratae_Sepsidae
MSANSPSRSSPPSSTEDAATQFRVLLSQAAASTSVTTTSADSQGHGHIKRNTSGSTTPTP
TQPTTTITPTPGGGGERVSSAPSSPAAQQPHAITSTVGSAASTAPPTTASTTTTSTTTNQ
ALQRALSTTFMASVTPLSPRLSIHNLVDPGSGTSATVRSPQSPVGGACDGPPSPQPSQSS
GSNETVAPQQFCLRWNNYQSNLTCVFDQLLQSECFVDVTLACDGRSIKAHKMVLSACSPY
FQALLADTPCHHPIVIMRDVQWPELRALVEFMYKGEINVSQEQIGPLLRVAELLKVRGLA
DVNGPAASAASTATASANASHDQSYSDELKFAAVKEGGASDDDGGSLPPSPPSSLQRRST
EREPQKERTDSEELLEKKLRIGRSSEWSFGAAPSSGNAAVSAELRLSPLHQQLSRANVRK
RRWPSADTIQFNPPESPLSGLIAAERAERERERERERDKERDRDREHATPPIPPSSGEHP
HHHQHAHHLGLEMPSPSATPAPRGSSASSTSQVTVSGRTPTHSLATSPLLGHHMHRNSPH
PSSSGGGGGGGASTSSSSGGGDLSAAAAAAARFQLGSAQAAAMAAAAAAAAAHNLGGHHT
HGHPHMGHGHASTSGGLQQTQTGQQQQSPASQPQPPPPPXHASTSGGLQQSQTGQQQQQS
SASQPQPPPPPPPPPPPSAAGHHTPSIVDDLEIKPGIAEMIREEERAKMLENSHAWMGAS
GSSIAADSYQYQLQSMWQKCWNTNQNLMHHLRFRERGPLKSWRPETMAEAIFSVLKEGLS
LSQAARKYDIPYPTFVLYANRVHNMLGPSIDGGPDLRPKGRGRPQRILLGIWPDEHIKGV
IKTVVFRDAKEMKDDGSSMAHLPYGRHSELESPLTYAGMGPAAACPNGIPTGAAGAEHQM
SQEATAAAVAAVAHNLRQHMQLAAVAQQQQQQQHLQHGDGPGGGAGTGGGGYVPGPGNLF
NLPPHLQSHLVAAAAASAGAGSSGPGTGGIPLPKASISPALSSASAAAAAAAGSMLGPRH
APSPCGPGGLPMVPMHMVGGPGGRCDPTAAALLSQQQQHQLQQLHLQQQHALQQQHQQQQ
QQQQQHSLGPFGSLGPSSTSSGHHHSHHLHMQHQQQHQQQLHLQQHQQQSMTGASTSKAP
QSSSPHDERLRTGNSSSHTAPAPLHSPLTELGLDMVYKSARGFSPSRLFPDDIADLVSTI
PPPPPQTPAVHTSQPQVSATSSATATTTTSTGSMRTTGTSSAMPSSTGDTGEELPAMTSS
TAIKIEPLSTSAE
>Themira_minor_2_Diptera_Brachycera_Cyclorrhapha_Schizophora_
Acalyptratae_Sepsidae
MDIIDFTRKFVMDESQVEDLDPSSATEQHIVDASKLEASVELLTADDLSMTPAPVSSYTV
TPTASPPPAQPPQTPDEAHIEKATDLGMTEPNSCLGSDSRQSPTSNPTLCKADNNSLALA
QTSPKPQPQQDVADVPGDLECPAKVPGADMMTPPPRPFTSNEIVGLTDPVMRNVASLLGH
PKSRSLPASPNRLSVGVVEPSALAANPSLNFEAPKFAFAMDTGHGHTTISDLFASARPRR
LATPPMALGTARSLPKPRKSAKPEFAASGTPAEDSQQFCLRWNNYQTNLTNVFDELLQSE
SFVDVTLACEGQSVKAHKMVLSACSPYFQALFYENPCQHPIVIMRDVRWQELKALMEFMY
KGEINVSQDQIDPLLKVARMLKIRGLAEVSANNGAGTGTGHTLVPEQRMTVYGDDEPPES
EDDADNDDIGGLMDGPHKPKRTRLYERNPTVAIAAAAAAAAVASRSANQRKRSREGLLLH
DDTMLVDRLYGNRAYSPASPSRAAEASDNMSPPPPPPLALTASTIVRNPFASPNTQVSSH
GGVIASSSNPNSAAAPVATSSSSSSSSSSSSISTSSSSSTSAVTAVSAVSMGPSSTSASN
TPSASSGYRAQVACTPPLTAAAPTTVAAPPPPLPAQSQTQPMIPSAVSLLGSSSPDSNSN
SAGHHLSVHSHTLGGNAHHQHGHSSSSSHLHGAHGGRRCGxxxGRAAAASGGSTNSSSAGT
GSQAGSSASTLGGHHDDMEIKPEIAEMIREEERAKLIEGGHPWMGGASSSSVADSYQYQL
QSMWQKCWNTNQQNLVQQLRFRERGPLKSWRPEAMAEAIFSVLKEGLSLSQAARKYDIPY
PTFVLYANRVHNMLGPSLDGGTDPRPKARGRPQRILLGMWPEELIRSVIKAVVFRDYREI
KDEVGHSFANGQGHASPFGGGAAGHGSASAAAANGYHSSSAAKNAAAAAAAAAAAAALAP
PDSPIPLSTMTETLRRQIMSQQQQSPLGPSMNTMYKSPAYLQRSEIEDQVSAAAAAKHQQ
TQHKQQQQQQQQHHHHQQQQQAERTRPSDSLPELAMGLMGLPGLNVMANPTAAQQRGSSA
AVASYERRERERELKEAMQGRHLAQQSRGSSTLPKSSSTDSGAFPLYKQSKQDAHQQYAA
YNKRFLESLPPGIDFEAFANGLLQKSVVNKSPRFEDFFAGQDMSELFANAESAAAAAAAG

VSSYPPVRESPLMKIKLEQQATAEMPHDE
>Bactrocera_latifrons_1_Brachycera_Cyclorrhapha_Schizophora_
Acalyptratae_Tephritidae
MTSNSPPPQGGGLRPLSAADNATTSSPFRSLLSSPMLSRQRANLAARTSSTSSVSSPASP
QPPPPRPASVLPSFLGSPLTPPPPTAPTHSATCGEQGDDSLATLEHESKAGRCTPLLREE
SPIPPACSTTTHTPTTGGENIGARVISPSGTSAVGAAQAISPHSSTGSTATVPQQFCLRW
NNYQSNLTSVFDQLLQSESFVDVTLACDGRSIKAHKMILSACSPYFQMLFSDTPCQHPIV
IMRDVNWSDLKAIVEFMYRGEINVSQDQIGPLLRIAEMLKVRGLADVSAGSVSAEEAAAE
RLLKELHSPSKSCSPKRGGDGGSPASKRERKDYHDPLTESEEQLAFLDAEKKLRLGRNDW
DFSTATSGSSTSGDIIGGSSNLELRLSPLLLPQQQQQQASQQQLQQSRHVRKRRWPSADM
IFNPPPSPLSGLIAAERAERERERERERERERDRDCTIITPPQTTAAAGTAPSSTTQSSV
RSTSRLTPSLLETAAHLDLPSPSTTPAPSLLRPMRTPPGVATHLSHQQSQQQAHHQQQQQ
QQQQSHLPGQAQSQQSHLLHSHRASPASSAATPTHPSSLISGQLTATTVGSSGVSGRSEQ
GGGANDSRFALGSQAAAMAAAVAAQMDLGAMGMPHGSAPTPTHAPHHHTSSLADDLEIKP
GIAEMIREEERAKMLENSHAWMGASSSSTIAADSYQYQLQSMWQKCWNTNQSLMHHLRFR
ERGPLKSWRPETMAEAIFSVLKEGLSLSQAARKYDIPYPTFVLYANRVHNMLGPSIDGGP
DLRPKGRGRPQRILLGIWPDEHIKGVIKTVVFRDAKDLKDDSIVPHLPYGRHSPMFPFQD
GPLNYPGMSGPCPNGVPTPTGDQMSQEATAAAVAAVAHNIRQQMQLAAAVQHQHPSEGPT
GPGLFNIPPHLQPHLAAATSAGTGGAAPGVHGSVPLPKTSISPALSTTSNPSAGTMLGPR
HAPSPCGPGLSGLPPNIPPSMAMALHMGRCDPTGVLSQQQQHQLQHLHMQQQQALQQQQQ
QQQHHQQQHQQQQHGFGYGVSSMPHHGAASASATATSSSSQHYQQHQLQHQQPQAQQQQL
HQHQQHHQSASVSATATKAKCPSPSSLLEHRRSSPSGNSHLHSTLTELGLDMGYKTTRSS
AYSPTRLFSEDLAALVGASEDSPPGTTASSTNVSSTSTVTTPAMSGSHADVSITSSAASG
NISSSNIKIEPITTSSE
>Bactrocera_latifrons_2_Brachycera_Cyclorrhapha_Schizophora_
Acalyptratae_Tephritidae
MDMTKDIIDYTQKHTETADRLIAGYQSPLSEISENETNTRACAAASPFQIGSNIVEAKIT
TTINAYNEVPEQTPVNTAHAAQQLEQHKSTQANAMEPQPAAETANSMSVDKTSEATAIAA
AKTVGAIMSGDITPPPRPFTSSEVVGLSSALDDDPDLHVDVQLPKSRSLPTSPDHHRRSL
DSASAEILTRMNAALFGFEQRRGLDGAMHIPMGISKLTKQSAPLAARKSISPLGLKSKKQ
LAAAAAVTTNSQQQQFCLRWNNYQTNLTNVFDELLQNESFVDVTLACEGQSIKAHKMVLS
ACSPYFQALFYDNPCQHPIIIMRDVRWEELKAIMEFMYKGEINVSQEQINPLLKVAEMLK
IRGLAEVSAGSGHGMAAPHPMMPEQRMTIFDEEEDVESDDDTESAEDGCQKPKRARMLDS
SMSKVFPHAAALDLNLAAQRQRKRSRDGLFTDGNMSRFGANASMQPTTESAEPNKQTTNA
TTDEISGADVSGAPQQPPVAVTTSTIVRNPFASPNPNTTNNAAANANQSNSQSAIATASA
PTAASSSTPTLCSSTPTAPDTPTSLATSHSYRPPSMPSASSLKGAMSSPAAAAAAAAAAG
ATVSGMPLNVHSNPAAAAAAAAALGVNPHHPHHPHHHHHHAAAAAQQLAAQHQLHAAAHS
HAAMASALGASLAAGAGAGAAGTGAAGAANISSAVGHHDELEIKPEIAEMIREEERAKMI
ESGHPWMGAASTSSVTDSYQYQLQSMWQKCWNTNQQNLVQQLRFRERGPLKSWRPEAMAE
AIFSVLKEGLSLSQAARKYDIPYPTFVLYANRVHNMLGPSLDGGTDPRPKARGRPQRILL
GMWPEELIRSVIKAVVFRDYREIKEDLAHVYANGQMQAGSPFGAAGNAAVAAAAANGYHN
AAKLAAQNAALAPPDSPSPLSTMTETLRRQIMSQQQQSPISQTMNMYKSPAYLQRSEIED
QVSAANGKQHADARRTSDSMADLSALGGLMGIPGLNVMPAQAATRASQMHQSGAGYARER
ERERERERERELKEAMQARQYGNQSRGSIGASSGSSQKMPATATAAADSAAAAYAHYKNK
EHATNYAFNKRFAETLPPGIDFEAIANGLLQKSVNKSPRFEDFFPSEFFGNAESAAGAGA
AYPPTRESPLMKIKLEHQASAEVPHED
>Ceratitis_capitata_1_Diptera_Brachycera_Cyclorrhapha_Schizophora_
Acalyptratae_Tephritidae
MTSNSPPPQSAGIRPLAAGESATSSSPFRSLLSSPLLSRQRANIAARTSSTSSVSSPASP
QLPPPRPASVLPSFLGSPLATPPPPSQASVATGLSVDLSQEDHGIREQDPKPSPLLREES
PIPATCTSSARTPTPGGENTGTRAETPAEPSTPGGTQPISPHSSTGSTATVPQQFCLRWN
NYQSNLTSVFDQLLQTESFVDVTLACDGRSIKAHKMILSACSPYFQMLFSDTPCQHPIVI
MRDVNWADLKAIVEFMYRGEINVSQDQIGPLLRIAEMLKVRGLADVSAGSVSAEEAAAER
LLKDLHSPSKSSSPKRGGGESVSPLLKRERKDYHDALGETEDPLAYLDAEKKMRLGRTDW
NFGAATSGSSISGDNLSGSSANHELRLSPLLLPHHQQQLQQQARHVRKRRWPSADMIFNP
PPSPLSGLIAAERAERERERERERARERDRDCTIITPPQTTAASGIAPSSTTQSSARSTS

RLTPSLLDTAANLDLPSPSTTPAASMLRPMRTPPGVGVHLTHQQSQQHAHHHQQQQQQHQ
QQSQSQQSHLLHAHRSSPASSATTPTLPSSLISGQLTATPVGGSGSSAGRSERFSGAADS
RFALGSQAAAMAAAVAAQMDLGAMGMPHGTGAPPTHGPPHHHGSSLADDLEIKPGIAEMI
REEERAKMLENSHAWMGPSSSSTIAADSYQYQLQSMWQKCWNTNQSLMHHLRFRERGPLK
SWRPETMAEAIFSVLKEGLSLSQAARKYDIPYPTFVLYANRVHNMLGPSIDGGPDLRPKG
RGRPQRILLGIWPDEHIKGVIKTVVFRDAKELKDDSLVPHLPYGRHSPMFPFQDGPLNYP
GMGGPCPNGVPTPAGDQMSQEATAAAVAAVAHNIRQQMQLAAAVQHQHPSEGPGGAGLFN
IPPHLQPHLAAAAASGPAGSASGVHGSVPLPKTSISPALSTTSNPGAGAMLGPRHAPSPC
GPGLPGLPPNLPPSMAMALHMGRCDPTAVLSQQQQHQLQHLHMQQQQALQQQQHQQQQHQ
QQQQQHPLGYGVSSMSHHGGPTATATSTSSSSHLYQPQQHQHQHQQAQNQQQQQQHQQQH
QHQQQHQHQQQHQKQQHQQQHHQSVNLGGTASKMKCPSPSSLLEHQRPSPSVDSHLHSTL
TELGLDMGFKTSRSSAYSPTRLFSEDLAALVGASEDSPPDTAATSTIVSRTSTVTTSAMT
SGSHADVSITSSATSGNISSSNIKIEPITTSSE
>Ceratitis_capitata_2_Diptera_Brachycera_Cyclorrhapha_Schizophora_
Acalyptratae_Tephritidae
MDMTKDIIDYTQKHATDTAERLIASYQSPLSEISENEANTRACAAASPFHAGSNIMDAKI
NTPTAASYNERASTINNNNNTTTSGIPMGAEQCEQQTRPLETTQADAAVQQPREELNDTV
AAVEDEIKAIASQPPKTVGAAVVSGDLTPPPRPFTTSEVVGMSSALDDDPDLHVDIQMPK
SRSLPTSPDHHRHSLDSASADILTRMNAALFEFEQRRGLDTALHAPFAADMSAPMRISKL
SKSSLSAGAPLTASKPISPLGLKSKKQLAAAAAVATNTQQQQFCLRWNNYQTNLTNVFDE
LLQNESFVDVTLACEGQSIKAHKMVLSACSPYFQALFYDNPCQHPIIIMRDVRWEELKAI
MEFMYKGEINVSQEQINPLLKVAEMLKIRGLAEVSAGGGHGGMAAPHPMMPEQRMTIFDE
EDDVESDEEGAEDGCQKPKRARMLDPTLGKTFPHAAVLDLNLAAQRQRKRSREGFFTENN
MSRFGISTTMHTAGDNAETNSQVAIATNGTAADTASQPPPVAMTTSTIVRNPFASPNPNP
NHNAGAASTNQNNIQSATATPTAPSAVASSTPTPHGSATPTAPDTPTSHATAHSYRPPSM
PSANSLKGAMSSPAAAAAAAASGIPLNVHSHPVAAAAAAAALGVNPHHPHHPHHHHHHAA
AAAQQLAAQHQLHAAAHSHAAMASALGASLAAGTGPGGAGASGAPNMPPSMGHHDELEIK
PEIAEMIREEERAKMIESGHPWMGGASTSSVTADSYQYQLQSMWQKCWNTNQQNLVQQLR
FRERGPLKSWRPEAMAEAIFSVLKEGLSLSQAARKYDIPYPTFVLYANRVHNMLGPSLDG
GTDPRPKARGRPQRILLGMWPEELIRSVIKAVVFRDYREIKEDLAHIYANGQMQAGSPFS
AAGNAAVAAAAANGYHNAAKLAAQNAALAPPDSPSPLSTMTETLRRQIMSQQQQSPISQT
MNMYKSPAYLQRSEIEDQVSAANAKQHAEARRASDSMADLSALGGLMGIPGLNVMPTQAQ
AAAASRASQMHQSGGGVGASYARERERERERERERERERERERERERELKEAMQARQYGN
QSRGSIGTPSGSSGQKMPGTAAAAAAATDSAAAAAYAHYKNKEQASNYAFNKRFAEAMPP
GIDFEAIANGLLQKSVNKSPRFEDFFPSEFFGNADSAASGGASYPPTRESPLMKIKLEHQ
ASAEVPHED
>Teleopsis_dalmanni_1_Diptera_Brachycera_Cyclorrhapha_Schizophora_
Acalyptratae_Diopsidae
MASNSPLNTSPQAIAGHAADSSSPFRKLFESTTVVTKQERESSPQPAHSLHSPTNTSTVL
PSIMASFTTPECQLNTPPPIASTTDQRGEVSPSRNSPCTATTPTPVVATNNSRANTPICE
LGSPTHSVPSSTGSVSIAPQQFCLRWNNYQSNLTSVFDQLLQSESFVDVTLACDGRSIKA
HKMVLSACSPYFQTLLTDTPCQHPIVIMRDVSWTDLKAIVEFMYKGEINVSQEQIGPLLR
IAEMLKVRGLADVRGGNNYDSSETDKELNSTDIQLQQQKCTPNKTISSPQGTTSTPSPKQ
VQSRLQTAEQHDSDAYLDFVEPEKKMRLTQSDWELGSSSNNNTTGADKNLEMRLSPLHQA
AQAAARNIRKRRWPSVDTLFNPPTSPLSSLIAAERAEIERHRERERERERERERQRSLMT
PPTIPTTSCTAEATAQLSSSTRLTPSVLEAVSRLEIPSPSPTPTPNLLAPNRTPSSTLTP
SPQLLQQQGHSHHHSQTHTHQQHHHSHRTSPASSATSTTHPSSVISGGPLTPSSATSAVG
PSRGVESMHADSRFPLGPAQAAAMAAAAAAAHMDLSTAAAMGMAHPPRPPPPPPHHSTSI
VDDLEIKPGIAEMIREEERAKMLENSHAWMGASSSSIAADSYQYQLQSMWQKCWNTNQNL
MHHLRFRERGPLKSWRPETMAEAIFSVLKEGLSLSQAARKYDIPYPTFVLYANRVHNMLG
PSIDGGPDLRPKGRGRPQRILLGIWPDEHIKGVIKTVVFRDAKELKEDTLPPIPYGRHSP
IFPFPDSPLSYPGVPGPCPNGMPSAPGEQMSQEATAAAVAAVAHNLRQQMQMAAAAQHQH
SEGASGANLFNIPPHLQSHMSAAGPGPGGIPLPKASISPALSSASNHGAAGGMLGPRHAP
SPCGSLAGLPNLPPSMAAALHMVGGTSAARCDPAAMLSQQQQHQLQQLHLQQQHALHQQQ
QHQQQQHQQQQHAATHALGFGISSMSHSLTSTTATATATSSSTQKLQLQQEEQQHQQHEQ
QQQQQHQHTTSAVNTSSSSIIAKPKSVKDSPTQQRSSPRHETPLHSPFTELGLEIGYKHV

RGYSPSRLFTEDIAELMGAATATTSSITSSSSASPATSSILSSLTSATGNVATAASAPTS
SKISSSSIKIEPITTSSE
>Teleopsis_dalmanni_2_Diptera_Brachycera_Cyclorrhapha_Schizophora_
Acalyptratae_Diopsidae
MDMTKDNIDYVHQADSLNAILPKVSPVIATTTATNDISEVTFKANTATVTPTVSPVSAAA
TLSPLLPSTGGNAATTSAVLETNTAPVRPKSLTPDLMKTFGNITTAGDLTPPPRPLTSSE
VVGLTASLDDPDMRVDTPVPKSRSLPASPNHLTSLPHMAALFEFGRDYGVPATHAASLKL
PKPTREALHTEKSPSKPKTVPSSNQQFCLRWNNYQTNLTNVFDELLQNESFVDVTLACEG
QSIKAHKMVLSACSPYFQALFYDNPCQHPIIIMRDVRWPELKALMEFMYKGEINVRQDQI
NPLLKVAEMLKIRGLAEVSSGAHGVAAHPMLPEQRMTVYDDEEDDDDISDARHLLTNDDD
DDDDEEACHKPKRARILDAALEKLRAPTALELNLSAHHQRKRSRDGALLDDSILRRSRSH
TPLPRPTEKQHNAAAAENAPANDAASNTQPIAMTTSTIVRNPFASPNPTTASNNSVTTNT
SSTSSSTTSSGTSSSSATSASSAYRPTVGCSSPLPSAHGNSAQRSSPNDNGTPNSANTHS
HPAAAAAAAAAALASSHHPHHSHHHLHHPAAAAAQQLAQQQLHAAAHSHAAMSAALGASL
AAAAAGSSGGGGSGAGAGAHHEELEIKPEIAEMIREEERAKLIEGGHPWMGGGASTSSVT
DSYQYQLQSMWQKCWNTNQQNLVQQLRFRERGPLKSWRPEAMAEAIFSVLKEGLSLSQAA
RKYDIPYPTFVLYANRVHNMLGPSLDGGTDPRPKARGRPQRILLGMWPEELIRSVIKAVV
FRDYREIKDEMSHPYANGQGHVSPYGTPVNPGANGYHNAAKFAAQNAALAPPDSASPLST
MTETLRRQIMSQQQQSPISQTMNMYKSPAYLQRSEIEDQVTAVAAAAKHQAERRGSENMP
DLSALGLMGIPGLNVMPTQGQRSSQMHSSSFGRERERERERDRERERERDRERERERERD
IDRERERDRELKEAMQARQYGNQSRSSSAGSTQKQSLGPNNNTISGGGSNFQHMRNKEHP
SYAYNKRLLESLPPGIDFESIANNLLQKSVNKSPRFEDFFPGQDMSELFSNPEMAAGGSY
SSMRDTQLMKIKLEQAAEVPHED
>Musca_domestica_1_Brachycera_Cyclorrhapha_Schizophora_Calyptratae_
Muscidae
MDSKSSPTHRQQDESPAPNPTTTSSSCSSAADNVQTFRELLEAASSKAMAKQQQQPSQQQ
QQHSPQAPRSHSRSSSLLHSLTGSPKRSSSPGMEPKKAKIEQGDTQHHKTPPYSSDIGDS
AQQRVGGNLALEASYSPHSDRSSAGSHSVNGSSQQQQQFCLRWNNYQSNLTSVFDQLLQT
EAFVDVTLACDGRSIKAHKMVLSACSPYFQTLFSTTPCQHPIVIMRDVNWPELKAIVDFM
YKGEINVSQDQIGPLLRIAEMLKVRGLADVGHISTSSNEAPHSSTKADQTSTSDKELYSP
PLHLSPKIRKKSLSPFGKRPPSRRSSDQNEPADSDMELLTPMEPEKSRSRVEASGWDLPS
TSGRSGLELRLSPPMVSRNVRKRRWPSADTVFNPPESPLGSLIAAERAEQERNRERERER
QRGRDSSLFTPPLPVITGSSSSVLDASAHLEFPSPSPTPAPSLLPPARTPSSLLASSASP
HLQLSHHHQQQQQQQHHHHHLHHQQQQQQAQQHQGQQSSAAQHSSSSTSAGIGSGGGG
GLQSHRSSPASSVTSTHPSSILSRPLTPSPTSVSGPGSSRLETERFSAQAAAMLGDISAS
AAASMGMRSLPSSGPAAMTEGGRLHGSSIADDLEIKPGIAEMIREEERAKMLENSHAWMG
GSSSSIAGADSYQYQLQSMWQKCWSTNQNLMHHLRFRERGPLKSWRPETMAEAIFSVLKE
GLSLSQAARKYDIPYPTFVLYANRVHNMLGPSIDGGPDLRPKGRGRPQRILLGIWPDEHI
KGVIKTVVFREGKDMKDEGLVPHLPYGRHSPIFPFQQEPSVSYPGVSAQCPNGIPTPTPT
GDQMAQEATAAAVAAVAHNLRQQMQMAAAQQQQQQQQHGPEGPGGQAGLFNLPPHLAGS
GALPVPSGPGGMILPKTSISPALSSASSSGAVGSMMGPRHAPSPCGPGMPVMTQLPHSVA
AALHHKRQQRHHYQISGYEIYFFNQYQIYHPGSMTGGMGGGGGTPTHTHHTTATTTASST
KSSSSTVSPLRRTSPPSVSPLTELGLEMAFKPSRAFSPSRLFSDDIADIVGAAAAAAAVA
AASSTSSSTCTTTATVTTATITGSSSYSLPATSSSSMMAMDAAGQQHQSSSIASSAGISP
SHSSTSMATTTAAATVVTSASSSNISSTGIKLEPITTSSE
>Musca_domestica_2_Brachycera_Cyclorrhapha_Schizophora_Calyptratae_
Muscidae
MDFTKDILDFTLKVERGEMDLGESTKPMMPEANDFNMFSEPSNCPEQQQVEEQQAQATQS
ETNDKTNTKTTTTNETETTTATTTMKTPPEVMAKPTTPQLIESVATQESSVKPTMSGDIS
PPPRPLTSSEVVGFTNAIEDPEMQLDATVALNKSRSLPASPNHLAPTHLGELFGFAAMKS
LDAPTTKIPDTLEERLLTNLIPKVLPKAASKALPEPGRNNQQFCLRWNNYQSNLTNVFDE
LLQNESFVDVTLACEGHSIKAHKMILSACSPYFQALFYDNPCQHPIVIMRDVRWQELKAL
MEFMYKGEINVSQDQINPLLKVAEMLKIRGLAEVSSTQGTGSQLLPGHRPMYQEDEEEEE
EQGDDEGNNNDDQDASPNAKRRKIQVSQRMATAPVEEVMNMAEQRMRKRSRDGFFVQDGR
MTPKSPGNNNNNNNTRNLGDLSGPQLKDKESCGFSSPANPGSITTSTIVRNPFASPNPLG
TTTPMENTTALNASSSSSSSSCIEGAGSASGSHLNSAQTASPSIAASSNTNPVALQYRPS

GERSCSPPLMTPTHVGSRSASVGGNTATPSSLPSPSSNSSGPPPPPPISIHPHPAAAAVA
AAALANPHHPHHHAAAAAQQLAAQHQQLQAAAHSHAMASALGASLAAAAAGASTTASNPA
GASSSHHDDMEIKPEIAEMIREEERAKMIESGHPWMAGATTSSVTADSYQYQLQSMWQKC
WNTNQQNLVQQLRFRERGPLKSWRPEAMAEAIFSVLKEGLSLSQAARKYDIPYPTFVLYA
NRVHNMLGPSLDGGTDPRPKARGRPQRILLGMWPEELIRSVIKAVVFRDYREIKEEMGGL
PFANGQAHAAALFGASPGAGSAASAANGYHSAAKLAAQSAAAMGGHQDSPSPLSSMTENL
RRQILSQQQQSPINPAMNIYKAPAYVQRSEIEDPMMAAGKQHSASGAGVAGSAAGVAGDR
RGSENLPDLSALGLMGLPGINVIPTAGGPGSGSSGRSNHMHSNAAAAAFSRDLERERERE
RNREREREHRERERERERERERERERERELKEAMQARQYCNQSRGSASGASSAADMQKSA
GSISPYTHQHMKNNKDQMAVNYAYNKRFLENLPPGIDFEAIANGLLQKSSAAAAAAAAAA
ASSTAANKSPRFEDFFPGPDMSELIANAEASSASAQFPAQREYNLMKIKLEQQPTEVQHE
D
>Stomoxys_calcitrans_1_Diptera_Brachycera_Cyclorrhapha_Schizophora_
Calyptratae_Muscidae
MDSKSPLHQQQETANTTATASASPSSSSNQPAISQDSVAAYRGLLEASAKARGAKQHSPP
QGSAALLQSLALSPHRPSSREQKKASAIDDDQEHNSPAYVSELNEPQSHMRSAEDQAPAY
SPHSERSSTGSTSVQGAQQFCLRWNNYQSNLTSVFDQLLQNEAFVDVTLACDGRSIKAHK
MVLSACSPYFQTLFSTTPCQHPIVIMRDVNWRELKAIVDFMYKGEINVCQDQIGPLLRIA
EMLKVRGLADVGNIASSSETAQLSNKTEPSTSPDKEYYSPSRAPEASSPKMRKKSLSPYS
KRRSSEPQEQTDMDLLTQQETEKSRLRMEVPTWDLNLPASTSRSGLELRLSPLHQQTHTP
LVGRNVRKRRWPSADTIFNPPESPLGSLIAAERAEQERNRERERERERDRERERVRDRDG
SLFTPPMPVISGASSNILEPSTHLDFPSPSPTPTPNILPSGSRTPATLLTSVSPHLQISQ
QHHHHHLQQQQQQQQQQSQTHHSSQHSQHSSAHGGAGGSGGGSGSAGQHHISGLQSHRS
SPASSVTSTHPSSILSRPLTPSPASVTGSGGVGGSGGGVGVGVGSRLGDSDRFSLGPAQA
AAMLGAAAASQMELSASAASMGMRSLPSSGQTIGQGPSHHSSSIADDLEIKPGIAEMIRE
EERAKMMENSHAWMGGSGSSLTADSYQYQLQSMWQKCWNTNQNLMHHLRFRERGPLKSWR
PETMAEAIFSVLKEGLSLSQAARKYDIPYPTFVLYANRVHNMLGPSIDGGPDLRPKGRGR
PQRILLGIWPDEHIKGVIKTVVFREGKDLKDEGIAAHMPPYGRHSQEPSLSYPGVSAQCP
NGIPTPTPSGDQMAHEATAAAVAAVAHNIRQQMQMAAAQQQQQQQQQQQQQQQQHQSHGD
VVGAGQGLFNLPPHLAGSGALPVPSGPGGMMLPKASISPALSSTSSSGAVGSMMGPRHAP
SPCGPGLPVISQLPPGVAAALHMAGSPASRCDPSTLLSQQQQHQLQQLHLQQQHVLHQQQ
QQQQQQQQQHKHHTHHQHPHHPAQHQLSYGQHHHLSLSHPSGSATMVGPPTHSHLQHPS
STATTTASSTKSSSSTVSPLRRSSPSHSVSPLTELGLEMAFKPARPFSPSRLFADDIADI
VGAAAAAAAAATNVSSTSSSACTTTATMTTATITASSSSSTYGLPTATGAGAMDSSSSSA
LGSVAISSATDAGGGMASSAAPTTSTATVVSSAASSSSSNISSTGIKLEPITTTSD
>Stomoxys_calcitrans_2_Diptera_Brachycera_Cyclorrhapha_Schizophora_
Calyptratae_Muscidae
MDLTKDMLDFTRKLEGGEGEMEVPPEEKLMATSSNEFSMFAEQGNAEEESLLMSMQQEKN
FNETEPLIKMPLEVVQKPTTPQLIENLGAPSSSSAAKALQSGGDISPPPRPLTSSEVVGF
STALDDPEMQLDATVALNKSRSLPASPNHLNATHITDLFGFAAMKSSKITTAAPATHSNS
LETTTSMTIPRIIPNMTPKSLAAKGLPKFPASDARNSQQFCLRWNNYQSNLTNVFDELLQ
NESFVDVTLACEGHSIKAHKMILSACSPYFQALFYDNPCQHPIVIMRDVRWQELKALMEF
MYKGEINVSQDQINPLLKVAEMLKIRGLAEVSSSGGARAGGVGASLLGSHERSMGFEDDP
EDMEDEEEALVNDLEEDAEDSPTNAAKRPRIQVSQKLVSPLNMNLGEQRMRKRSRDGMFI
EDERILETSLNARNRSRSRSRSPQARDKEAAASINPVAMTTSTIVRNPFASPNPTQGSTQ
NSSGSTANAPTQASSSVNSTSATSISSAAAAAAASPNVVALPYRSLERSCSPPMMTPPQA
RAQSSVSNTTPSALPSPSSNSSAGPSGTPSISLHSHPAAAAVAAAALANPHHPHHHAAAA
AQQLAAQHQQLQAAAHSHAMASALGASLAAAAAGATPATNPAGSTSAHHDDMEIKPEIAE
MIREEERAKMIESGHPWMAGATTSSVTADSYQYQLQSMWQKCWNTNQQNLVQQLRFRERG
PLKSWRPEAMAEAIFSVLKEGLSLSQAARKYDIPYPTFVLYANRVHNMLGPSLDGGTDPR
PKARGRPQRILLGMWPEDLIRSVIKAVVFRDYREIKDEMGGLPFANGQAHAAAMFGASSA
SSATSAANGYHSAAKLAAQSAAIGHQESASPLSSMTENLRRQIMSQQQQSPLNQSMNIYK
APAYVQRSEIEDPLTAVANKQHTAATTSSASGDRRSADNLPDLSALGLMGLPGLNVIPTG
GGAGSAGAGVSSRSNNHLHSNAAAAFSRDLERERERERNRERDREHRERERERDRERERA
ERERERELKDAMQARQYGNQSRGSAGVSSDMQKPSGTSSPYAHAHMKNKDHLTNYAYNKR
FLDNLPPGIDFEAIANGLLQKTAANKSPRFEDFFPGPDMSELIANAEAAAASAPFPAQRE

YNLMKIKLEHQATEVQHED
>Lucilia_cuprina_1_Diptera_Brachycera_Cyclorrhapha_Schizophora_
Calyptratae_Calliphoridae
MDTNSPPPRAVSSQAPAATTGVREVDSPFRETQETKQNSPRPPSRPSSVLPHLLASFVSS
PTASCSKKPSPDRRDESQGGKRSPTNDTSLKSPTAHSDTSPSTPTCGMPPFSPQSDRSSC
GSTSATAPQQFCLRWNNYQSNLTSVFDQLLQNEAFVDVTLACDGRSIKAHKMVLSACSPY
FQTLFSTTPCTHPIVIMRDVNWFELKSIVDFMYKGEINVSQEQIGPLLRIAEMLKVRGLA
DVSNIGGSSAHISARAQEPPNEKELFSTQTIMDSPKRHDMITSPSTKRPPSQSQDSDNER
SENDRDISSLAETEKKSRLEPADWDITGSVKTTESSNLELRLSPLQHQHLLGRNVRKRRW
PSADAIFNPPESPLSSLIAAERAELERERERTREREKSREVTLFTPPIPVTSCSSAATSS
LNSGGMSTNVLDTSVLMDITSPSPTPTPNLLPPARTPSGMLTPSPHLQISQHQHHSHQSL
QSHRSSPASSVTSTHPSSILSRPLTPSPASVGHAGPSSSRLGEGGQMAGDSHRFPLGPAQ
AAAMAAAAAQMDLGGTSSLGMRPHSSSAGPPMSAGPGHHTSSLVDDLEIKPGIAEMIREE
ERAKMLENSHAWMGASGSSIAGADSYQYQLQSMWQKCWNTNQSLMHHLRFRERGPLKSWR
PETMAEAIFSVLKEGLSLSQAARKYDIPYPTFVLYANRVHNMLGPSIDGGPDLRPKGRGR
PQRILLGIWPDEHIKGVIKTVVFRDAKDLKEEGLAHLPYGRHSPVFPFQEGPLSYPGVGG
QCPNGMPGATGDQMSQEATAAAVAAVAHNIRQQMQMAAAAQQQQQMGESHGGPNLFNLPP
HMASGGPMSGPPGSGGVPLPKASISPALSSSSNTASMSAMMGPRHVPSPCGPNLPGMPQL
PPGMAVALHMAGSGPGGRCDPTSVLSQQQQHQLQHLHLQQQHALHQQQQKHQQQHQMAFG
PHGTSLSHTSVGGTPSHTVVHPSLQKSTTATSTKPHSSTSSPSRRNSPHSLTPLHSPLTE
LGLEMSFKPSRPFSPSRLFSDDISDIVGVAASPLRSPSTSTHPSSSLAAVTTATITTTSS
INTATVRASSSEPSGSAATSQTTTATSAGGIDSTASASSNISSTGIKLEPITTSSD
>Lucilia_cuprina_2_Diptera_Brachycera_Cyclorrhapha_Schizophora_C
alyptratae_Calliphoridae
MDLTKDIIDFTQKVDYSDNIKSSETKILSGEDFGIFSNMEKISKMAQEANALSSNEHNAE
KFEAKMPTAQLQKPTTPQLIENTAHVSSIKPAMGRDISPPPRPLTSSEVVGFITTLDDPE
MQLDPAVVLNKSRSLPASPNHLKTTHISDLFTFKPEKQREMPLKQTGFLSDKKLSNLIKP
NAEALTRNNQQFCLRWNNYQTNLTNVFDELLQNESFVDVTLACEGQSIKAHKMILSACSP
YFQALFYDNPCQHPIVIMRDVRWQELKALMEFMYKGEINVSQEQINPLLKVAEMLKIRGL
AEVSPNGNGMAAQQLIPEQRMTVFDDEEDEEEVAAKNDDTEEDKVECSKAKRARIQISAK
TTSALDLKLAEQRVRKRSRDGLLIDEERLRPRSLSRSPSPSPQNREKFAESTTPPPIVMT
ASTIVRNPFASPNPSNQPAATNASATSTSSSSSSSGSGSATSGSSVSSTPAAASYRARER
SLSPSLMTAHPGTSSLSLASPNSSSTTPQISIHPHPAATAAAAAALANPHHPHHHAAAAA
QQFAAQQQLHAAHSHAAMASALGASLAAAAAGAGSASAGPTSSAGSSSHHDDMEIKPEIA
EMIREEERAKMIESGHPWMGGASTSSVTDSYQYQLQSMWQKCWNTNQQNLVQQLRFRERG
PLKSWRPEAMAEAIFSVLKEGLSLSQAARKFDIPYPTFVLYANRVHNMLGPSLDGGSDPR
PKARGRPQRILLGMWPEDLIRSVIKAVVFRDYREIKDEMGGLPFANGQPHAIFGSSPGGS
AGVSNGFHNAAKLAAQSSGMAQESTSPLSTMTENLRRQIMSQQQQSPISQSMNMYKSPSY
VQRSEIEEQISGVSSKQHADRRGSENLPDLSALGLMGLPGLNVIPTGGSRNTQMHSNTAA
AFSRERERERDRERERERDRTRDRERERERERERERERQYSNQSRGSAAGDSQKSTGSAS
PYSQMKMKEHPNYAYNKRFLDSLPPGIDFEAIANGLLQKSTNKSPRFEDFFQGQDMNDLM
TGPESASAGAHFPPQRETNLMKIKLEHQPTELQHED
>Phormia_regina_1_Diptera_Brachycera_Cyclorrhapha_Schizophora_
Calyptratae_Calliphoridae
MDSNSPLARAETTHEQTLTAGHKEYGSPLHETQEGKASSSRPPSRPSSVLPNILSSFGSS
PTTSSHTKRQTPERKQEDILESKNSPLKSPTAHSDTCPSTPICGVPPYSPQSDRSSCGSA
SATAPQQFCLRWNNYQSNLTSVFDQLLQNESFVDVTLACDGRSIKAHKMVLSACSPYFQT
LFSTTPCQHPIVIMRDVNWFELKSIVDFMYKGEINVSQEQIGPLLRIAEMLKVRGLADVS
NLGSSANTAQLSTLQTAANEKDLYSSQTLLESPKRSENLQSPTTKRPPSQSLETDPEHCE
QVRDISSLNEVEKKSRLEASEWDISGSVKTSESSNLELRLSPLQHQHLLGRNIRKRRWPS
ADAIFNPPESPLSSLIAAERAEQERERERTREREREKAREITLFTPPIPVTSCSSGNTSS
LNTSALETTVHMDITSPSPTPTPNLLPPARTPSGMLTPSPHLQLSHHHSHQPHQSHRSSP
ASSVTSTHPSSILSRPLTPSPASVSHTGPGSRLEGSQMTGDSSHRFSMGPAQAAAMAAAA
AQMDLGAGPSMGMRPHSSSGPPMPTGPGHHTSSLVDDLEIKPGIAEMIREEERAKMLENS
HAWMGASGSSIAGADSYQYQLQSMWQKCWNTNQSLMHHLRFRERGPLKSWRPETMAEAIF
SVLKEGLSLSQAARKYDIPYPTFVLYANRVHNMLGPSIDGGPDLRPKGRGRPQRILLGIW

PDEHIKGVIKTVVFRDAKDLKDEGLAHLPYGRHSPVFPFQEGPLSYPGVAGPCPNGMPGA
AGDQMSQEATAAAVAAVAHNIRQQMQMAAAAQQQQQIGEGHGGPNLFNLPPHMASGGPMS
GPQGSAGVPLPKASISPALSSSSNTAGLSSMMGPRHVPSPCGPNLPGMPQLPPGMAVALH
MAGGGPGGRCDPSAVLSQQQQHQLQHLHLQQQHALHQQQKHQQQQHQHQMAFGPHTSSLS
HSSVAGAPSHTIVHQSMQKTTTASSIKPPSSTSSPSRRTSPHSLTPLHSPLTELGLEMSF
KPSRPFSPSRLFSDDISDIVGVAASPLRSPTTSHPSSTAAVTTATITTTSSINTATVRSS
SSEPSATSATATSSAASVAIDSMASASSNISSTGIKLEPITTTSD
>Phormia_regina_2_Diptera_Brachycera_Cyclorrhapha_Schizophora_
Calyptratae_Calliphoridae
MDLTKDILDFTQKTDYNDIKSSETKMLSGEDFGIFATTTVTEKSGNLPLETHPLTATNTI
EQNSERIEMKIPTERLQKPTTPQLLENAANVSTLKPTLGRDISPPPRPLTSSEVVGFITT
LEDPEMQLDPSVVLNKSRSLPASPNHLKSAHMADLFSFKPEKSRDINSKPSIIMDEKKMP
NIIKPSAETLTRNNQQFCLRWNNYQSNLTNVFDELLQNESFVDVTLACEGQSIKAHKMIL
SACSPYFQALFYDNPCQHPIVIMRDVRWQELKALMEFMYKGEINVSQDQINPLLKVAEML
KIRGLAEVSTTGGSMAAQQMLPEQRMTVFDDDEDDETMGKNIAININEHDDIDDPKAKRA
RIHITPKNTTALDLKLAEQRLRKRSRDGLLMDEDRLGASSLSRSPSPQIPEKYAETNNPT
TPPIVMTASTIVRNPFASPNPSNQPVTTINSGTSTTSSSSSSASGSATSASSISSSSAAV
TYRPRERSISPSLMSSHTGTGNLSLTSPTSSSTPQISIHPHPAATAAAAVALANPHHPHH
HAAAAAQQFAAQQQLHAAHSHAAMASALGASLAAAAAGAGSTSVGPTSSAGSSSHHDDME
IKPEIAEMIREEERAKMIESGHPWMGGASTSSVTDSYQYQLQSMWQKCWNTNQQNLVQQL
RFRERGPLKSWRPEAMAEAIFSVLKEGLSLSQAARKFDIPYPTFVLYANRVHNMLGPSLD
GGSDPRPKARGRPQRILLGMWPEDLIRSVIKAVVFRDYREIKDEMGGLPFANGQPHVSIF
GTSPGGSAVSNGFHNAAKLAAQGSSMGQESTSPLSTMTENLRRQIMSQQQQSPISQSMNM
YKSPSYVQRSEIEDQISGAASKQHAERRGSENLPDLSALGLMGLPGLNVIPTGASRNSQM
HSNAAAFSRDRERERERERERERERNRERERERERERERERQYSNQSRGSAAGESQKSAG
SASPYSQMKMKEHPNYAYNKRFLDSLPPGIDFEAIANGLLQKSTNKSPRFEDFFQGQDMN
DLMSGPESASAAHYPAQRGETNLMKIKLEHQHTELQHED
>Cochliomyia_hominivorax_1_Diptera_Brachycera_Cyclorrhapha_
Schizophora_Calyptratae_Calliphoridae
MDSNSPLSRAESSHEQSGSKEYTSPLRETTEAKSGSPRPPSRPSSVLPNILATFGTSPSV
SSHTKKNTPEHRQDDVLEGKSSPMAHSDTCPSTPICGIPPYSPQSDRSSCGSTSATAPQQ
FCLRWNNYQSNLTSVFDQLLQNESFVDVTLACDGRSIKAHKMVLSACSPYFQTLFSTTPC
QHPIVIMRDVNWFELKSIVDFMYKGEINVSQEQIGPLLRIAEMLKVRGLADVSNLSNTAN
TAQLSTVQTSSSEKDLYSSQNIMESPKQTENVQSPTTKRPQSQTLEMDHERCEQVKDISS
LNEPEKKSRLEVSEWDISGSVKTTESSNLELRLSPLQHQHLLGRNIRKRRWPSADAIFNP
PESPLSSLIAAERAEQERERERARERERERKAREISLFTPPIPVTSCSSGATSSLNTSALE
PTVHMDITSPSPTPTPNLLPPSRTPSGMLTPSPHLQITHHHSHQTHQSHRSSPASSVTST
HPSSILSRPLTPSPASISHTGPGGRLEGNQMSGDSSHRFPMGPAQAAAMAAAAAQMDLGA
GSSMSMRPHSSSGPPMSTGSGHHTSSLVDDLEIKPGIAEMIREEERAKMLENSHAWMGAS
GSSIAGADSYQYQLQSMWQKCWNTNQSLMHHLRFRERGPLKSWRPETMAEAIFSVLKEGL
SLSQAARKYDIPYPTFVLYANRVHNMLGPSIDGGPDLRPKGRGRPQRILLGIWPDEHIKG
VIKTVVFRDAKDLKEEGLAHLPYGRHSPVFPFQEGPLSYPGVGGPCPNGMPGAAGDQMSQ
EATAAAVAAVAHNIRQQMQMAAAAQQQQQIGESHGGSNLFNLPPHMASGGPMSGPPGAGG
VPLPKASISPALSSSSNTAGLSSMMGPRHVPSPCGPNLPGMPQLPPSMAVALHMAGGPGG
RCDPSAVLSQQQQHQFQHLHLQQQHALHQQQKHQQQQQHQMAFGPHTSSLSHSSMAGAPS
HTMIHQTMQKSASASSTKPLSSTSSPSRRNSPHSLTPLHSPLTELGLEMSFKPSRPFSPS
RLFSDDISDIVGVAASPLRSPSTSHPSTATAVTTATITTTSSINPATVRSSSSEPSVSAI
ATAVTTATSTASAAIDSTASTTSNISSTGIKLEPITTSSD
>Cochliomyia_hominivorax_2_Diptera_Brachycera_Cyclorrhapha_
Schizophora_Calyptratae_Calliphoridae
MDLTKDILDFTQKTDYSDIKSSETKMLSGTDFGLFGTSSAADKTNINTPQEMQPSTETIS
NIEQTTERIESKIPTERLQKPTTPQLLENAANVSTLKPTVGRDISPPPRPLTSSEVVGFI
GTLEDPEMQLDPSVVLNKSRSLPASPNHLKAAHMAELFSFKPDKIRDIPTKPSAVMGDKK
IPNIMKPNAETFSKNNQQFCLRWNNYQSNLTNVFDELLQNESFVDVTLACEGQSIKAHKM
ILSACSPYFQTLFYDNPCQHPIVIMRDVRWQELKALMEFMYKGEINVSQDQINPLLKVAE
MLKIRGLAEVSTTGGNMAAQQMLPEQRMTVFDDEEDDEIMDKNNSANSNELDDIDDPKAK

RARIHITPKSNAALDLKLAEQRLRKRSRDGLLMDDDRLAEPALSRSPSPQHTEKYIPSSA
PTPPSVVMTASTIVRNPFASPNPTNQPVAANNNGTSTSSSSSSSASGSAISASSVSSSSA
AVTYRPRERSLSPSLMSSHTGTGNLSLTSPTSATTPQISIHPHPSASVAAAAALANPHHP
HHHAAAAAQQFAAQQQLHAAHSHAAMASALGASLAAAAAGAGSASVGPTSSAGSSSHHDD
MEIKPEIAEMIREEERAKMIESGHPWMGGASTSSVTDSYQYQLQSMWQKCWNTNQQNLVQ
QLRFRERGPLKSWRPEAMAEAIFSVLKEGLSLSQAARKYDIPYPTFVLYANRVHNMLGPS
LDGGSDPRPKARGRPQRILLGMWPEELIRSVIKAVVFRDYREIKDEMAGLPFANGQPHAI
FGTSPGGSGVSNGFHNAAKLAAQNSGIGQESTSPLSTMTENLRRQIMSQQQQSPISQSMN
MYKSPSYVQRSELEDQISGVGSKQHGERRGSENLPDLSALGLMGLPGLNVIPTGPSRNSQ
MHSNPTAFSRERERERERERERERERERNRERERERERERERERERERQYSNQSRGSAAG
ESQKSTGSASPFSQMKMKEHPNYAYNKRFLDSLPPGIDFEAIANGLLQKSTNKSPRFEDF
FQGQDMNDLMTGPESAHATHFPAQRETNLMKIKLEHQPTELQHED
>Paykullia_maculata_1_Diptera_Brachycera_Cyclorrhapha_Schizophora_
Calyptratae_Rhinophoridae
MDNNSPPPARASTTVQSGGTGVSEADVQVKPSSPMSPSRPSSVLPHILASFVNSPLTAGN
KKPSPDRTSENPECKDSPCIETALKSPASCSDTSPSTPICELPPFSPQSDRSSCGSTSAT
APQQFCLRWNNYQSNLTSVFDQLLQNESFVDVTLACDGRSIKAHKMVLSACSPYFQTLFS
TTPCQHPIVIMRDVNWFELKSIVDFMYKGEINVSQEQIGPLLRIAEMLKVRGLAEVSNIG
ASGSTTHLSARHERSNEKEIYSSHTIMDSPKRQETTLLSPSTKRPPSHAHDADNERSEHY
RNVSSISETEKKSRSEAPDWDMTGSVKTTESSSLELRLSPLQHQHLLGRNFRKRRWPSAD
AIFNPPESPLSSLIAAERAEQERTRDRTQERERDKAREVTLFTPPIPVTSCSAGATSNLN
SGMSTSVLSSVDITSPSPTPTPNLLPPARTPLGMLTPSPNLQISHQQYSHQPHQSQRSSP
ASSVTSTHPSSILSRPLTPSPAAVGYAAASSRLEGSQITGDSHRFPLGPAQAAAMAAAAA
QMDLGTTGSMGIRPHSSSAPTMPSSSGHHTSSLVDDLEIKPGIAEMIREEERAKMLENSH
SWMGASGASIAADSYQYQLQSMWQKCWNTNQSLMHHLRFRERGPLKSWRPETMAEAIFSV
LKEGLSLSQAARKYDIPYPTFVLYANRVHNMLGPSIDGGPDLRPKGRGRPQRILLGIWPD
EHIKGVIKTVVFRDAKDLKDEGLVHMPYGRHSPVFPFQEAPLSYPGVGGQCPNGMPGAAG
DQMSQEATAAAVAAVAHNIRQQMQLAAAAQQQQQLSEGHVGPNLFNLPPHMAGGGPMAGL
PSSAGVPLPKASISPALSSSSTTGGMMGPRHVPSPCGPALPGMPQLPPGMAVALHMAGSG
PVGRCDPSAVLSQQQQHQLQQLHLQQQHAIHQQQQKQQQQQQQHQMTFGPHTSSLSHSSS
AGAPSHTIVHSSLQKPTTASITKPPSITSTSPSRRHSPHSVTPLHSPLTELGLEMSFKPS
RSFSPSRMFSDDISEIVGVAASPLRSPSTSYSSCSVTGVSTATITATSSINTTRASSLEP
SVIATSQASATAARASAIDSTATAKTSNISSTEIKLEPITTSSD
>Paykullia_maculata_2_Diptera_Brachycera_Cyclorrhapha_Schizophora_
Calyptratae_Rhinophoridae
MDLSKDIIDFTHKLDFNEVKSSEAKILAGEDYGIFENIEKVSCKSQQESTTSTTISTAEY
NVEKVDNKISTAQLQKPTTPQLLENTANVSSLKTTMARDISPPPRPLTSSEVVGFITTLE
DPEMQLDPAIVLNKSRSLPASPNHLKASHMVDLFSFKPEKPQQVRIKQTAFVGSKKSCNI
VKPTAETLTRNNQQFCLRWNNYQTNLTNVFDELLQNESFVDVTLACEGQSIKAHKMILSA
CSPYFQALFYDNPCQHPIVIMRDVRWQELKALMEFMYKGEINVSQEQINPLLKVAEMLKI
RGLAEVSTSSGCMAAQQLIPEQRMTVFDDEEDEEPASKHDRANVNEEEEHDKVQCSEAKR
PRVQISGKNTTVLDLKLAEQRLRKRSRDGMLMDENRLTARSMSRSPSPQMQEKYRESSPL
PPPPVAMTASTIVRNPFASPNPSNQPVATNNSATSLLSSSSSSGSNSAASASSLSSSSAA
LTYRPRERSLSPSLITSHAESGTLSITSPTSKSTPQISIHPHPVATAAAAAALANPHHPH
HHAAAAAQQFAAQQQLHAAHSHAAMASALGASLAAAAAGAGSASVGPSSSAGSSSHHDDM
EIKPEIAEMIREEERAKMIEGGHPWMGGASTSSVTDSYQYQLQSMWQKCWNTNQQNLVQQ
LRFRERGPLKSWRPEAMAEAIFSVLKEGLSLSQAARKFDIPYPTFVLYANRVHNMLGPSL
DGGSDPRPKARGRPQRILLGMWPEDLIRSVIKAVVFRDYREIKDEMGGMPFANGQPHVAI
FGTSPSGSSVANGFHNAAKLAAQNSGMAQESTSPLSTMTENLRRQIMSQQQQSPISQSMN
MYKSPSYVQRSEMEEQISGVAAKQHSERRGSENLPDLSALGLMGLPGLNVIPTSGSRNSQ
IHSNAAAFSRERERERERERERERNRDRDRERERERERERERQYSNLSHGSAAGESQKSA
GSASPYSQIKIKEHQKYAYNKQFLDNLPPGIDLEALANGLLQKSTNKSPRFEDFFQNQDM
TELMAGPEPISGSRFSSQRESNLMKIKLEHQPTELQQED
>Glossina_brevipalpis_1_Diptera_Brachycera_Cyclorrhapha_Schizophora_
Calyptratae_Glossinidae
MSSNNPLPTLSETEKNGKSPSISTLMDTTTDSKQPSRPSSVIPSILASLDSNSTSPPITH

IPKFESSSLRSRSEHIVEVSSVSETPSHDPNPHSSSPVCEVPSSSPHSEQSSSGSASANA
PQQFCLRWNNYQSNLTSVFDQLLQTESFVDVTLACDGHSIKAHKMVLSACSPYFQNLFFD
TPCQHPIVIMRDVGWCELKAIVDFMYKGEINVSQEQIGPLLRIAEMLKVRGLADVGNIES
STSETRPNELHEKQDPSPQNLVSPKISQNLSPMTSREQSLYQSPDNGPAEQAQSFLSFTE
PNKKLRLEKSTWEINNSSSSNHPNSSSIELRLSPLPHGSLVTRNVRKRRWPSAEALLNPP
SSPLSGLIAAERAEQEQERERQREHLLITPPIAISSSATNSATHLTSSSLATNVQIEIPS
SSLALLPPARTPSGILTPSPHLQISQHQSQLQQHHSSGHHSQRASPASSVASTQPSSVLS
GPLTPSPANITSSNSGESHHRFSMGSVQAAAMAAAAAAAHIDLSPAAAMGIGSHPSATIP
IVSGPAHHPPSIADDLEIKPGIAEMIREEERAKMLESSHAWMSSGASIAADSYQYQLQSM
WQKCWNTNQSLMHHLRFRERGPLKSWRPETMAEAIFSVLKEGLSLSQAARKYDIPYPTFV
LYANRVHNMLGPSIDGGPDLRPKGRGRPQRILLGIWPDDHIKGVIKTVVFRDAKDLKEET
FAHLSYTYHSPVFSFQESALNYGGPSSQCANGMPAPASDQISQEATAAAVAAVAHNFRQQ
MQMAAAAQHQQHSENLGAASLFNLPPHLISATGPSGTGPMVGPPGPGSIVLPKPSISPAL
STTSNPGGGSAVVGPRHAPSPCGPTLPGMHQLPPGMAVALHMVGGTGRCETATMLNQQQH
QHQIQQLHMQQQHAIHQQQQQKHHQQMIFGASSISHPPISAAATAVSASSQHSIATTISA
PQTKFSSSSISPSLERQRQSTPGNTTLRSSLTELGLDFGYSKTAQSFSPSRLFPDDLADL
VGASPSSSTSKKSSTLADTSSTITMAKSNTPSEILTSAACTTTTTTTTSNKSSSNSVKLE
PITTSTE
>Glossina_brevipalpis_2_Diptera_Brachycera_Cyclorrhapha_Schizophora_
Calyptratae_Glossinidae
MNLTKNIMDFTIRTRGTDLVSLETSDEFPATNHDTTATQNSEASVEFVAPAGVEFSRKTT
HWEREKVRNPSTPELLENVDKSIIKAATNCDLTPPPRPLTSSEVVGFTSTFEDPEINVDA
ALTLKKSRSLPASPNHLRNVRLTPLFAFASAKGFEPPKIAETYTINKEPSTVKQHFETDK
RNNQQFCLRWNNYQNNLTNVFDELLQNESFVDVTLACEGQSIKAHKVVLSACSPYFQTLF
YDNPCQHPIVIMRDVRWQELKALMEFMYKGEINVSQDQINPLLKVAEMLKIRGLAEVNST
GTAAAHPVVLEQRMSLYESDEEKPVENDEPYGDGEDKANRQKAKRPRINEAKKIRSKLDI
NFAPNRIRKRSRDGLLMDTDRFFSSSTHNDTYDYSKSATSNFAEKNTPSSSIPTVMTTST
IVRNPFASPNQTNQSSDVDVKKAFLTSSLASSHSAASSDASSSSVTLPFRSMTRSCSPSL
AAAAHTSREVLALVSPTESQTSVHSRSAGSVANALTDPNHPHHQAVAAAQQLAQHQFHTA
AQSHAVMASALGVSLAAVAAGASSSSGITNNPGKITAGPPSHHDDMEIKPEIAEMIREEE
RAKMIESGHPWTASTSSTSVTDSYQYQLQSMWQKCWNTNQQNLVQQLRFRERGPLKSWRP
EAMAEAIFSVLKEGLSLSQAARKYDIPYPTFVLYANRVHNMLGPSLDGGSDPRPKARGRP
QRILLGMWPDDLIRSVIKAVVFRDYREIKDELGGLSYVNGQPNVPPHFSNPNTIITNGMH
NAAKLAVQNTILASQESSSPLNSMTESFRRHIISQQQQQSPASQNMNLYKSPAYLQRSEM
TEQAPDLLSKQMNERRSTENLADLSKLGLMNLSGMNTLPSSGSCGNQSMHPNANAYAMER
EMECSREKERDHKLKEAIQARQFGNCSRGSATGAGDGQKQVPPSCFSTGPSSPYPYFKNK
DHAIQYAYNKKFLENLPPGIDFEAIANGLFQKSAVKSPRFEDLFSGQDANELLANNETGI
ATAFPSQRDSNLMQIKLEQQQITEMQNEG
>Glossina_morsitans_1_Brachycera_Cyclorrhapha_Schizophora_
Calyptratae_Glossinidae
MSSNNPLQTLPETGKKGKSPSISNLMDTTTDNKQPSRPSSVIPGILASLDSNSTSPPITH
VTKLESNTLRSRNEHIVEVSSVSETPSHVPTPHSSSPVCEVPSSSPHSEQSSSGSASASA
PQQFCLRWNNYQSNLTSVFDQLLQTESFVDVTLACDGHSIKAHKMVLSACSPYFQNLFFD
TPCQHPIVIMRDVGWCELKAIVDFMYKGEINVSQEQIGPLLRIAEMLKVRGLADVGNIES
STSDARPNELLEKHDPSPQNLVSPKIPQHLSPITSREQSLYQSPDSGRAEQVQSFLSFTE
PNKKLRLEKSAWDINSSSSGNHPNSSSIELRLSPLPHGSLVTRNVRKRRWPSADALLNPP
SSPLSGLIAAERAEQEQERERQREHILITPPIAITSSTTNSATHLTSSSLASNVQIEIPS
SSLTLLPPARTPSGLLTPSPHLQISQHQSQLQQHHSSGQHSQRASPASSVASTQPSSVLS
GPLTPSPANITSSNSGESHHRFSMGSVQAAAMAAAAAAAHIDLTPAAAMGIGGLPSATMP
IGSGPTHHPSSIADDLEIKPGIAEMIREEERAKMLESSHAWMSSGASIAADSYQYQLQSM
WQKCWNTNQSLMHHLRFRERGPLKSWRPETMAEAIFSVLKEGLSLSQAARKYDIPYPTFV
LYANRVHNMLGPSIDGGPDLRPKGRGRPQRILLGIWPDEHIKGVIKTVVFRDAKDLKEET
FAHLSYGRHSPVFSFQESTLNYGGPSSQCANGMPAPASDQMSQEATAAAVAAVAHNFRQQ
MQMAAAAQHQQHSENIGAASLFNLPPHLVSAAGPAGAGPIVGAPGPGSIVLPKPSISPAL
STTSNSGGGSAVVGPRHAPSPCGPTLPGMHQLPPGMAVALHMVGGTGRCETATMLNQQQH
QHQLQQLHMQQQHAIHQQQQQQQKHHQQMIFGASSISHPSISTATTAAPASSQHSISTTI

SAPQTKFSSSSISPSLERQRQSTPGNTALRSSLTDLALDFGYSKSTQSFSPSRLFPDDLA
DLVGTSPSSSTSKKPSTLADTSSTITVTKSNTPSEILTSVACTSTTTSTTSNKSSSNSVK
LEPITTSSE
>Glossina_morsitans_2_Brachycera_Cyclorrhapha_Schizophora_
Calyptratae_Glossinidae
MNLTKNIMDFTVRARGTDLVSLEATDELCAANHDSTVAQNSEAHVEFVAPAGVELSRKTA
YWEREKLRNPSTPELLENMDKSITKATTNCDLTPPPRPLTSSEVVGFTSTFEDPDINVEA
TLALKKSRSLPASPNHLANVRLTPLFAFTSRKAFEPPKIAETYAINKEPRTIKQHFETDK
RNNQQFCLRWNNYQNNLTNVFDELLQNESFVDVTLACEGQSIKAHKVVLSACSPYFQRLF
YDNPCQHPIVIMRDVRWQELKALMEFMYKGEINVSQDQINPLLKVAEMLKIRGLAEVNST
GTAAAHPMVLEQRMAVYESDEEKPVENDEPYADGEDITNRQKAKRPRINDTKKIRSKLDI
NFAPNRTRKRSRDGLLMDTDRFFSSSSHNDTYDYSKSSTGNFVEKNVPSSVPTAMTTSTI
VRNPFASPNQTNQASDVDAKKAFLTSSLASSHSAASSDASSSSVTLPFRSMTRSCSPSLA
AATHTSREILALVSPTESQTSAHSRSAGSVGNALADPNHPHHQAVAAAQHLAAQHQFHTA
AQSHAVMASALGVSLAAVAAGASSASGITNNTGKITTGPPSHHDDMEIKPEIAEMIREEE
RAKMIESGHPWTAGTSSTSVTDSYQYQLQSMWQKCWNTNQQNLVQQLRFRERGPLKSWRP
EAMAEAIFSVLKEGLSLSQAARKYDIPYPTFVLYANRVHNMLGPSLDGGSDPRPKARGRP
QRILLGMWPDDLIRSVIKAVVFRDYREIKDELGGLSYVNGQPNVPPHFSNPNTIITNGMH
NAAKLAVQNTILASQESSSPLNSMTENFRRHIISQQQQHSPVSQNMNLYKSPAYLQRSEM
SEQSPDLLSKQMSERRSAENLADLSKLGLMNLSGLNALPPSGPCPNQSIHHNANTYTIER
EMECSREKERDHKLKEAIQARQFGNCSRGSATGASDGQKQAPPSCFSTGPSSPYPYFKNK
DHAIQYAYNKKFLENLPPGIDFEAIANGLFQKSAVKSPRFEDLFSGQDGNELLPTNETGV
ATAFPSQRDSNLMQIKLEQQQITEMQNEG
>Eristalis_dimidiata_1_Brachycera_Cyclorrhapha_Aschiza_Syrphidae
MSTETPPPPASTPRSLSPYSQQHMHSVSAPGGEKRSLTPFGSTSPQPPPRPASVMPPASS
PHRVDSPSSASISNYKSPSALPLQSSSAGVPPSTPEANNNVINNPPLSPSSQRATTPIST
PPNQQFCLRWNNYQTNLTSVFDQLLQNESFVDVTLACDGQSIKAHKMILSACSPYFQALF
VDNPCQHPIIIMRDVKWPELKAIVDFMYKGEINVSQEQIGPLLRIAEMLKVRGLADVNGE
NSANNNSNNNSSGDASPGIRRNEGTTSGNAQNTNTSESRQLGDPACSAPFIEPEVAAKKP
RFGREWGELDVTPQQQQQLHLQQQQQQQQQQQMRNARKRRWPSSGESFGAPSPLGIAGVS
SLAGHQSVHENRSDLSSPLSSLTTGHTSXHRQHPLHQLRLCNNSSSRLINSNSSSKSRIR
PTNLNTAHTSAGASSVPAAAVTPSPHQQSAAPPPPTPPSSASSMQQQQQPLNQQQQQQQI
AHPSNALTPASAAAAFSALGGGPQMDTIGLSAAAAAAAMGMSHVDDLEIKPGIAEMIREE
ERAKLLENSHTWMGASTSSIADSYQYQLQSMWQKCWNTNQNLMHHLRFRERGPLKSWRPE
TMAEAIFSVLKEGLSLSQAARKYDIPYPTFVLYANRVHNMLGPSIDGGTDLRPKGRGRPQ
RILLGIWPDEHIKGVIKTVVFRDAKDLKDESIHLHYGRHSPVFPFQDNTLNYTGAPVCPN
GLPTGPVGPTVADSMSQEATAAAVAGVAHALRQQMQMAAAVQQQQQQQQQQQQQQQQHQH
HSHHAQQHQQATHSHHHPEPTTNIFNLPSHLSPHGAAGGIPLPKQNSPLSGSTGQNPLQR
HTSPSAPTMSGLPNQSVLNLASMSGLHMVPGMGSNMTSTGGASSSSTSTVHRGESLSQAS
HQSNISPAHIPYSSSSSVSHASQHATAPPSSSSNTRLHSTSPSESAHDLRMNSPEERLLN
SPLQMSLEPSVNLAVGVSGMAYKPSRGYSSPRPEHLFQEDIAELVGSASDRSSTANLENY
KDPPTNIKMEPITECRSE
>Eristalis_dimidiata_2_Brachycera_Cyclorrhapha_Aschiza_Syrphidae
MDVIKELPSFSEPIPFLKSLSPASTGDIPPSPRLDDERIEVPMPKSQSLPASPQHHLNVP
SIPPFFKEKEAINFSAKCLEPEIPKSTTPPIELTKPRTPTPTPPLQKSSPGNQSGHQQFC
LRWNNYQTNLTNVFDELLQSESFVDVTLACEGQSIKAHKMVLSACSPYFQALFYDNPCQH
PIIIMRDVRWNELKAIMEFMYKGEINVSQDQINPLLKVAEMLKIRGLAEVNTTETADDIS
ALASATLVSEEPAPRRSPPLPKKPRLHVNTEKLLEVNLSSTGGRKRSRSPSPVVTEHQKE
KAKQMQQEQHQHQLQYQQRQQLPQECPSPSMSSSVRNPFASPIPHLSSSTSNNHSSSAGS
GNGGNAGGGNGAGGSSGGSGSYPIATPPTNDALSLSALGINHVDEMEIKPEIAEMIREEE
RAKLIEGAHPWIGAPPPSSVADSYQYQLQSMWQKCWNTNQQSLAQQLRFRERGPLKSWRP
ESMAEAIFSVLKEGLSLSQAARKFDIPYPTFVLYANRVHNMLGPSLDGGSDPRPKARGRP
QRILLGMWPEELIRSVIKAVVFRDYREIKEETVQMYANGGGGGAPAVTHTSQYGMGVNAT
NGSASNGYHNKMGLPNGPLPADTSSPLSTMAETLRRQIQMSQQSPSPSLNMYKSPAFLQR
SEIEDPTSKRNSSEHHMATGKMAENLSELSALGLMGIPGLNVIPQTRGQHPITHQQQGQ
HLQPNASFSSRDLKDSMQHMSNAMSQAADAASRNQHSYNQQRNGSQTSNSNSHQKPGNGA

PPYHMNYKTKCDSSLYKYPDKRMLEGLTPGIDFEAIANGLLQKSHKVSPRFEDYFPNPDV
SELFAANSANEAAAGFPMKDHPIAKIKLEHQHHSTENNEE
>Megaselia_abdita_1_Brachycera_Cyclorrhapha_Aschiza_Phoridae
MSSESPPPPPDKSLSPKPPPPDADPNASSPPPSVDMLSDTNKQQQQHQHHQQFCLRWNNY
QTNLMSVFDQLLQNESFVDVTLACDSQSIKAHKMVLSACSPYFQALFFDNPCQHPIIIMR
DVKWEEMKSIVDFMYKGEINVSQEQIGPLLKIAEMLKIRGLAEVNNSETTIETEADDEPP
VAIKKPRIDVVKWPSKSQSPSSSSHHRLISSTTPPVRPLSHPPLLTTQPPQLPPPSTLPP
IAVPQPMDPMGLTALGLAPHIDEMEIKPGIAEMIREEERAKLLENSQAWMGTPATSIADS
YQYQLQSMWQKCWNTNQNLMHHLRFRERGPLKSWRPETMADAIFSVLKEGLSLSQAARKY
DIPYPTFVLYANRVHNMLGPSIDGGTDLRPKGRGRPQRILLGVWPDEHIKGVIKTVVFRD
AKELGKEDNIIAYGRHSPIYPFPDANLPVYPGGACPNGIPQPSPTPSLGDTMSPEATAAA
VAAVAHGLRQQMQMAAVAHHVDSPPNLFSLPHLQQKSPSQRSQPPVTTQPPSNLSSSRSQ
HTSPHDMRMQSPEDTPISSPIEMTLEPSVNLAVGVSGLPYGKPPEILFADDLSRNCTSSN
TSSTSANSGNIKLEPIGDCRGD
>Megaselia_abdita_2_Brachycera_Cyclorrhapha_Aschiza_Phoridae
MEVDERRPRSTGDLHTVCEDKPEIPITSVSLPASPNSLAPAIPPIIQQPPVIGKRKLASP
HPPEAPHEKCLPQQFCLKWNNYQTNLTNVFDDLLQNESFVDVTLACDGQSIKAHKMVLSA
CSPYFQALFYDNPCQHPIVIMRDVKWPELKAVIEFMYKGEINIDREKIAPLLRVAEMLQI
RGLAEVGGEVAAAQSSNSTASSMMKHQPTDVLENLSKKMRISESNLTVSVGGSGGGSGSG
SGRRPMRRTLGSSHSPSIENVHPEILTTTPLLPLHSHYDDEDEDDADEEENEVVRLKEKQ
VEQPQHLSKISVRNLNPSPITGGPLSSPTKSASLKPPPPAVSTPSTPSTMAQQPSTTAGV
TTTPFNLAVTPPEVINLGMGGNSGRGDVSVGGNGGTGEDFMRGGRGMSHVDDMEIKPEIA
EMIREEERAKLLESSNPWLSGGAGAPGAPPPSSSVAESYQYQLQSMWQKCWNTNQQNLIH
QLRFRERGPLKSWRPEAMAEAIFSVLREGLSLSQAARKYDIPYPTFVLYANRVHNMLGPS
LDGGVDPRPKARGRPQRILLGMWPEELIKSVIKAVVFRDYRELKDETGQHLLPNGSPSPH
FPIHEGLMNYPPRPNSNGYPPNAGKIHPPSPTSEASSAQSLANLTENLRRQMSQMQQSPG
NSPGNFFGLQQNFPRNTSEKRNSSNESPKTPIENVGEYNPLGFMGIPGLHIIPQAVKEER
HNSNTPNTPKDMFLKQFNNSHFKQHQQKLSARTGSSYPEYPAGINFEALANGMLHKSGLS
SVSPRFEDFFQNHQVVDLLNSTADLENREPPLPTALTKIKMEPTVADNRED
>Condylostylus_patibulatus_Brachycera_Empidoidea_Dolichopodidae
MSSEASQPPPPIISCKNMSPDAMSSKSNTESNIIDKTQLGGGDTTPPITQQHFKLPSPSS
QTSELSPASPFLKANTPPENLNRLKENLSILSTTTVTTTAASAVQFxxxxxxxxMKINLD
KNIDAIAESLSEANNLKRSKTPTTTIQHQTSIIPKQSSTLLDTKLTGLFNTPATSSLLTT
TATPSAAASATSSTSQQFCLRWNNYQSNLTSVFDELLQNESFVDVTLACDGQSIKAHKMI
LSACSPYFQALFFDNPCQHPIIIMRDVKYSELKAIVDFMYKGEINVSQDQIGPLLRVAEM
LKIRGLADVSNEDPSSDTPSPTPLLSENNNFNRSNNKLSDLTLNRNNKIKNNSTAQIYTK
DFSNRETTNGNKDCATKERIGTPITDREHEIRERDLLREKERDRDRDRDRDRDRDRDRDR
DRESPFLRKTDFEKFIPNNGPKIDFNPELTLKKARLSRDWDLASLEISLQQQQRHQQQQQ
QHQQQFLNxxxDVGKQLPVAHELMIEGRLSAARKRPRWPSMDNSRNLESPLGMLERANVA
VSLSERLSRQVTxxPLFEQQQIPHPPVTPQPPIPPFPIGPGGLDPVSLASIGGLGAHVDD
LEIKPGIAEMIREEERAKLMENSHAWLGASTSSIADSYQYQLQSMWQKCWNTNQNLMHHL
RFRERGPLKSWRPETMAEAIFSVLKEGLSLSQAARKYDIPYPTFVLYANRVHNMLGPSID
GGTDLRPKGRGRPQRILLGIWPDDHIKGVIKTVVFRDAKELKEESMHLQYGRHSPVFPFQ
EGPLGYPGGPCANGMPPQGSDSMSPEATAAAVAAVAHNLRQQMQMAAAAHHPDSPGSLFN
LPSHLQVNHPSVSGAGTPLPKSNSPTSINERNNSRESNNSNNNGNNNSNNSGNLNSGNTT
GLHNSNTSSQRHSSHQPILPNPNTLASLAGLPGLHMIPGLSNTQISRDSNLDSNNTRGNN
NGGNSNNNSHSISIYSNSGSGNNDNSNGNNSTKNLNQHSSDMSSHHHHQHHHQHHHHKEI
PRNDSPMELAHDLRMSSPDNSPLNSHLQIALEPSVNLSVGVSGMRYKSSRGYSSSPHSDH
HLFQDDIAELVSAGNNNNNNNNNNNNSSSTGRNCPPNFKEPSSSIKIEPMTECRSE
>Proctacanthus_coquilletti_Brachycera_Asiloidea_Asilidae
MPSETPPPKISSPGTTSASDKMRPKTPALTPSPQEGSQTPLANPQIHHSQTATASGSGSG
SGSQQFCLRWNNYQTNLTSVFDQLLQNESFVDVTLACDGQSIKAHKMVLSACSPYFQALF
FDNPCQHPIIIMRDIKWPELKAAVEFMYKGEINVSQEQIGPLLRVAEMLKIRGLADVNGE
QDATPATPAQSSFGSGSSSETATKKARVSREWELSNLDTNTPSGNRNRKRRWPSGDAAGS
DSPVGGIRTGSGTPTPENNDIASPVAGTAAATGTSQIPHPAATPNTMPSFPIPTPLESMA
LSSLGMSHADDMEIKPGIAEMIREEERAKLMENSHAWLGASTSSIADSYQYQLQSMWQKC

WNTNQNLMHHLRFRERGPLKSWRPETMAEAIFSVLKEGLSLSQAARKYDIPYPTFVLYAN
RVHNMLGPSIDGGTDLRPKGRGRPQRILLGIWPDEHIKGVIKTVVFRDAKELKDDSMHLS
YGRHSPVFPFQEGALNYPGAPCSNGLPNQPAASVAETMTPEATAAAVAAVAHGIRQQMQM
AAAAQQHHTDAASNLFNLPAHLQHTAGNPGVPLPKQNSPASGGQMNTNQQRHTSPSIPGL
PNPAALGLPGIPNMHMVPGLNSSTSQAVGASAGSVPHSSNAGNQQTTVTPAHLPLNTSTG
SAPSNTSRNESPSESAHDLRMNSPEESPLNSPLGMPLEPAVNLAVGVSGMAYKPSRGYSS
PRPEHLFQEDIADLVGNTSRNCPPNMSSFKEPPSGIKMEPMAECRSE
>Dasypogon_diadema_Brachycera_Asiloidea_Asilidae
MPSETPPPKVSSPGAMSACDKSRPKTPALVPSPQESSQTPSANPQTQQSPPAASSGSGSS
SGSQQFCLRWNNYQTNLTSVFDQLLQNESFVDVTLACDGQSIKAHKMVLSACSPYFQALF
FDNPCQHPIIIMRDIKWPELKAAVEFMYKGEINVSQEQIGPLLRVAEMLKIRGLADVNGE
QDAASSATPQTSSGSGSEVANKKARVSREWELSNADTASANRNRKRRWPSGDAAGADSPL
GGVGASGGTSTPDNEVASPVAGAAAAATAATGTSQMPHPAATPNTMPSFPIPTPLESMGL
SSLGMSHADDMEIKPGIAEMIREEERAKLMENSHAWLGASTSSIADSYQYQLQSMWQKCW
NTNQNLMHHLRFRERGPLKSWRPETMAEAIFSVLKEGLSLSQAARKYDIPYPTFVLYANR
VHNMLGPSIDGGTDLRPKGRGRPQRILLGIWPDEHIKGVIKTVVFRDAKELKDDSMHLSY
GRHSPVFPFQEGAMNYPGAPCANGLPSQPAASVAESMSPEATAAAVAAVAHGIRQQMQMA
AAAQHHTDAASNLFNLPAHLQHSNTGGVPLPKQNSPASSGQLNTNQQRHTSPSIQGLPNP
AALGLSGIPNLHMVPGLNSSTSQTAAVSASSVSHSSNTSNPQTTVTAAHMTLNTSTASAP
SNTSRNESPSDSAHDLRMNSPEESPLNSPLGMPLEPAVNLAVGVSGMAYKPSRGYSSPRP
EHLFQEDIADLVGNTSRNCPPNMNSFKEPPSGIKIEPMAECRSE
>Holcocephala_fusca_Brachycera_Asiloidea_Asilidae
MXXQQQQQQQQQQQQQQQQQQQSINSTINSSSSLINTNNNGGCTNSGNGGTTASSNSQSS
TSQQFCLRWNNYQTNLTSVFDQLLQNESFVDVTLACDGKSIKAHKMVLSACSPYFQALFF
DNPCQHPIIIMRDIKWPELKAAVEFMYKGEINVSQDQIGPLLRVAEMLKIRGLADVNGDQ
DGTPxxxxSNKKPRISREWELNNIEASINRNRKRRWPCSDISGAESPLSGIGTSGGTPTP
DNNDVASPVAASAGSIATGSAQLSHSGGVNPNAMPPFTIPTPLESMGLSLGMSHADDMEI
KPGIAEMIREEERAKLMENSHAWLGASTSSIADSYQYQLQSMWQKCWNTNQNLMHHLRFR
ERGPLKSWRPETMAEAIFSVLKEGLSLSQAARKYDIPYPTFVLYANRVHNMLGPSIDGGT
DLRPKGRGRPQRILLGIWPDEHIKGVIKTVVFRDAKELKDEGMHLSYGRHSPVFPFQEGT
LNYSGAACANGLPSQSAGSVAESMSPEAAVAAVAHGIRMQMAAAVQHHTDSASNLFNLPA
HLQHTTNTGVPLPKQNSPASGGQINPNQQRHASPSISCLPNPAALGLTGLSNLHMVSGLN
SSTSQTAAASAGSVSYSSSTSNPQPAVTPAHISLNASTVSVPSNISRNESPSESAHDLRM
NSPDESPLNSPLGMALEPAVNLAVGVSGMAYKPSRGYSSPRPEHLFQEDIADLVGNTTRN
CPPSMNSFKEPPSGIKIEPMAECRSE
>Hermetia_illucens_Brachycera_Stratiomyomorpha_Stratiomyidae
MSTETPPPPPKSLSPVAAAPAASEKAPRSKTPSSSPATPQDMSSRQSPTNLPSGSGSAGS
SQQREFPSPETAAARSTPTASSQPGSQQFCLRWNNYQTNLTSVFDQLLQNETFVDVTLAC
DGQSIKAHKMVLSACSPYFQALFFDNPCQHPIIIMRDVKWPELKAIVEFMYKGEINVSQD
QIGPLLRVAEMLKIRGLADVNGDPDGNSDGKAKTVSSSTAGTPEVAVKKARVAPREWELS
NVDGSGGNARTRKRRWPSSDNCNASTGSPLSTGLGSACENAPTPDPHLEAASPVATAANS
SQGPNHPQTPTPNAMPSFPIPTPLETMGLSSMGMSHADDMEIKPGIAEMIREEERAKLME
NSHAWLGASTSSIADSYQYQLQSMWQKCWNTNQNLMHHLRFRERGPLKSWRPETMAEAIF
SVLKEGLSLSQAARKYDIPYPTFVLYANRVHNMLGPSIDGGTDLRPKGRGRPQRILLGIW
PDDHIKGVIKTVVFRDAKDLKEDGMHLSYGRHSPVFPFQEGQMNYPGAACANGLPQQQAS
SNAETMTPEATAAAVAAVAHGIRQQMQMVAAAQHHPEAQAAGLFNLPPHMHTNNGGVPIP
KQTSPASSSQRHTSPSIPGLSNTSAMGMSGIPGIHIMPGLGSNPSASTNSSYTSNASSQQ
APPTLSATSAPSGSTNISRNNSPTEAAHDLRMNSPDESPLNSPLELALEPAVNLAVGVSG
MAYKPSRGYSSPRPEHLFQEDITDLVGRTSRTSSMTNFKEPSGSIKVEPIAECRSE
>Mayetiola_destructor_1a_Nematocera_Bibionomorpha_Cecidomyiidae_Ceci
domyiinae
MSKTTSIVPSTSPPSHSTSRHHQSPLTKSHASDLSNNRKVDSLSSTLPDSCDVFSGYFQD
RLNDKAVNLEKLTTTDHCCDSSDFHQQQQQQQQQPQHILANKSADGYQDDGQFSTMTTP
TIDIDAEENLSDKITATNAHHQQFCLRWNNYGRNLTNVFDQLLQNESFVDVTLACDGQSI
KAHKMVLSACSPYFQSLFFDNPCQHPIIIMRDVKWIELKAIVEFMYKGEIKVSQTEIGPL
LRVAEMLKIRGLTDVNGDHETTNICTKQRRSISPMAMQSQHQQHHQRLQSETPPLKKHRS

QKRLSSEWDDNRSSHSPPSTGLDKQHRRHKRSPLTFDNSRMADSPRIRETLTPDVATANN
FTLSSNTQSTSGATKTTRNNASPSSSSGRHTSGMNTPIFPQTSIDPITLSNLSNNDDLEI
KPGIAEMIREEERAKILENSQGWPGVPTTNSLAESYQYQLQSMWQKCWNTNQNLVHHLR
FRERGPLKSWRPETMAEAIFSVLKDGLSLSQAARKYDIPYPTFVLYANRVHNMLGPSMES
SLDLRPKGRGRPQRILLGIWPDDHIKGVIKAVVFRDAKDLKDDPSIHLAAAAAAAAAAAA
YGGHSPVYPFHDGAIPGFPSSAACSNGLLSANQSAGETISSEAAVAMAAVMGLRQQHLNA
SAINQHHSDTDLTNFRSATDSGGVGCLKQPKLGSPATSSQSNNNEINFPMPRLASPAIPS
IHSISGLSSLHLMQQHNLSTGDLIANRSRNQCNDARSTPPHTEISKFTGHIDTLMIRNDK
NQRHHINAIEQQLKHLSNKNLNNSNTTNNNNTKSNNNNNNNNNNNTKDTPDATQGSLGDK
QINFPIDDFDSVGMVDIAINGLPFLSSSAAANEEPPHLFNDELNDFDDSETGSNANTRDK
IKLEPLTECRGQ
>Mayetiola_destructor_1b_Nematocera_Bibionomorpha_Cecidomyiidae_Ceci
domyiinae
MPDSELPTDLRSSPRASITPPTPSQSQPLASTSTSTTIQTAIRQQHQLSSSALQFDMDRG
SITSSPSITTTPPKDDLHQHQHHHHAMLTNRSTPSPHTPPLSKLDTNTKMQPQHQSINDR
NSVGPTLASASASATSSMSSAIFGAGASSSSSNAQQFCLRWNNYQTNLTNVFDQLLQNES
FVDVTLACDGNSIKAHKMVLSACSPYFQSLFFDNPCQHPIVIMRDVKWPELTAVVEFMYK
GEINVRQEQIAPLLRVAEMLKIRGLADVKSDLDLNSESENLPQASPQSIFSDQQQQQQQL
HQQINNAEQQQQQQQHQQLNKKPRITARTEWQINNLDLGIESIGSGTQNKRDSNSNIQQL
QQNAGHSILTSVSASARHFANRKKRCPSAEIKRNASASPLSLIEQRNDLNASPAQQSPSS
HTSATIPSFLSAANSIDAITMASLLPNASSDDLEIKPGIAEMIREEERAKLLQNSQAWLN
AAPSTTLNSMSESYQYQLQSMWQKCWNTNQNLVHHLRFRERGPLKSWRPETMADAIMSVL
KDGLSLSQAARKYDIPYPTFVLYANRVHNMLGPSIDGGSDLRPKGRGRPQRILLGVWPED
HIKGVVKTVVFRDDKELREDNGIPMPEFDGNNPMFPFPNPQIDFSATGQSNNGALGLQLN
ELDTDQISPINISRNNLNWSSLVNASGSRSTSSRCSS
>Mayetiola_destructor_1c_Nematocera_Bibionomorpha_Cecidomyiidae_Ceci
domyiinae
MPDSPQPISQHSTSPKTPASSPAIGDVKPLPSSKTASPTASPSLPNCLQTNYKPQTSSTP
LTHEKSPANPQQYCLRWNNYTSNLTNVFDQLLQNESFVDVTLACDGKSIKAHKIVLSACS
PYFQSLFFENPSQHPIVILRDVKWTELKSIVEFMYKGEINLAHDEIGPLLNVAEMLKIRG
LADVKGEHDAIKLEHSSLASASASALGTASVSTLASATSLPDIISGKQQKMQNNANDWDL
NKIDFLMRSASASPSTAAAAVVAQSLTTQMRNQRKRNWSSNGAGSAAIPLTTSSTLSSNS
IGSNNSIVANSTISNSTTLVNNNNNNNINNNNNNNNNNNNQSSNRLNKCLTNNNNLNHNQ
ISNLDRNSNVNNMSTPSPAPLLNIDTLDARSQSSLSLSEDLPIDASNTPTPPTIRNAGLN
ISDTNNLSSLSGITLPHTIDSHSNDEMEIKPGIAEMIREEERAKLMESHPWLAPSTSASL
AESYQYQLQSMWQKCWNTNQNLIHNLRFRERGPLKSWRPETMADAIMSVLKDGLSLSQAA
RKYDIPYPTFVLYANRVHNMLGPSMDGGSDLRPKGRGRPQRILLGVWPEETIKGVVKTVV
FRDAKELRDETGFPGRHSPWYPFGDGQIDFSAGLPNHGINTNLPINLDSSDSLSLSSLVN
SGCWPSLTNQLANQKAPSG
>Coboldia_fuscipes_1a_Nematocera_Psychodomorpha_Scatopsidae
MYESQMSMVHNKTPPPQNLSTTDTKPSSPYHNNNNSNQNANMMGGNQQSIASSSSPTTSS
SMHHQQTHQHQQQQQQQQQQHQLNSGPQQFCLRWNNYQTNLTNVFDQLLQNESFVDVTLA
CDGNSLKAHKMVLSACSPYFQGLLYENPCQHPIIIMKDVKWSEMKAIVEFMYKGEINVRQ
DQIGPLLKVADMLKIRGLTDVNADNEFQRSSSSPLEHNVSVVTPPELLMNKKPKINVKDW
GEMSSLSTTVGISASLSHNRKRRHPSNGNSSPTSYDTSPSIQHESIETSSPSPLIGNRLA
SLIPSVVATPTPPSSLQTPAFSIPLPMDPLGLTALHNMSQGSDDLEIKPGIAEMIREEER
AKLLESSQNWLGASTSTINDSYQMQLQMWQKCWNRNQNLAQIPFRERGPLKSWRPQAMAD
AIFSVLKDGLSLSQAARKYDIPYPTFVLYANRVHNMLGPSIECEPDLRPKGRGRPQRILL
GLWPDEHIKGVIKTVVFRDGLDIKEEIMHYGYDGHHPMFPFHESALKRPLRPQSNGILEA
SGSLSISSSTNSIQISPNESSTSPSVQELRHQLQQQSHSPSENQPRG
>Coboldia_fuscipes_1b_Nematocera_Psychodomorpha_Scatopsidae
MSEASLPASPQHSPRTVATTPVPTIEERTSPMLNSTITANLMGSPKGSGGQSTLSPSSSS
AGCIATSVSGPALVGASTPELLHKQIQSIPSTSSQATIAQQFCLRWNNYQSNLTNVFDQL
LQNESFVDVTLACDGNSLKAHKMVLSACSPYFQGLLFDNPCQHPIIIMKDVKWPELKAIV
EFMYKGEINVRQDQIGPLLKVADMLKIRGLADVGGDNDELDTMPSTSIEESSVRKPKISV
KDFDVSPLDIRNISHSAKARKRRYQQSENGSAGGSPRQYVSTPTPTPSEILSSGDGQQHQ

QLQPTNLSATGKSRSSATPTSASTVSSFGIGNHTDPLGLSTATVSGIQQQMNSQSSGGED
LDIKPGIAEMIREEERAKLLENTHAWLGASTSSIADSYQLQLQSMWQKCWNRNQNISQHM
QFRERGPLKSWRPETMAEAILSVLKDGLSLSQAARKYDIPYPTFVLYANRVHNMLGPSID
GGMDLRPKGRGRPQRILLGIWPEEHIKGVVRTVVFRDGVEIKDEVSYDRLTDLPGFQFPE
TQLGYPVEGPSSAMVETLSAELAAAQRHADPTDLAGFEKYVKMLCNGQQQKLMKSI
>Phlebotomus_papatasi_Nematocera_Psychodomorpha_Psychodidae
MPSETPPPKIPSPSASVSVLEQQQRPKTPSSVPLVSSTPSSSSQLSHLNPNCQQGQQTGT
TTATQQEQQSFSSLPTTSQNHSEPQQFCLKWNSYQSNLTSEFDQLLQNESFVDVTLACDG
HSIKAHKMVLSACSPYFQALFFDNPCQHPIIIMKEVKWPELKAVVEYMYKGEINVSQDQI
APLLRVAQMLKIRGLADVNGDQDVNTSASSGASGSANGGSQSGSGAGGGKTDSINVKKPR
LARDFDVHERSSNNGAGGASSSSSGGQSSRDGGENLTTTARSGRKRRWLTGENQTGAGSP
VDTSTPDNLEPPSPIPIQNHPSSSITHPPTSTPTPSMPFPVPPHLDTMGLSSLALNNPDD
MEIKPGIAEMIREEERAKLLENSHAWLGASTSSIADSYQYQLQSMWQKCWNTNQNIIHHL
RFRERGPLKSWRPETMAEAIFSVLKEGLSLSQAARKYDIPYPTFVLYANRVHNMLGPSID
GGTDLRPKGRGRPQRILLGIWPDDHIRGVIKTVVFRDAKELKDESIPMGYGRHSPIFPFQ
EGALNYSGSPCTNGLGPQAPPVTGPESMSPDAAAAAAVAAVAHGIRQQIQMAAAAQHHPD
AANLVAAGGFNLPAHLNIHPNNGGSASSIPMPKLGSPATPGQLSSGNGSGAIPMPRHGSP
ALAGGAGHGLAALTGLHGLPGLPSVSVSTTRETVTSSGPASSGTGSQGHMSSPLGGGGLN
ITRLASPGSAHDLRMASPDDNSLNSSLEMALEPAVNLAVGVSGMSYKSRGYTSPRQDNLF
QEDISDIVGAPRSCPSRLSGGFKESGGVIKLEPVAECRSE
>Lutzomyia_longipalpis_Nematocera_Psychodomorpha_Psychodoidae
MPSETPPPKIPSPSASVSVLEQQQRPKTPSSVPPVSSTPSSSSQLSHLNPNCQQGQQSGG
TTATLANQEQQSFSSLPTTSQNHSEPQQFCLKWNSYQSNLTSEFDQLLQNESFVDVTLAC
DGHSIKAHKMVLSACSPYFQALFFDNPCQHPIIIMKEVKWPELKAVVEYMYKGEINVSQD
QIAPLLRVAQMLKIRGLADVNGDQDVQASSNTPGSANGGHQSASGAGGGGTATKSDTTNI
KKPRLARDFDVHERSSNGAGGASLTSSGGQNRDGGDNLAGTSRSGRKRRCLTGENQPGAG
SPVDTSTPDNLEPPSPIPIQNHPSSSITHPPTSTPTPSMPFPVPPHLDTMGLSSLALNNP
DDMEIKPGIAEMIREEERAKLLENSHAWLGASTSSIADSYQYQLQSMWQKCWNTNQNIIH
HLRFRERGPLKSWRPETMAEAIFSVLKEGLSLSQAARKYDIPYPTFVLYANRVHNMLGPS
IDGGTDLRPKGRGRPQRILLGIWPDDHIRGVIKTVVFRDAKELKDDSIPMGYGRHTPVLP
FQEGALNYSGSPCTNGLGPQAPPGAGPESMSPDAAAAAAVAAVAHGIRQQIQMAAAAQHH
PDAANLVAAGGFNLPAHLNIHPNNGGSASSIPMPKLGSPATPGQMNSGNGSSAIPMPRHG
SPALGGGSGHALAGLSGLHGLPGLPSVSVSTTRDTVTSTGPASSGTSSQGHMSSPLGGGG
LNITRLASPGSAHDLRMASPDDNSLNSSLEMALEPAVNLAVGVSGMAYKSRGYTSPRPEN
LFQEDISEIVGAPRSCPSRLSGFKESSGGIKLEPVAECRSE
>Anopheles_gambiae_Nematocera_Culicomorpha_Culicidae
MPSDTPPPSATSVSHPSPASSHHDPNDPNAPPRDPVDRSGTGTPGPSDHPTGGHLGHHQP
PSSSSSSSSSSSSSTSSSLSSLSLKRSLEEPLTTAKPSPPCSPLTMDHHHQHKAARQSR
AASPAGRSTQQQASPSAPGTGGSSSGGGGGGQQFCLRWNNYQTNLTSVFDQLLQSESFVD
VTLACDGQSMKAHKMVLSACSPYFQTLFFDNPCQHPIVIMRDVSWAELKAIVEFMYKGEI
NVSQDQIGPLLKVAEMLKIRGLADVSGDAGEPTGSRAEREAAGSRGPEELDREEHGKLLN
PLAIVGSSLLANGAASAAMAGGNGSNSTATSGSAAVQAAAAAAAAKKQRAGRDRDTTKEH
RMDARLSEFARDLSRADPHISSRDISSVAAAAAAAAAAAAGLAVGEWPLGAAGLEAAAAA
AVQASTPKSARKRRWPSGERSSIGSPADSTPDQLEVPSPIPPTPSSLAQSSGGGGGGGGG
GGGGTGSGGGGGGGSSNPLASFPLPPALDTAAMAMSSLSSSIANHPDDMEIKPGIAEMIRE
EERAKLLENSQAWLGASTSSIADSYQYQLQSMWQKCWNSQNLIHHLRFRERGPLKSWRPE
TMAEAIFSVLKEGLSLSQAARKYDIPYPTFVLYANRVHNMLGPSIDGGTDLRPKGRGRPQ
RILLGIWPDDHIKGVIKSVVFRDAKDMKEEPMMYGRHSPFPFQDNPLSYGPTAPNGQLPS
VATGTNVPDGMSQDALTAATVAAVRQQMCNMVAAAQHHPDAANLVAAAGFNLPSHCGTPP
NLSMHPAAAAAAAAAAAASNASAAGGPSGGGGGGGSSGAIPLPKMGSPAVPSTGHGNNNG
GSGAGIQMPRLGSPAGSSGLAKEHELQHHGGGGGGGGGLGGGSGGGMSRATPPGARDRAM
TARSNLAAGETGRSSSSAGSIHRSSPSSSAGSSLNHQHPAHLSHPHHQQQHHHQHHHQPH
HGHAHHLPHHNPLSHLVGSGGASGALSITKLGSPGSAHDLRISNSPDESPLASPIGLAME
PAVNLALGAGGTQPGPEDVRLHVPPPYGSKPPSRGGGAPSTGYTSNSSPPRPEHLFQDQD
IAALVATTRAACPPSRVPDYKDTAVRPTASIKVEPLTECRGD
>Aedes_aegypti_Nematocera_Culicomorpha_Culicidae

MPSDTPPPMHSSPIMDTSDRRPASVPSSSTTGLSGLSGFKREKFEPSLKPTLTPNQQFCL
RWNNYQTNLTSVFDQLLQNESFVDVTLACDGKSIKAHKMVLSACSPYFQTLFFENPCQHP
IIIMRDVKWPELKAIVDFMYKGEINVSQDQIGPLLKIAEMLKIRGLADVNGDQELNQVEH
GEPSVNIADETGSNNDSNNSSTSSLLHRGIIKKEGRLISMDPTHNNNNNPPHMQSKKPRT
SRERESSQISIRDFPRDLSRDMSRELNAAAAAAAAAAAGEWPLSAALDTVQASTPKNNRK
RRWPSGERSSVGSPADSTPDQHEVPSPIPPTPSSIVQPASTPTPQMPQFPIPPALSEQMA
GLSSLSGLAGAGSSTGAGNHPDDMEIKPGIAEMIREEERAKMLENSQAWLGASTSSIADS
YQYQLQSMWQKCWNSQNLIHHLRFRERGPLKSWRPETMAEAIFSVLKEGLSLSQAARKYD
IPYPTFVLYANRVHNMLGPSIDGGTDLRPKGRGRPQRILLGIWPDEHIKGVIKSVVFRDA
KDMKEEQIMYGRHSPFTFQADNPLNYAPNANGQMPTAGAEGLTPDALTAATVAAVRQQMC
NMVAAAQHHPEAAANLVGFNLPQHHTNLSIHPSGNAGGSSGGIPVPKVGSPAVTHQTHSN
NGGSGGIQMPRLGSPAGSSIHSGKEHDLHGPPGLNQSSHPSTSSIGGNANSNATAISNRS
ESSRSSSGSNHSSNANHHHPLAHMNSGSLSITRLGSPASAHDLRMTTPPTEESPLASPMG
LALEPAVNLAIGGGNATSGGQTSEHLGLQQYGGGSKSSSRAGYSSSSSPPRPEHLFQDQD
IAELVANTRAAAGANKDLGRPVASAIKVEPMTECRGD
>Daphnia_pulex_Crustacean
MASTSSSSSSAASGGSSPHLCLRWNNYQSNLTSVFDQLLQNETFVDVTLAADGHAIKAHR
MVLSACSPYFQHLFFDNPCQHPIVILKDTRWPELKAIVEYMYRGEISVAQEELTSLLRVA
ETLKIRGLSELNSDRHAAAAAAVTADDRSESSPLIPIHPTAGPSTSSSSSSAAAVRRQTD
SPTWPTLPHHHHQIHHHQQQQQPAWPGHVMDEEMATTPTPSPTPAPASRSSGVKRRRISG
SESPIGGASSPAAASTPGMHHQHHEEASLDHHDGPLSLVGHQQQAPPPPPPPPPHSPVAS
SASLAPQALTQAPPPPSSHHQHPLAHSDDLDIKPGIAELIREEERARMLEGTHPWLTASP
SVLSDTYQYQLQALWQKAWATGQGVMQNLRFRERGPFKTWRPETMAEAIMAVLREGLSLS
QAARKFDIPYPTFVLYANRVNNMLGPSADGGPELRPKGRGRPQRILTGSWPEDHVHGVIR
AVVFRDPSSLPRHIDREDASKLTANRSAASSSMSRLLTVHPAHQQTIQGELGGGSGSGMR
GLIHSAMNNGNSHNYNHEFALPSSSNMTGTFTHGGSLLAGGSAYPSMRSTAGPSSGVSNA
ANGLMADLMAGATNGSNNNNNNNNNMQGGSGSSTSCLSGSGLTSSSLFNALQQEVAEETR
LAALNAMTQRLMTSLEGTSRFHHAGRCLTSGRDHPGDNFGLGVSCNQQRAASGIQNPAAA
AAAAAALEAAAWFGPSSSFSGQLPIVDLAADDVTVAEDDEGDDDDEDAVQHLASFSAHHN
PTVTNPVHSKQQEQLFGE

Bab1 sequence conservation among cyclorrhaphans (Part1)

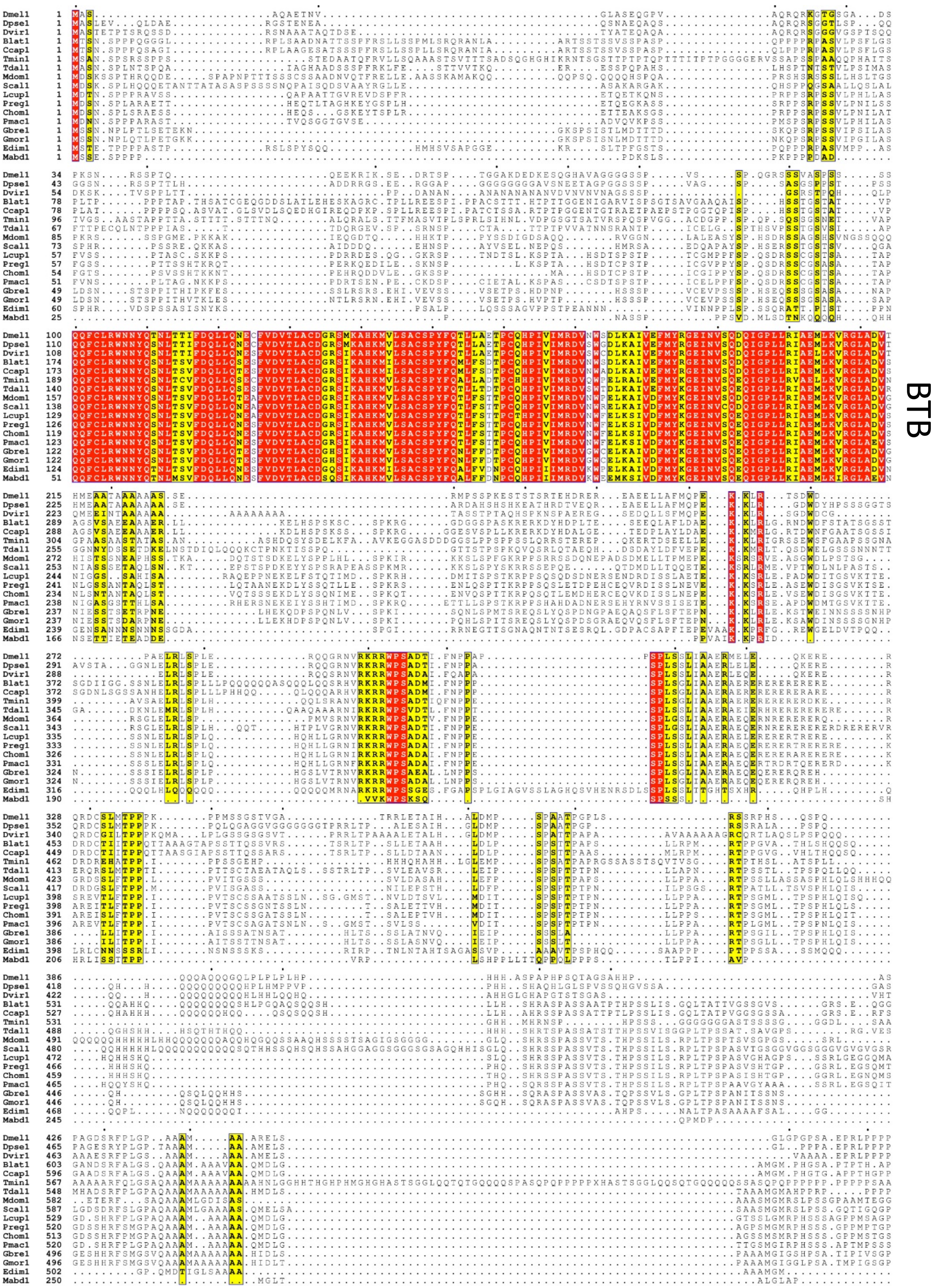

Bab1 sequence conservation among cyclorrhaphans (Part2)

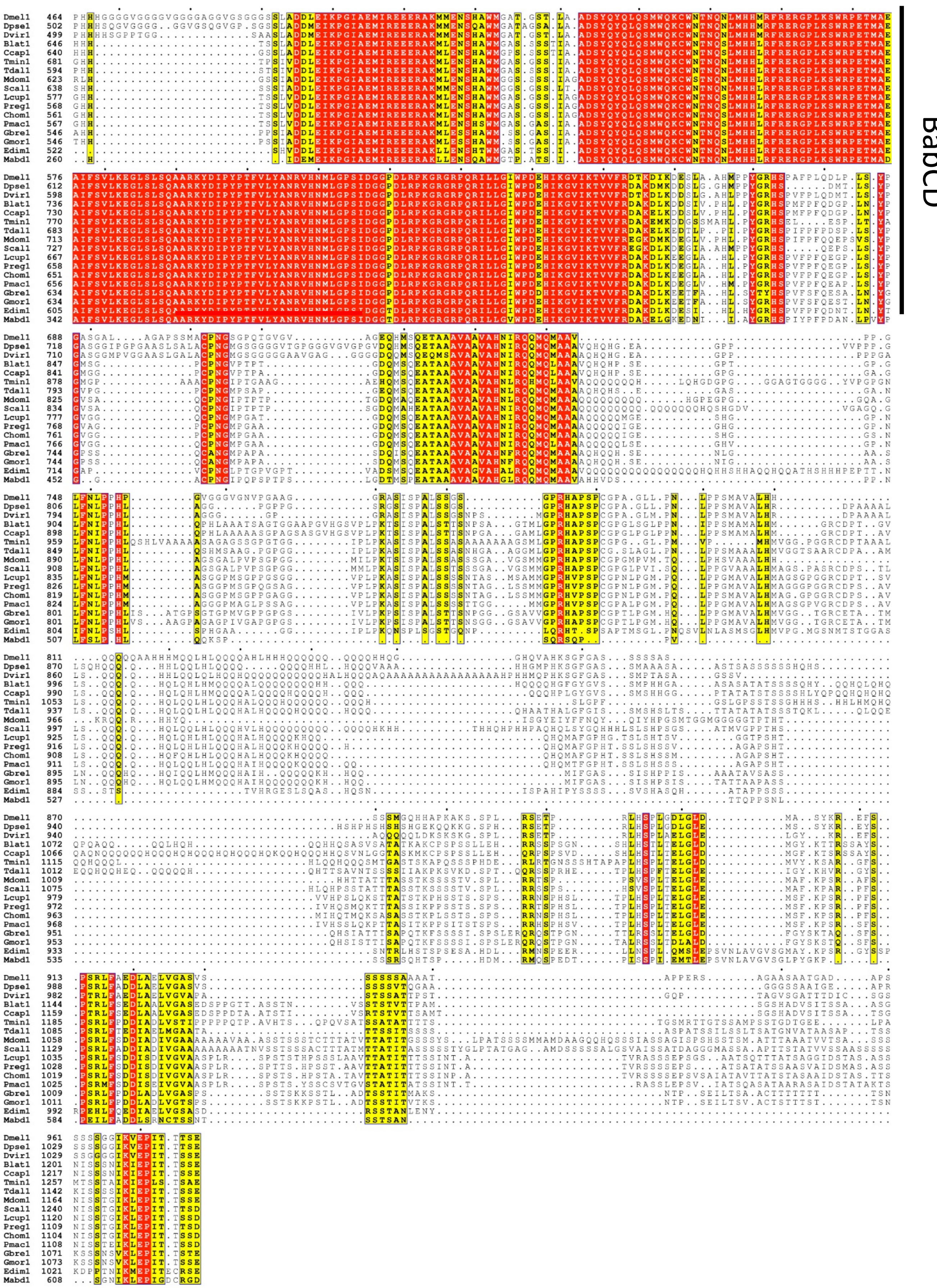

Bab2 sequence conservation among cyclorrhaphans (Part1)

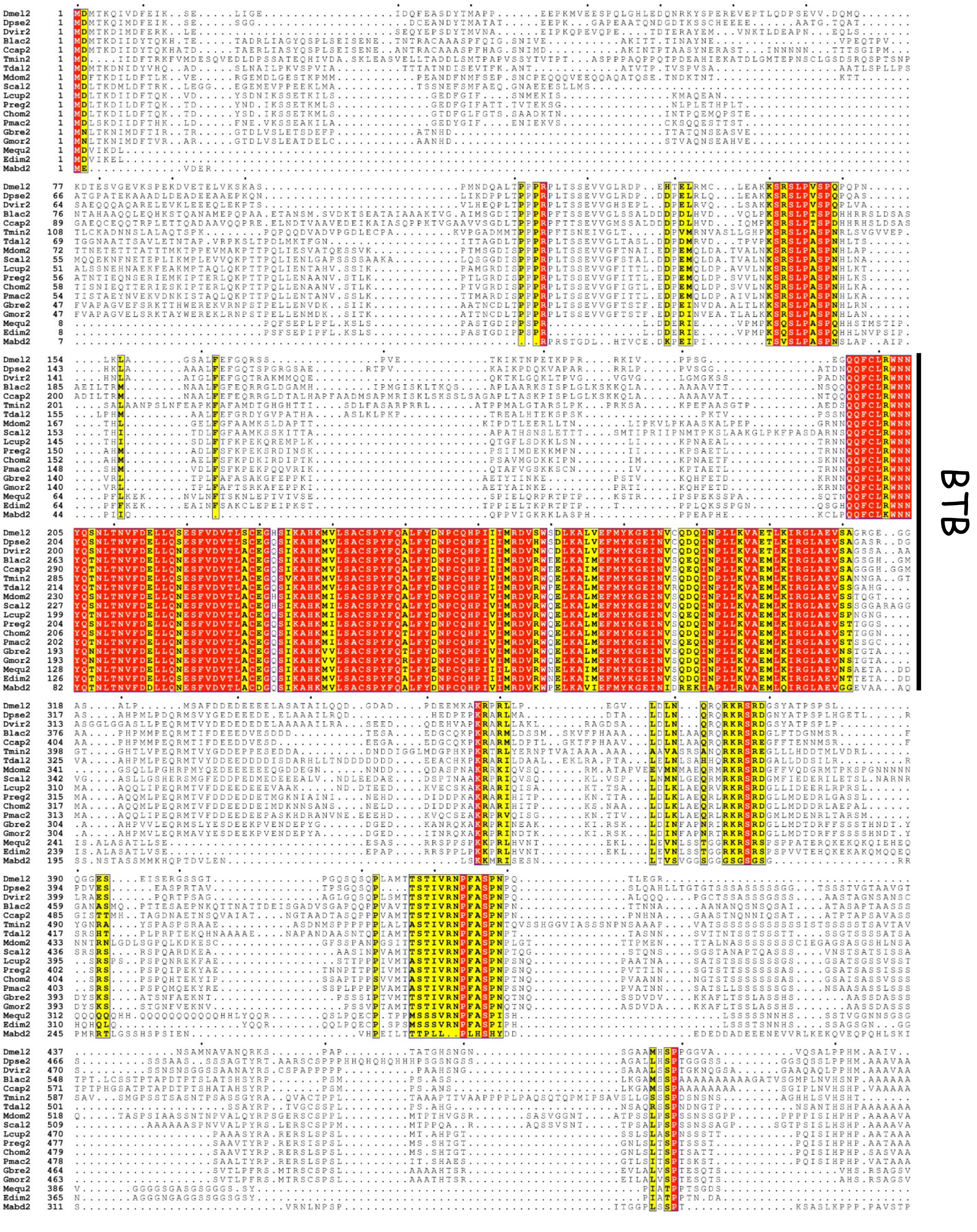

Bab2 sequence conservation among cyclorrhaphans (Part2)

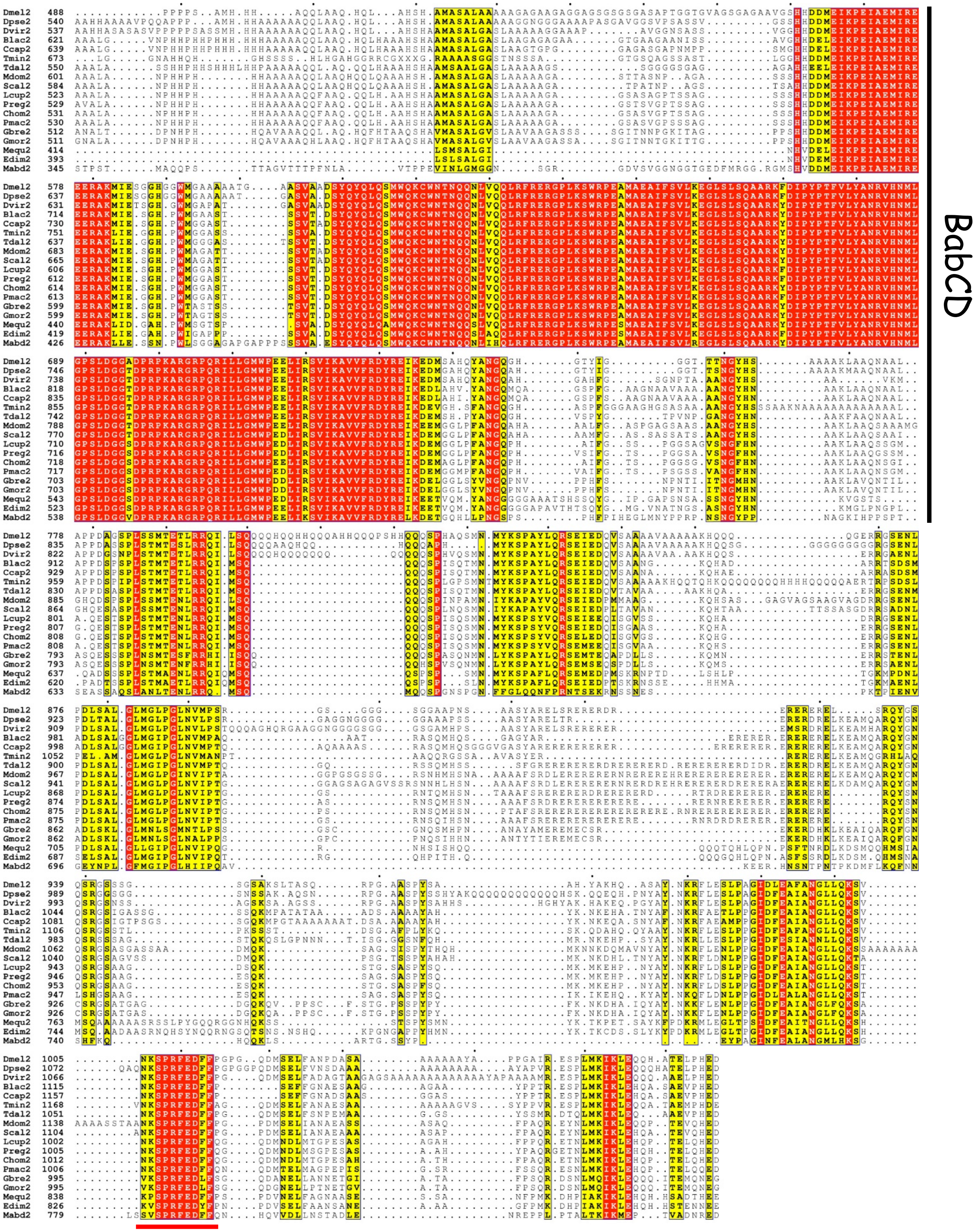

Sequence conservation between Bab1/2 paralogs (Part1)

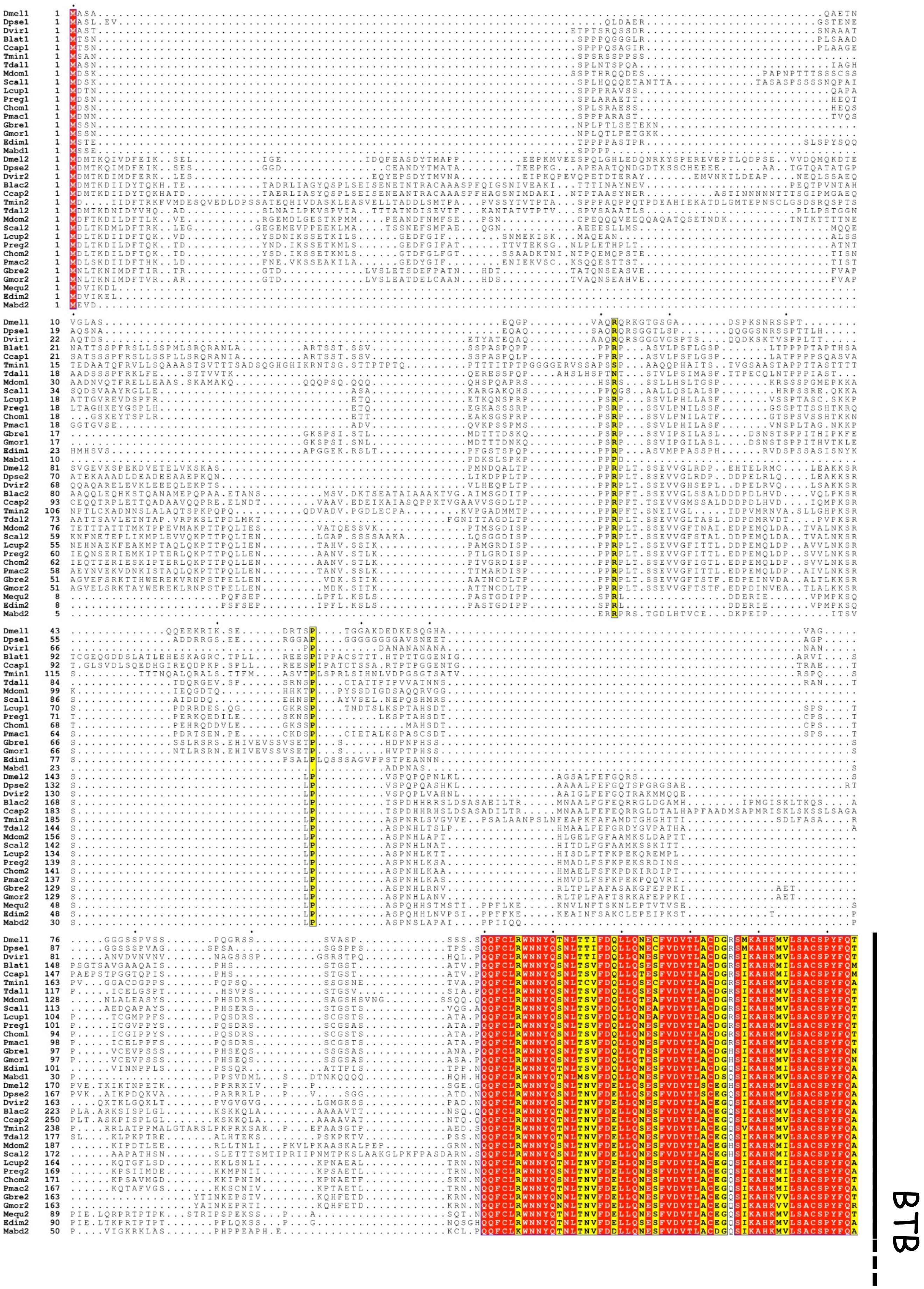

Sequence conservation between Bab1/2 paralogs (Part2)

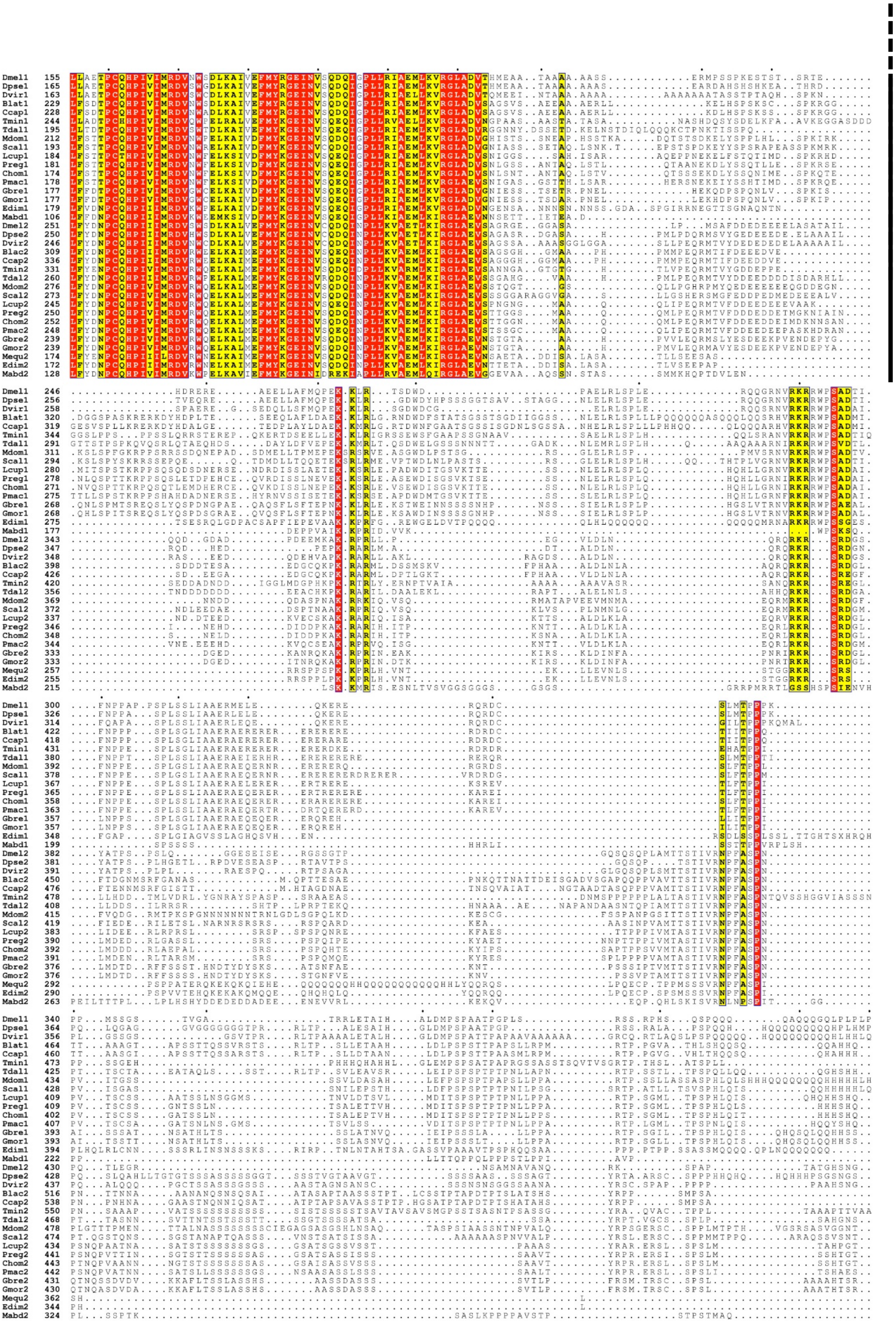

Sequence conservation between Bab1/2 paralogs (Part3)

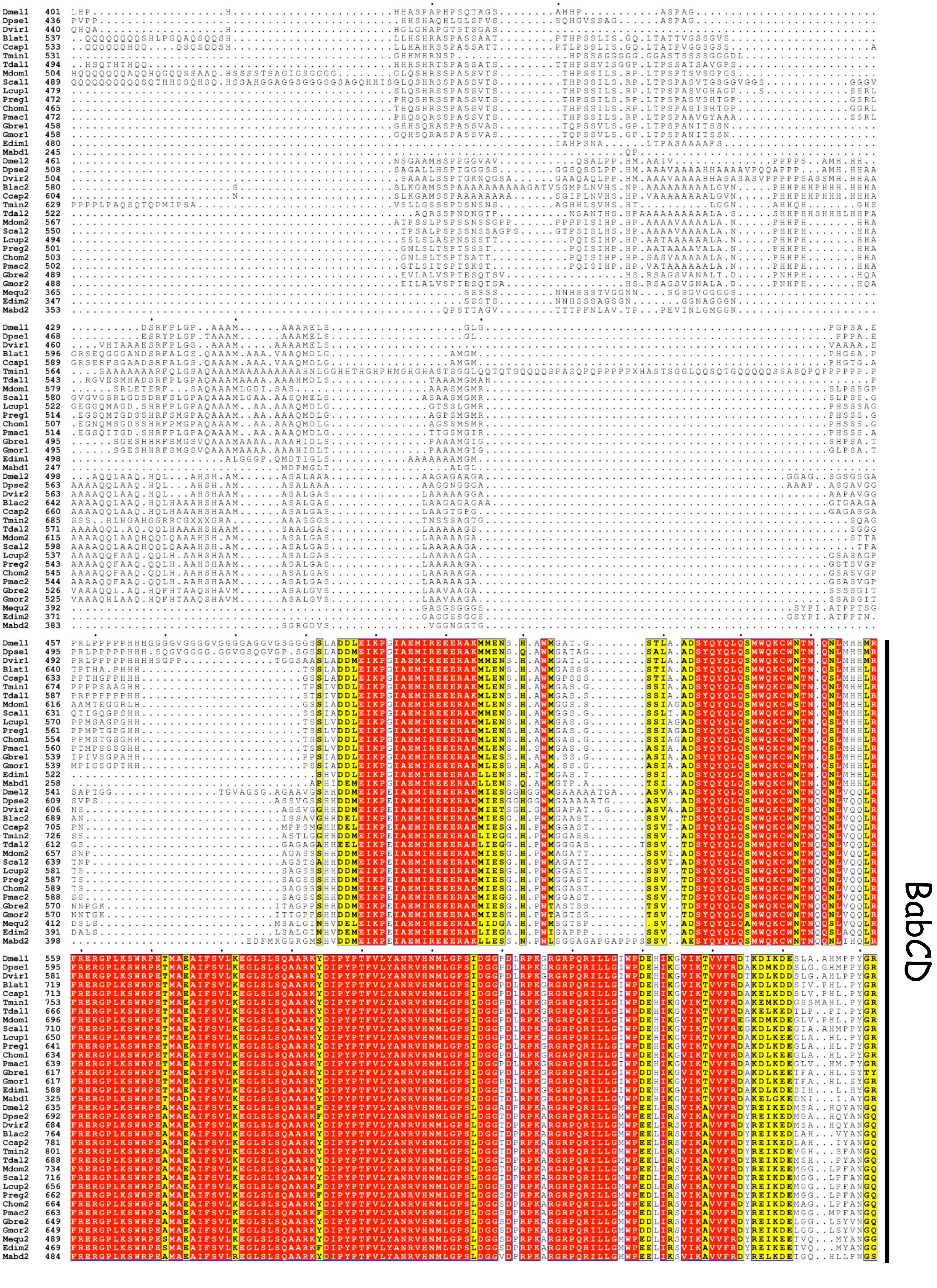

Sequence conservation between Bab1/2 paralogs (Part4)

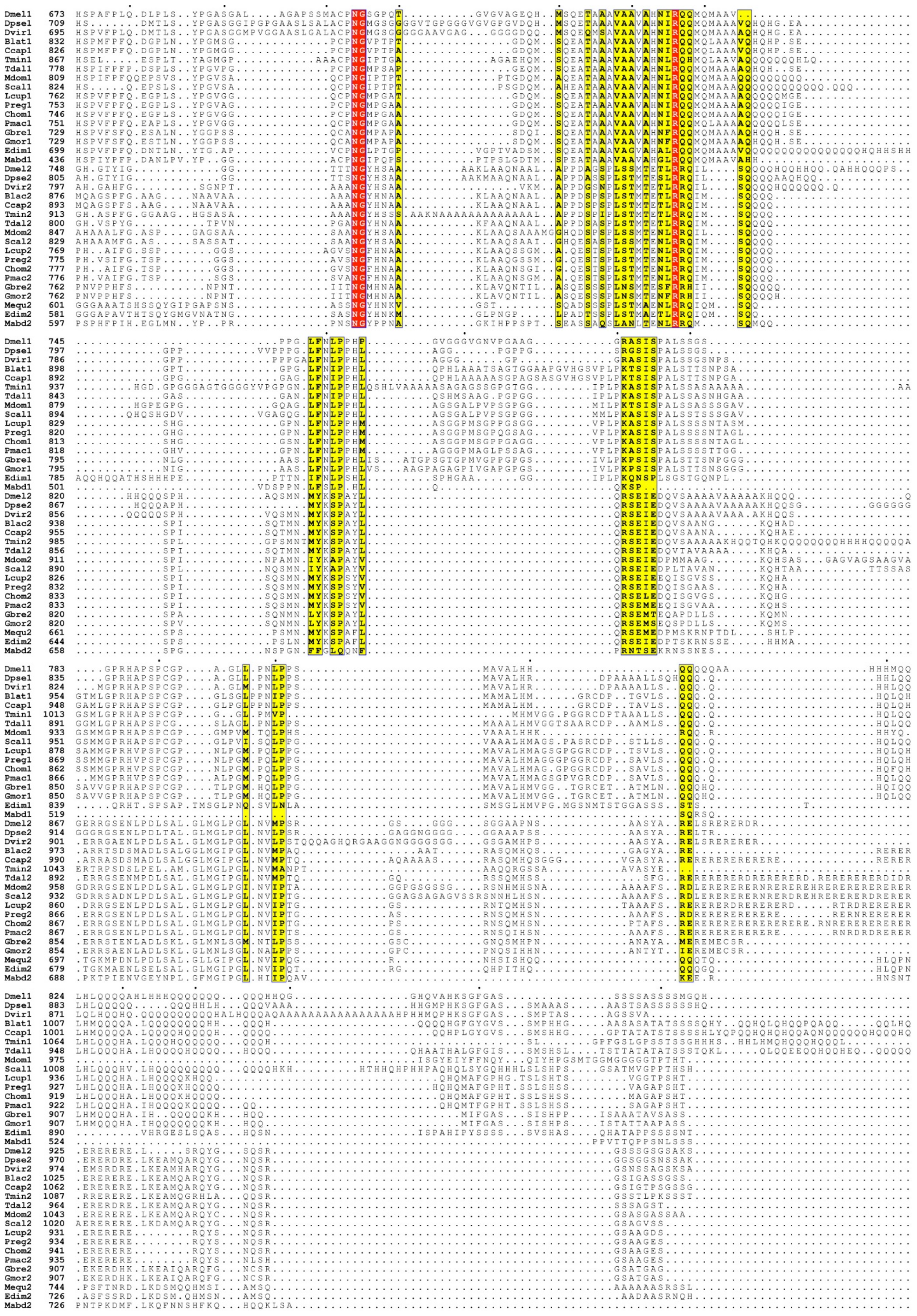

Sequence conservation between Bab1/2 paralogs (Part5)

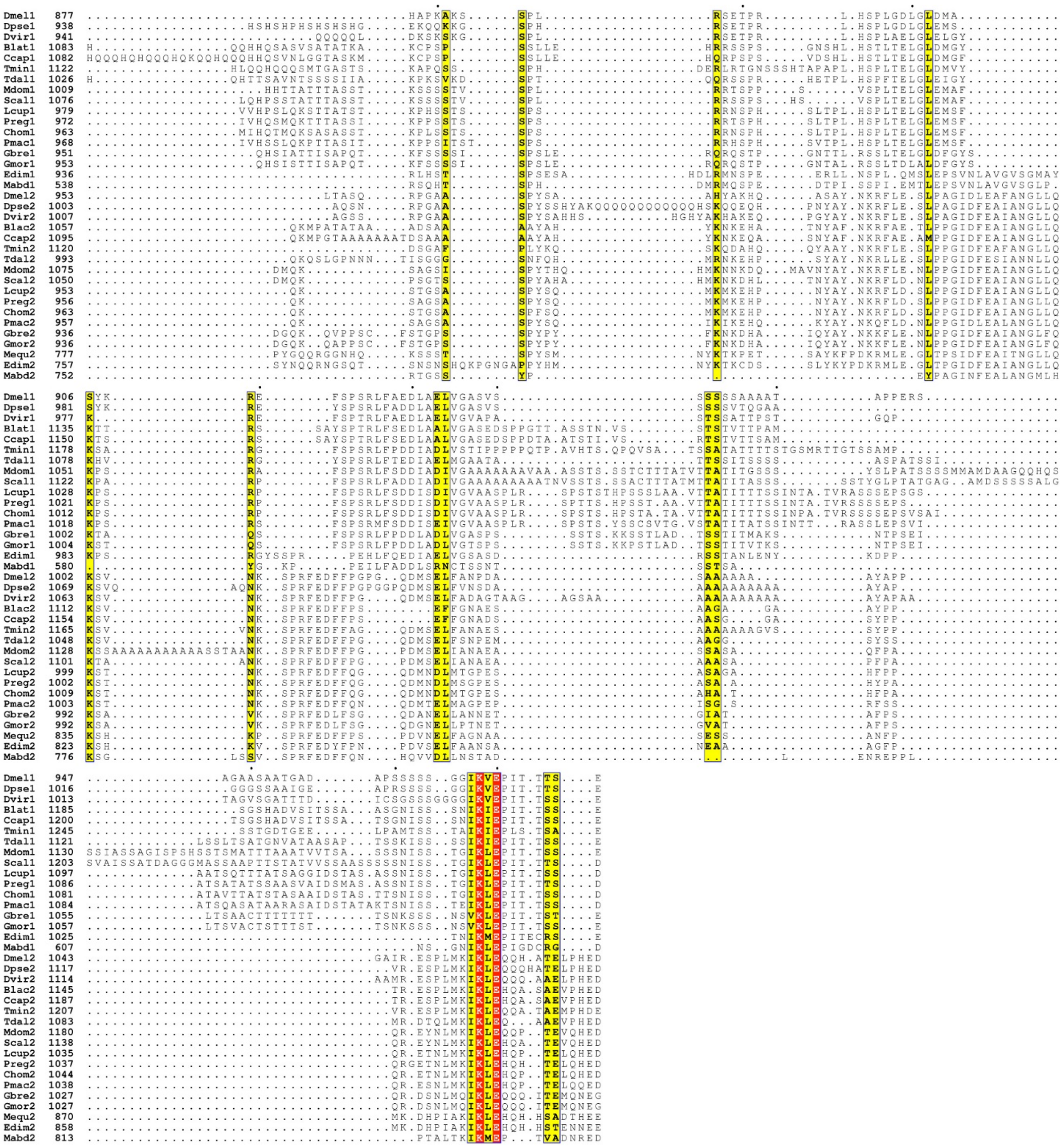

LAE sequence conservation among Drosophilidae (Part1)

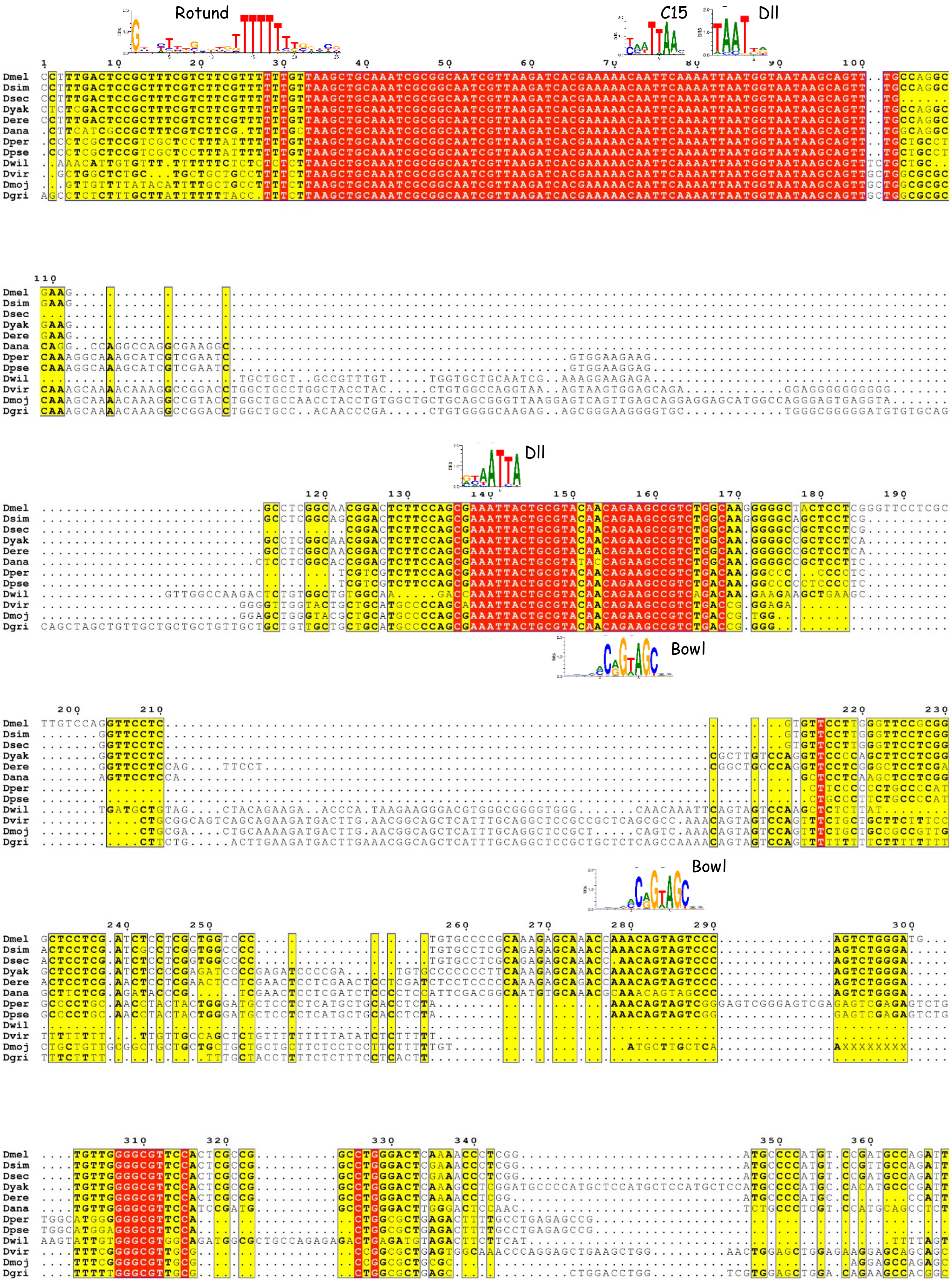

LAE sequence conservation among Drosophilidae (Part2)

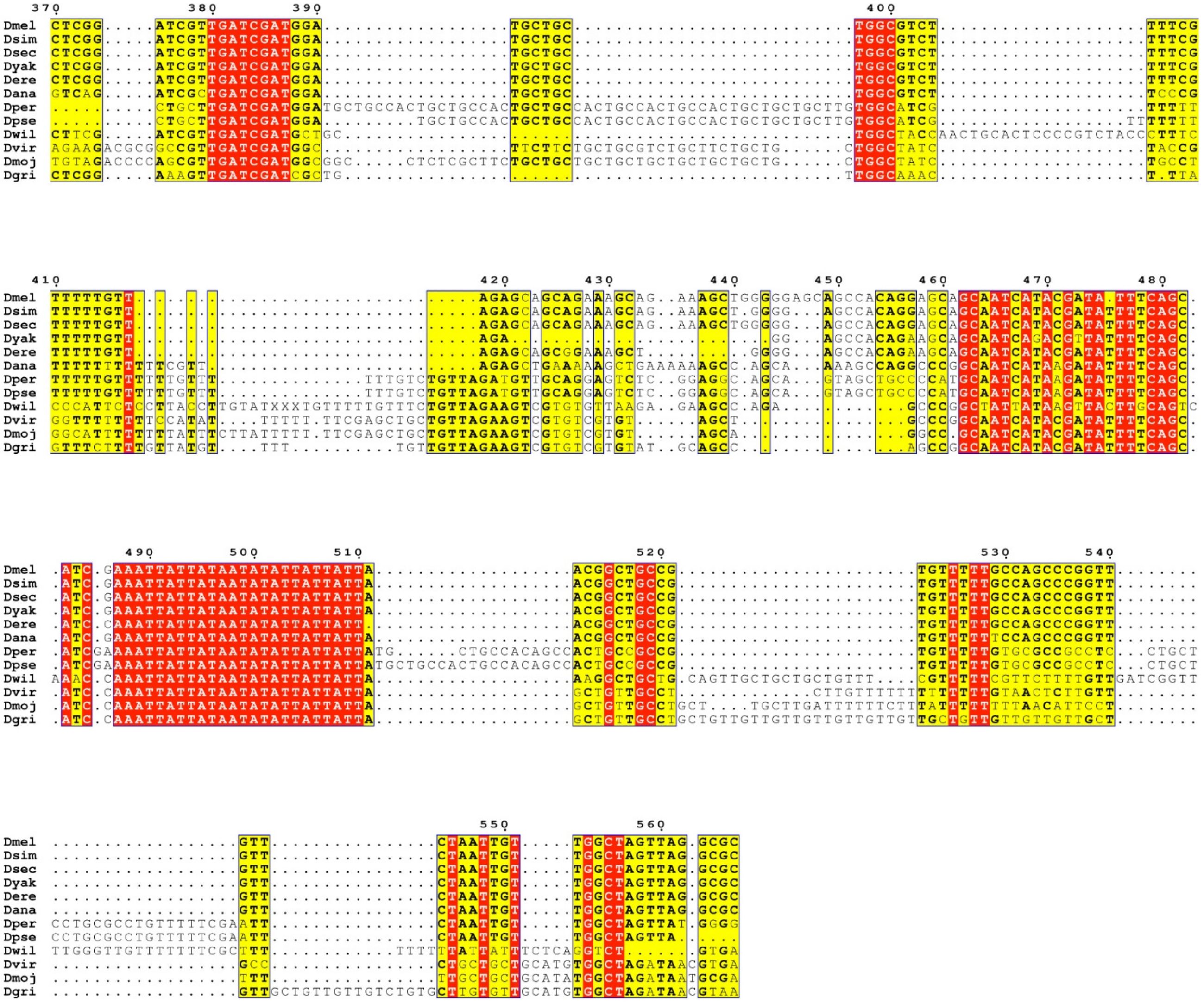

Cardiac CE sequence conservation among Drosophilidae (Part1)

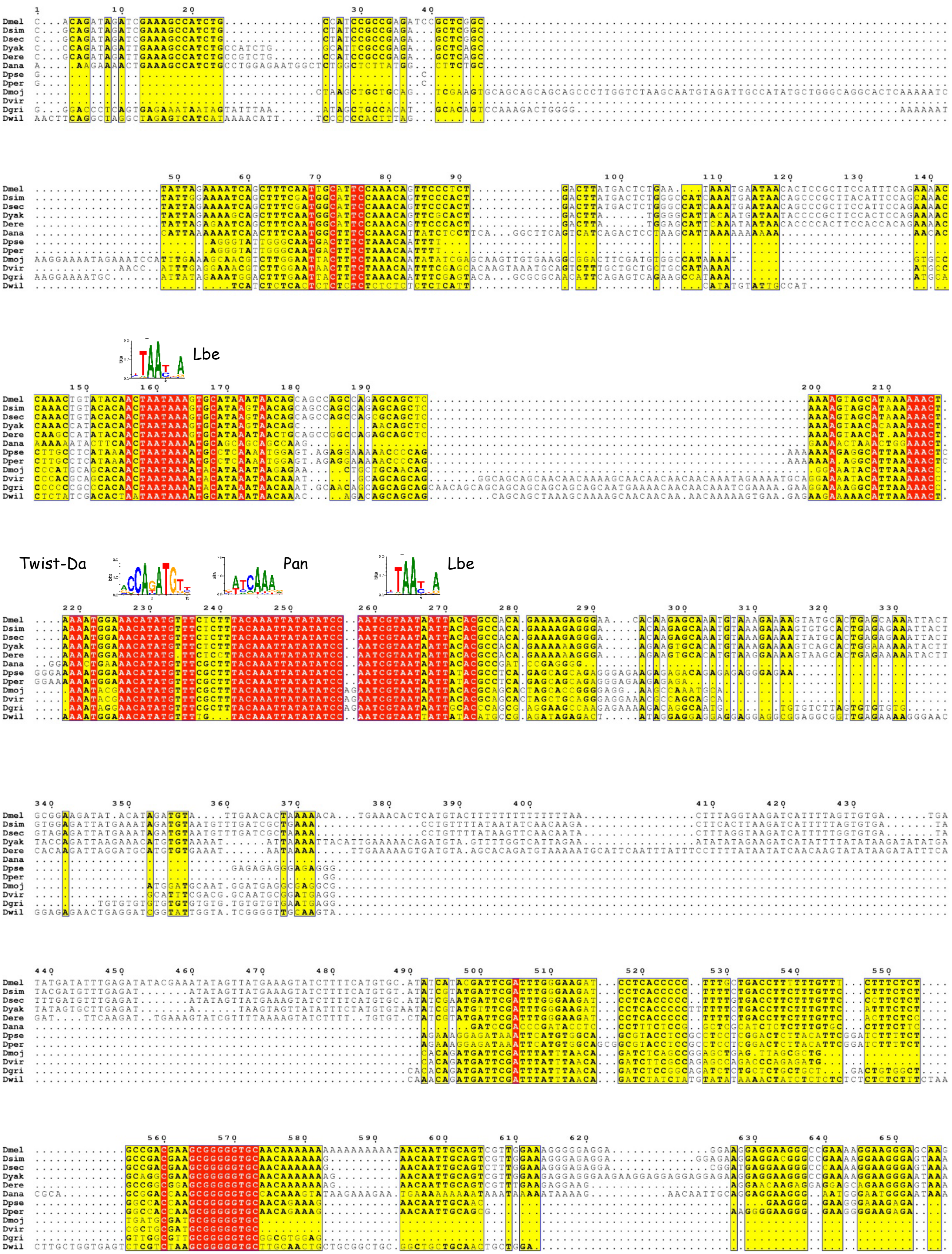

Cardiac CE sequence conservation among Drosophilidae (Part2)

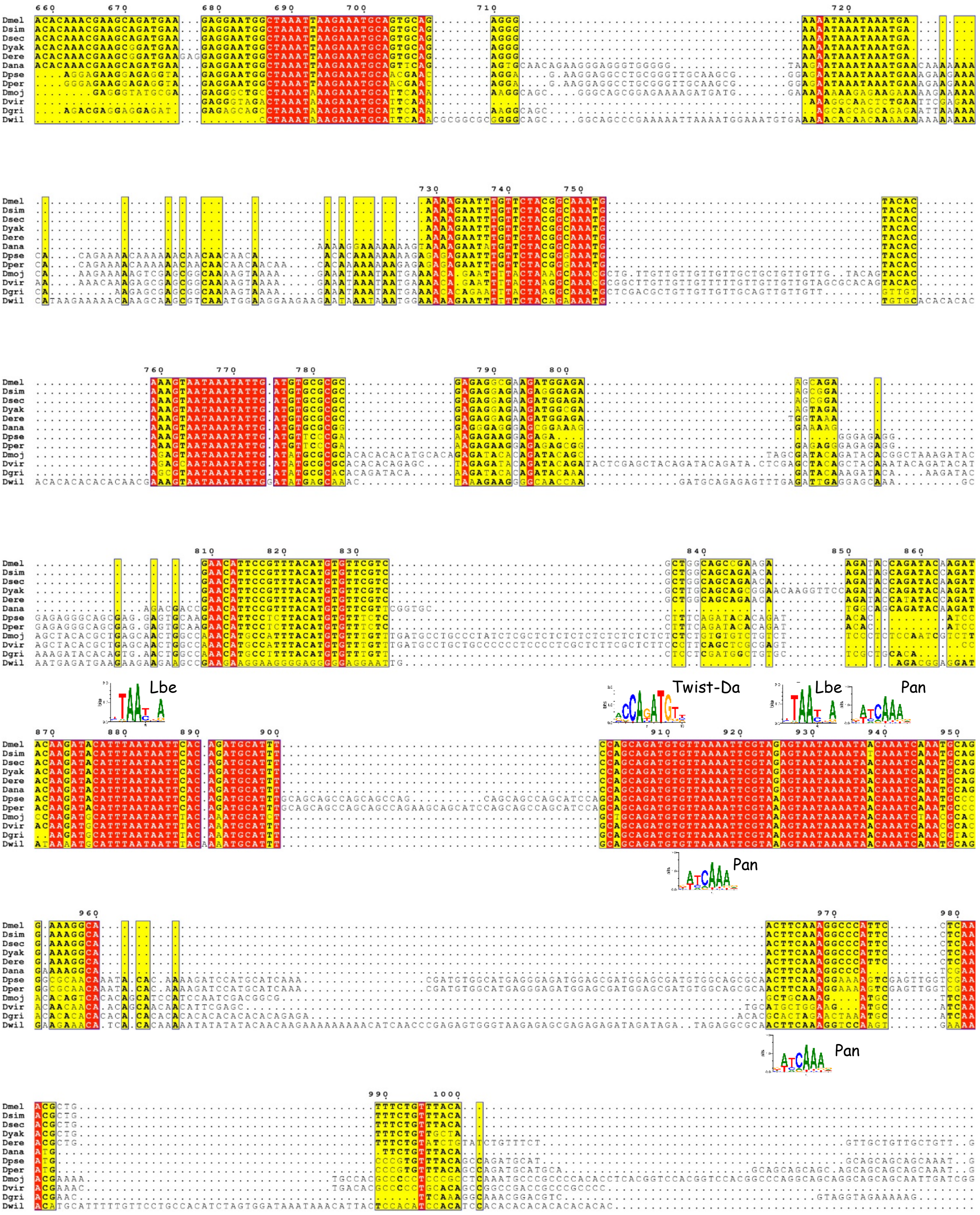

Abdominal AE sequence conservation among Drosophilidae (Part1)

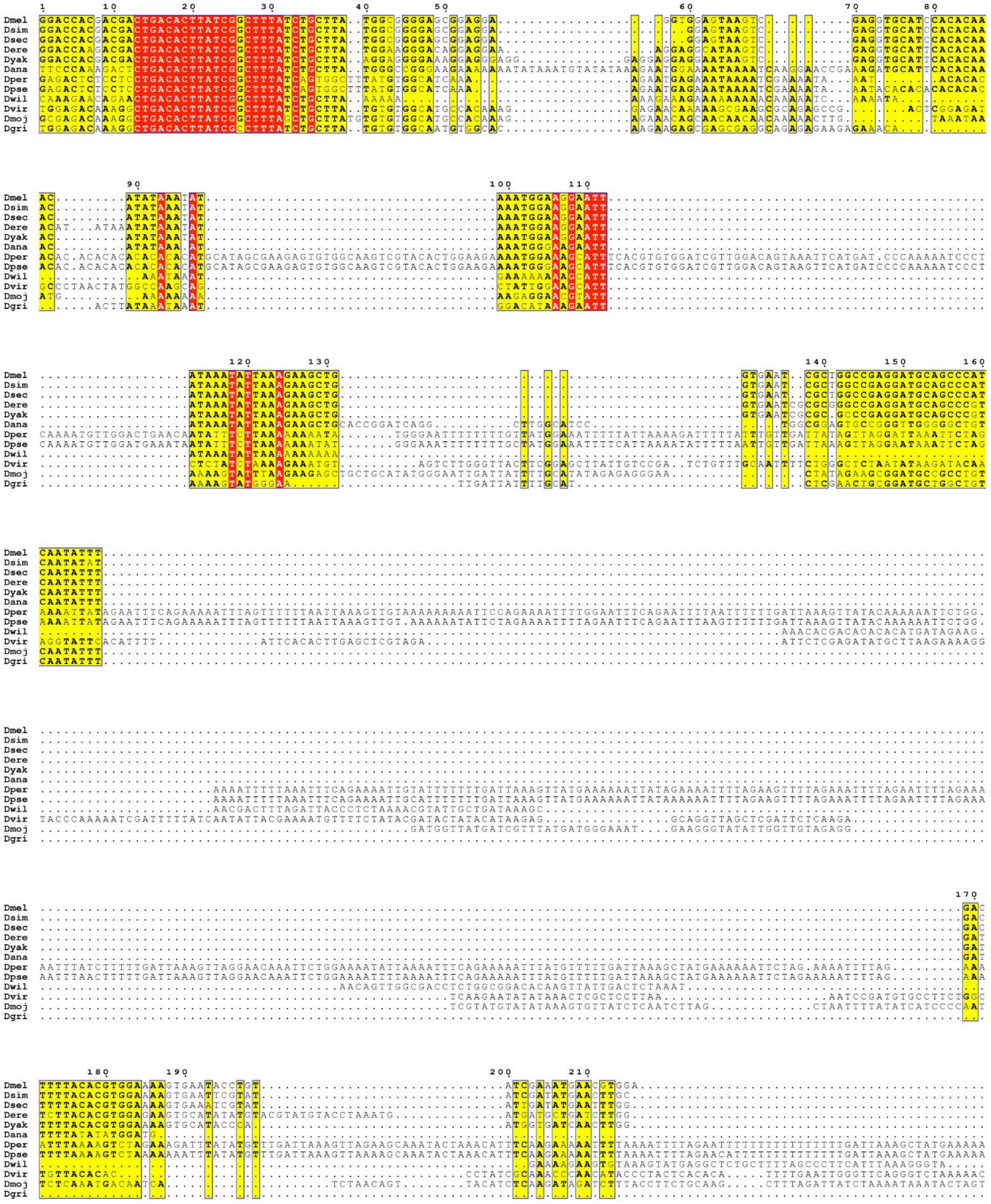

Abdominal AE sequence conservation among Drosophilidae (Part2)

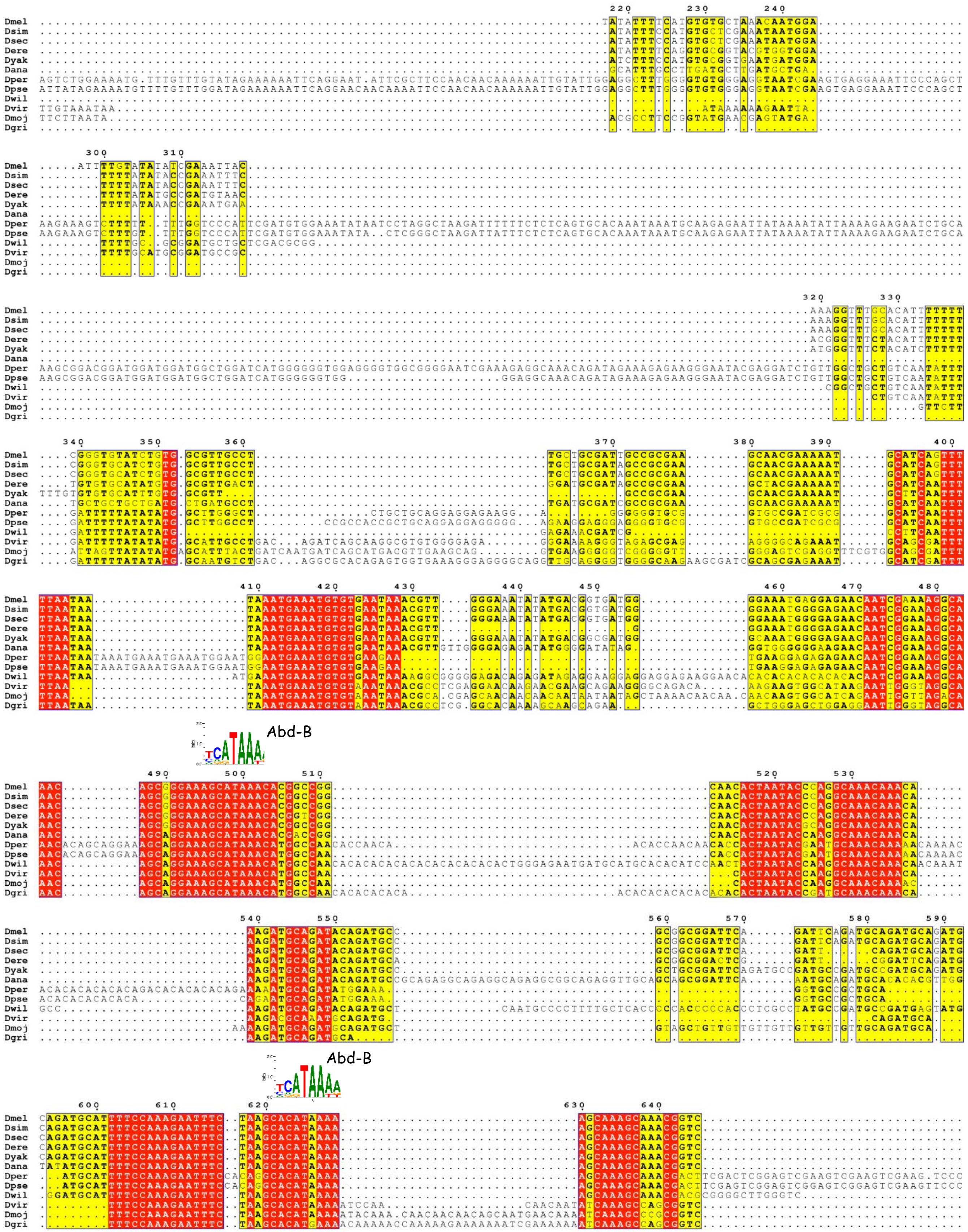

Abdominal AE sequence conservation among Drosophilidae (Part3)

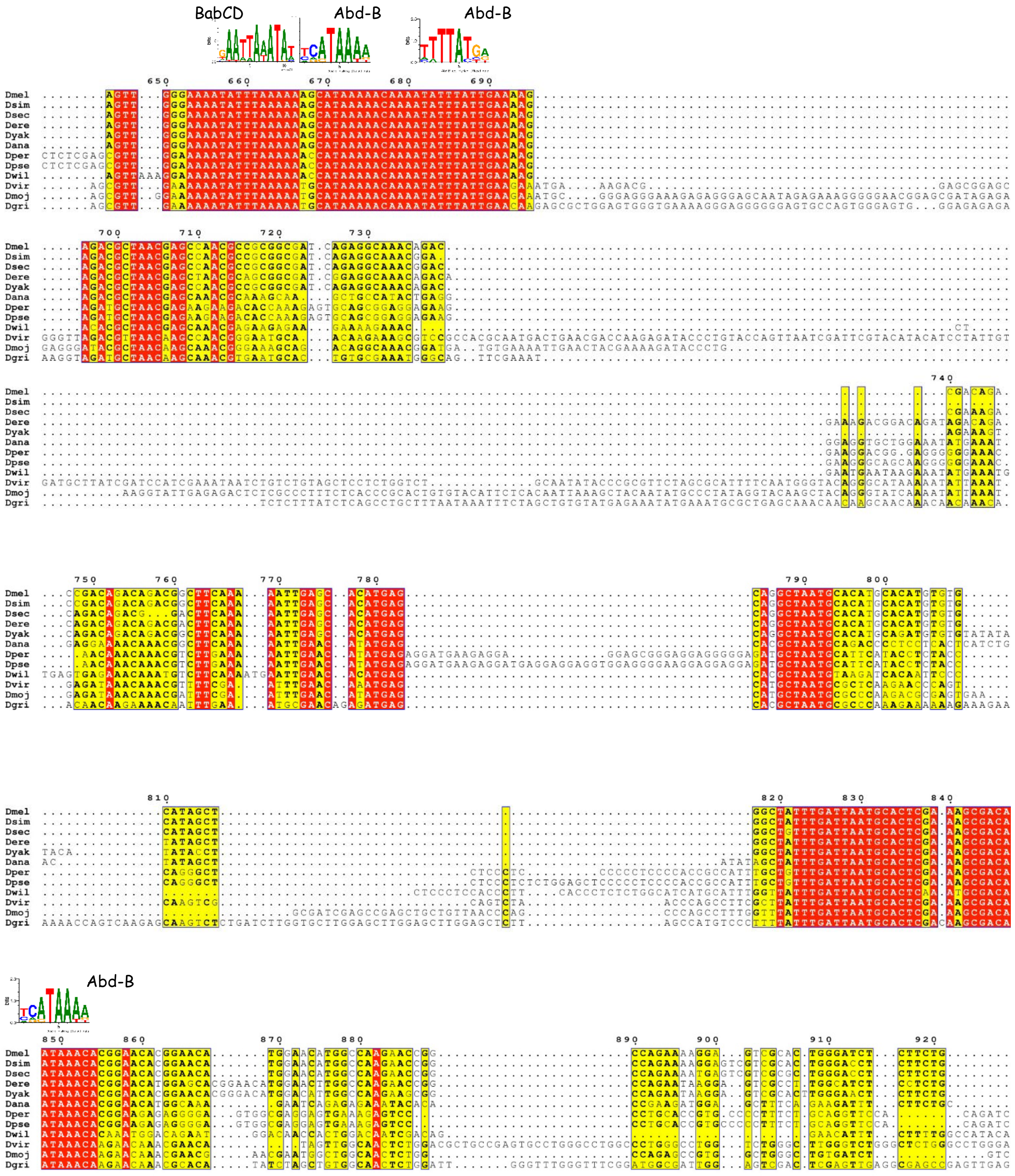

Abdominal AE sequence conservation among Drosophilidae (Part4)

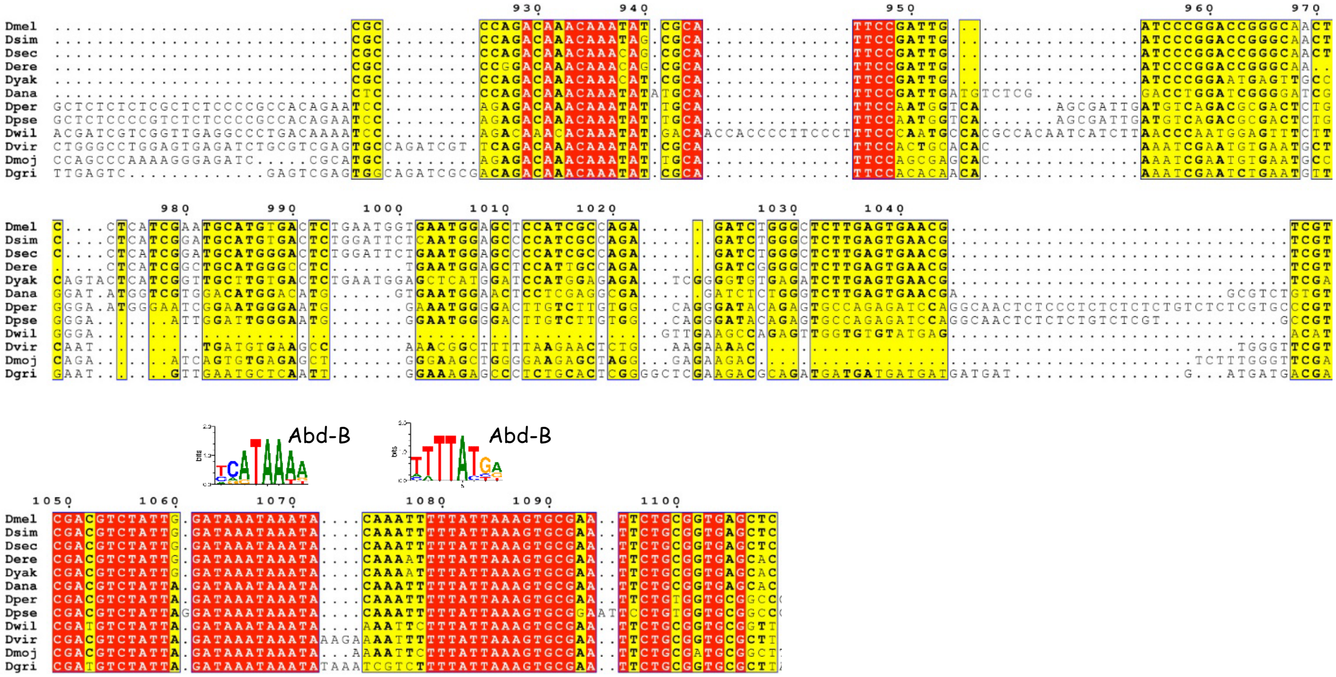

Abdominal DE sequence conservation among Drosophilidae (Part1)

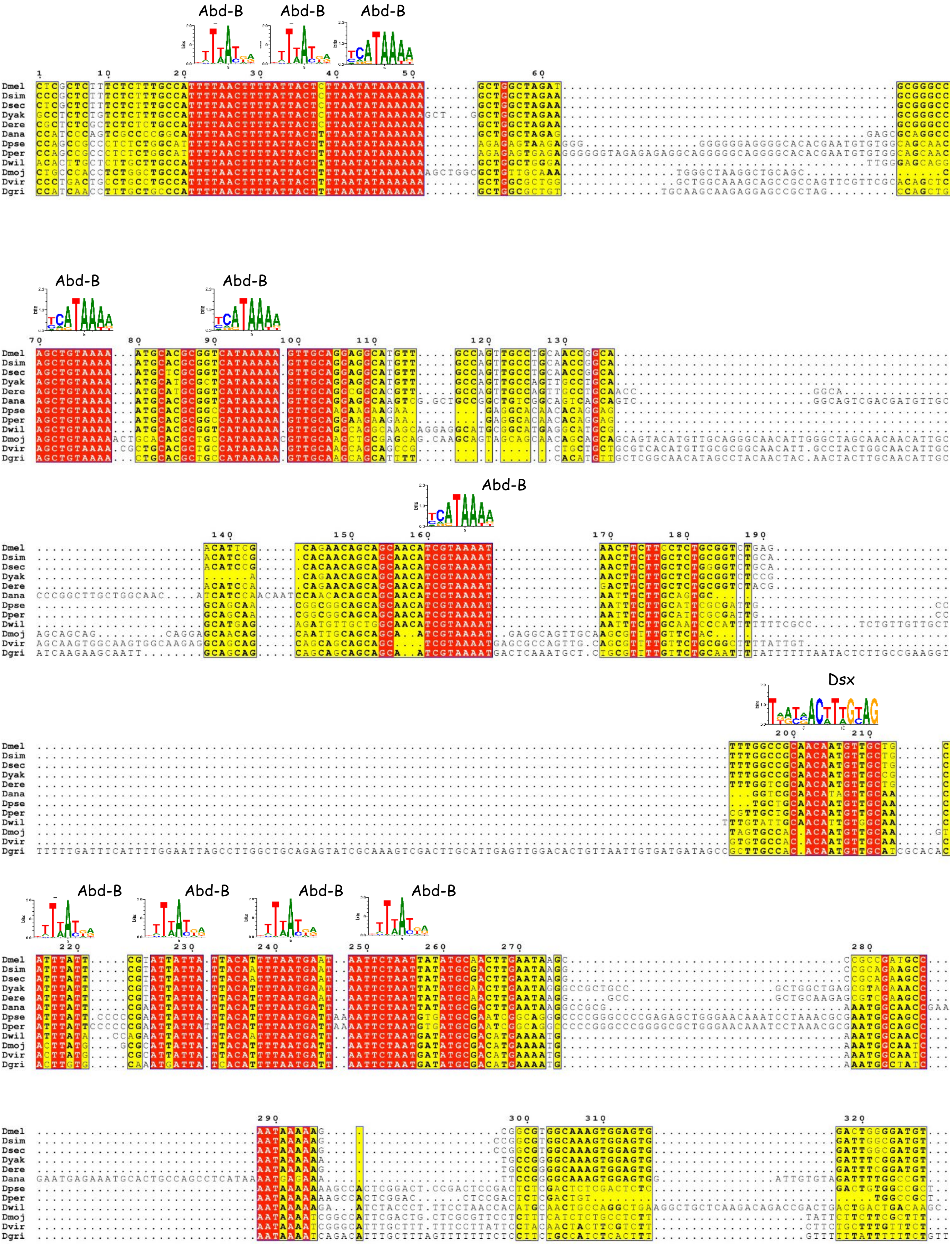

Abdominal DE sequence conservation among Drosophilidae (Part2)

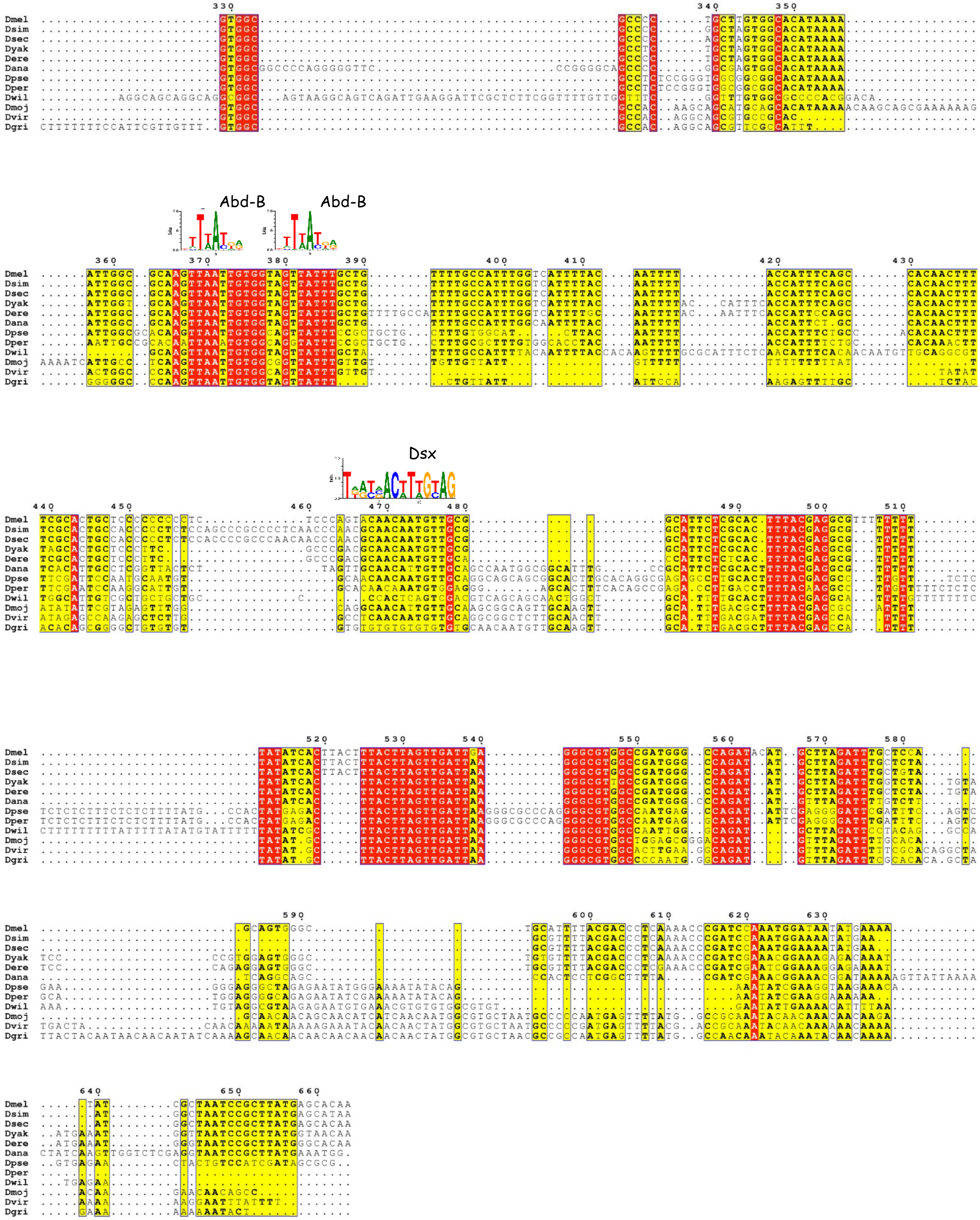

Supplement: S1 Data — p.3-20. Predicted sequences for BTB-BabCD proteins. p.21-22. Bab1 sequence conservation among cyclorrhaphans. p.23-24. Bab2 sequence conservation among cyclorrhaphans. p.25-29 Sequence conservation between Bab1/2 paralogs Sequence conservation between paralogous Bab1/2 proteins among cyclorrhaphans. The four-letter species abbreviations are as listed above (p.2). Strictly conserved amino-acid residues are indicated by white characters on a red background while partially conserved ones are in black characters on a yellow background. Locations of the strongly-conserved BTB and BabCD domains are indicated along the right side (see black lines). p.30-39 Enhancer sequence conservation among Drosophilidae. Conservation among twelve reference drosophilids of D. melanogaster LAE, CE, AE and DE sequences. The four-letter Drosophilidae species abbreviations are as listed below (page 2). Sequence LOGOs of (predicted) binding sites for the Dll, Bowl, C15, Rn, Pan, Lbe, Twist, Abd-B and Dsx transcription factors are depicted above or below the alignments. (PDF) [file pgen.1010083.s007.pdf]
