# Supplementary material for: A shared ancient enhancer element differentially regulates the bric-a-brac tandem gene duplicates in the developing Drosophila leg
Source: PLoS Genet. 2022 Mar 16;18(3):e1010083. doi: 10.1371/journal.pgen.1010083 (PMC8959175; doi:10.1371/journal.pgen.1010083)
Supplement: Attachment — Submitted filename: response to reviewers 2022 01 31.pdf [file pgen.1010083.s008.pdf]

UNIVERSITE DE TOULOUSE
UPS - UMR CNRS 5070 –
EFS - U1301 INSERM - ENVT
Bâtiment INCERE – 4 BIS AVENUE Hubert Curien
31 100 TOULOUSE
Tel. : 33 (0)5. 34.60.95.01 Fax : 33 (0)5.34.60.95.05

January 31th 2022

From: Dr Muriel BOUBE – CRHC CNRS
Email: muriel.boube-trey@inserm.fr

Dear Dr Karthiga Ravichandran,

Please find enclosed a new version of our revised manuscript (see below) containing the following modifications:

- Author contribution has been specified
- Legends of row data file have been added
- Funders and grant numbers has been detailed in both places in the submission form
- Financial Disclosure statement has been completed in full sentences

Thanking you in advance for your consideration in the reviewing process.

Sincerely yours,

Muriel Boube

January 24th 2022

From: Dr Muriel BOUBE – CRHC CNRS
Email: muriel.boube-trey@inserm.fr

Dear Dr Ayrton Kopp,

This letter accompanies our revised version of the manuscript, entitled 'A shared ancient enhancer element differentially regulates the bric-a-brac tandem gene duplicates in the developing Drosophila leg' submitted to PLOS Genetics.

We thank the three reviewers for carefully reading our manuscript and for their constructive comments. To address concerns raised by the Reviewers, three major changes have been done: we performed new qRT-PCR experiment to add several internal controls, the leg phenotypes shown in S2 Fig has been transferred in Figure 2 in order to be present in the main article and phylogenetic relationships have been modified in the figure and the manuscript as recommended by Reviewer 2.

This revised version of our manuscript takes into account all the Referees' requests, which have significantly improved the present manuscript. Please find below a point-by-point reply to the Reviewers and a detailed description of figure and reference modifications.

Reviewer 1 notes a substantial improvement of the revised manuscript and does not require further modifications. He finds that our conclusions overall are well supported by substantial and compelling observations and provide important new insights into the origin and regulation of duplicated genes".

Rewiewer 2 finds the work very interesting and very original given the use of different approaches and thinks it fully deserves publication in PLOS Genet after minor revisions:

- Line 70-73 As suggested, we added that phenotypic novelties during evolution can also originate from protein sequence modification.
- Line 75-76 We agree with reviewer 2 and mentioned in the introduction other examples of Drosophila gene complexes shown to be regulated by shared enhancers.
- Line 84 We replaced" histobast nest" by "larval epidermis"
- Line 113-117 As requested, we detailed bab1 and *bab2* loss of function phenotypes on leg development
- New qRT-PCR experiment have been performed with several control genes. Fig S2C has been replaced by a graph in FigS2B showing *bab1* and *bab2* mRNA quantification relative to 3 control housekeeping genes, *Rpl32*, *Gpdh1* and *Mlc-c* using the ΔΔCt method from Bio-Rad CFX Manager 3.1 software quantification (61).
- Scale bar of 10kb has been added in fig 3
- We agree with Reviewer 2 that the LAE sequences have been fixed in the Cyclorrhapha instead of the Muscomorpha lineage and modified accordingly the text and the figures.
- New phylogenic tree has been extracted from published dipteran or dipteran sub-groups phylogenies (Wiegmann et al 2011, Vicoso 2015, Wiegmann et al 2018) and Fig 5B. and S3A Fig panels have been replaced by a new panel in Fig 5B.
- The phylogenetic reconstruction method has been detailed in the material and method. Phylogenetic relationships were inferred through a maximum likelihood analysis with W-IQ-Tree (http://iqtree.cibiv.univie.ac.at/), using JTT+F+R6 as a substitution model, and visualized with the ETE toolkit (http://etetoolkit.org/treeview/)

- Line 302-306 In the phylogeny presented in Fig 5B, *bab1* and *bab2* clades do not group together which is consistent with an old duplication and a faster evolution of *bab2* genes. This interpretation is further discussed in the text according to reviewer 2 queries.
- As recommended by reviewer 2, the leg phenotype with the ectopic sex comb of the LAE deleted flies (ex S2Fig) has been included in Figure 2 in order to be present in the main article.

Rewiewer 3 thinks that our work provides interesting new insights into the mechanisms by which new regulatory linkages are formed during gene duplication and is appropriate for publication in Plos Genetics it its current format with two minor comments:

- Fig legends has been changed to Times or arial according to journal file figure requirement
- Line 395: As recommended, “This work brings some clues…” has been changed to “This work raises some hypotheses”

Figure changes

Fig 1 and 4 are unchanged

Panel B of Fig S3 with ΔLAE leg phenotypes has been moved to principal Figure 2B

Phylogenic trees in Fig5, Fig 6B, and S3AFig have been modified or deleted according to reviewer 2 concerns.

Muscomorpha subgroup has been replaced by cyclophorra infra order all along the text and in Fig 5,6, S4, S6 and in sup data p21, p23 and p25

Added references (see reviewer 2) :

=> Concerning regulation of other drosophila duplicated genes:

11. Cheng Y, Brunner AL, Kremer S, DeVido SK, Stefaniuk CM, Kassis JA. Co-regulation of invected and engrailed by a complex array of regulatory sequences in Drosophila. Dev Biol. 2014;395(1):131-43.

12. Barrio R, de Celis JF, Bolshakov S, Kafatos FC. Identification of regulatory regions driving the expression of the Drosophila spalt complex at different developmental stages. Dev Biol. 1999;215(1):33-47.

13. Nellesen DT, Lai EC, Posakony JW. Discrete enhancer elements mediate selective responsiveness of enhancer of split complex genes to common transcriptional activators. Dev Biol. 1999;213(1):33-53.

14. Gómez-Skarmeta JL, Rodríguez I, Martínez C, Culí J, Ferrés-Marcó D, Beamonte D, et al. Cis-regulation of achaete and scute: shared enhancer-like elements drive their coexpression in proneural clusters of the imaginal discs. Genes Dev. 1995;9(15):1869-82.

=> Concerning dipteran phylogeny:

46. Wiegmann BM, Trautwein MD, Winkler IS, Barr NB, Kim JW, Lambkin C, et al. Episodic radiations in the fly tree of life. Proc Natl Acad Sci U S A. 2011;108(14):5690-5.

47. Wiegmann BM, Richards S. Genomes of Diptera. Curr Opin Insect Sci. 2018;25:116-24.

48. Vicoso B, Bachtrog D. Numerous transitions of sex chromosomes in Diptera. PLoS Biol. 2015;13(4):e1002078.

=> Concerning qPCR RNA quantification with multiple internal control genes

61. Vandesompele J, De Preter K, Pattyn F, Poppe B, Van Roy N, De Paepe A, et al. Accurate normalization of real-time quantitative RT-PCR data by geometric averaging of multiple internal control genes. Genome Biol. 2002;3(7):RESEARCH0034.

Thanking you in advance for your consideration in the reviewing process.

Sincerely yours,

Muriel Boube

dépasser les frontières
Université Paul Sabatier TOULOUSE III
Instituts thématiques Inserm
Institut national de la santé et de la recherche médicale
EFS
